# Engineering In Situ Loose Selective Interface with Conducting Channels for Practical Ah-Level Aqueous Zinc Metal Batteries

**DOI:** 10.1007/s40820-026-02264-y

**Published:** 2026-07-08

**Authors:** Dongdong Wang, Shuo Dou, Qiongying Huang, Jiaying Li, Mi Xu, Jingshuai Li, Zhendong Guo, Siqi Qin, Tieyan Wang, Haipeng Yu, Haozhen Dou, Zhongwei Chen

**Affiliations:** 1 State Key Laboratory of Utilization of Woody Oil Resource, Key Laboratory of Bio-Based Material Science and Technology of Ministry of Education, Northeast Forestry University, Harbin, 150040 People’s Republic of China; 2 Power Battery & Systems Research Center, State Key Laboratory of Catalysis, Dalian Institute of Chemical Physics, Chinese Academy of Sciences, Dalian, 116023 People’s Republic of China

**Keywords:** Aqueous zinc metal batteries, Porous organic cages, Loose selective interface, Molecular engineering

## Abstract

In situ construction of highly conductive and self-repairing electrode interface with porous materials remains a grand challenge for practical aqueous batteries. Herein, loose selective interface (LSI) is in situ constructed by self-assembly of porous organic cages (POCs) for practical Ah-level aqueous zinc metal batteries. The delicate balance between hydrogen-bonding interactions of POC-POC and coordination interactions of POC–anion/cation endows LSI with loose porous structure, selective Zn^2+^-conducting channels and dynamic self-repairing capability. LSI obtains the high Zn^2+^ transfer number of 0.8, fast desolvation kinetics and homogeneous electric field distribution, which induces the dendrite-free Zn deposition along the Zn (002) plane. Meanwhile, LSI effectively confines the activity of interfacial water and enhances interfacial hydrophobicity, thus inhibiting the water-induced side reactions. Notably, Zn//Zn cells deliver the cycling life over 3200 cycles at 50 mA cm^−2^ and Zn//NVO full cells stably perform over 10,000 cycles at 10 A g^−1^. Moreover, Ah-level pouch cell with high cathode areal capacity of 7.25 mAh cm^−2^ and limited* N*/*P* of 2.65 delivers the initial discharge capacity of 1 Ah and cycling stability for over 237 cycles. This study offers a novel methodology for constructing stable highly conductive interfaces with porous materials.

**Supplementary Information:**

The online version contains supplementary material available at 10.1007/s40820-026-02264-y.

## Highlights

- A loose selective interface (LSI) is in situ constructed by self-assembly of porous organic cages for aqueous Zn metal batteries.
- The porous LSI enables fast Zn^2+^ transport, dynamic self-repairing capability and dendrite-free Zn deposition.
- Ah-level aqueous Zn pouch cells deliver 1 Ah capacity with stable cycling performance.

**Supplementary Information:**

The online version contains supplementary material available at 10.1007/s40820-026-02264-y.

## Graphical abstract

**Figure:**
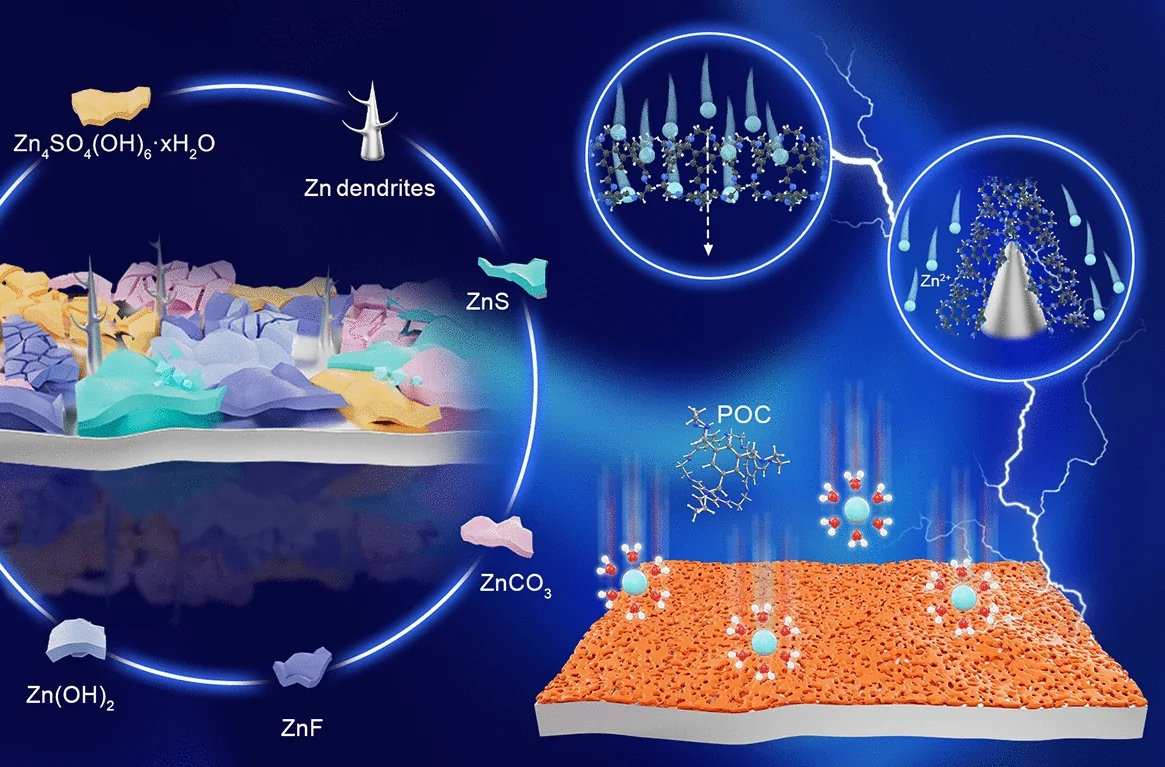

## Introduction

The integration of renewable energy sources such as wind and solar into the grid is efficient a path toward the low-carbon and sustainable energy structure, where the intermittency of renewable energy poses high requirements for battery energy storage technology [1–3]. Although lithium-ion batteries have achieved great success for renewable energy storage, their drawbacks of limited lithium resources and operation safety risks have driven the search for next-generation batteries [4–6]. Rechargeable aqueous Zn metal batteries (AZMBs) that operate in neutral or mildly acidic aqueous electrolyte have emerged as a promising alternative due to their exceptional safety, cost-effectiveness, and environmental benignity [7–12]. Particularly, Zn metal anode offers the highly appealing advantages such as high theoretical capacity, low redox potential and abundant reserves [13–15]. However, the commercialization of AZMBs is seriously hindered by interface issues associated with Zn anode due to the absence of stable electrode/electrolyte interface, which results in short cycle life and low Coulombic efficiency (CE) [12]. First, the high thermodynamic activity of interfacial water triggers the severe hydrogen evolution reaction (HER) and formation of by-products [16, 17]. Moreover, sluggish interfacial desolvation kinetics and uneven Zn^2+^ diffusion/deposition induce the formation Zn^2+^ depletion zones and severe dendrite growth [18].

Constructing the versatile anode interfaces via ex situ or in situ methodology is widely regarded as the most straightforward and effective strategy to address these issues [19, 20]. Artificial interfaces constructed by ex situ coating of inorganic nanomaterials or organic polymers on Zn anode can prevent the direct contact between aqueous electrolyte and Zn anode, thus relieving side reactions [21–23]. Nevertheless, most of artificial interfaces require the complicated fabrication procedure and bear the risk of exfoliating from the Zn anode under the repeated plating/striping. In this circumstance, in situ method has been conducted to spontaneously form solid electrolyte interface (SEI) via three main mechanisms of chemical reaction, electrochemical reaction or self-assembly, which have notable advantages of simple operation and can dynamically repair interface [24–27]. For example, Guo et al. have designed a Zn^2+^-conductive SEI via the chemical reaction between Zn(H_2_PO_4_)_2_ and OH^−^ to effectively promote the reversibility of Zn anode [28]. Yang et al. have designed anion-involved solvation shell by adding bidentate coordination cosolvent and constructed organic–inorganic bilayer SEI via the electrochemical decomposition of organic solvent or anion [29]. Zhou et al. have built a self-assembled multilayer interface via the self-assembly of L-cysteine, which alleviates the dendrite growth and side reactions [30]. However, in situ SEI generally encounters challenges such as poor ion conductivity, deteriorated desolvation kinetics, or compromise in safety and cost of aqueous electrolytes. Moreover, the cycling stability of AZMBs still needs to be further improved, especially under practical operations condition of high current density (> 10 mA cm^−2^), large areal capacity (> 5 mAh cm^−2^) or Ah-level pouch cell (> 1 Ah). Therefore, it is imperative to develop new interface construction strategy for developing practical AZMBs.

Porous materials such as metal–organic framework (MOFs) [31], covalent organic framework (COFs) [32–34], porous aromatic frameworks (PAFs) [35–37], polymers of intrinsic microporosity (PIM) [38] and POC have the attributes of large porosity, well-defined pore structures, controllable pore sizes, and tunable functional groups, which endow them as leading material platform for designing high-performance batteries [39–43]. Currently, porous materials can effectively tailor solvent activity, metal cation solvation structure as well as its interfacial desolvation and diffusion, which are widely used as building blocks for advanced separator, artificial interface layer or solid-state electrolytes [44]. However, the implementation of porous materials for in situ SEI has rarely been reported. Among porous materials, POC exhibits the unique host–guest chemistry and solution processability, which affords high compatibility with liquid electrolytes and great potential for constructing in situ SEI via manipulation their intermolecular forces [45]. Previous studies have primarily focused on incorporating POCs into bulk electrolytes to construct quasi-solid-state electrolytes (QSSEs), where their ordered channels and cavity-induced anion-trapping effects contribute to enhanced ionic conductivity and cation transference numbers. In such systems, POCs mainly function as structural components within the electrolyte matrix, aiming to optimize bulk ion transport and mechanical stability [44]. However, the application of POC as functional additives of aqueous electrolytes for in situ interface design has never been demonstrated, and the structure–performance relationship of POC-derived interface urgently needs to be explored.

In this contribution, we propose a novel methodology to in situ constructs electrode interface with porous materials for practical Ah-level AZMBs. By selecting POC as fictional additive of aqueous electrolytes, LSI with selective Zn^2+^-conducting channels and dynamically self-repairing capacity is in situ constructed by precise manipulation of interfacial self-assembly of POC. As highlighted by the combined experiment and theoretical simulation, the fine-balance between hydrogen-bonding interactions and coordination interactions contributes to the formation of stable LSI, which significantly suppress dendrite growth and side reaction of Zn anode. LSI obtains the high Zn^2+^ transfer number of 0.8, fast desolvation kinetics and homogeneous electric field distribution, which induces the dendrite-free Zn deposition along the Zn (002) plane [46]. Meanwhile, LSI effectively confines the activity of interfacial water and enhances interfacial hydrophobicity, thus inhibiting the water-induced side reactions. Notably, Zn//Zn cell delivers the cycling life over 3200 cycles at 50 mA cm^−2^ and Zn//NVO full cell stably performs over 10,000 cycles at 10 A g^−1^. Moreover, Ah-level pouch cell with high cathode areal capacity of 7.25 mAh cm^−2^ and limited N/P of 2.65 delivers the initial discharge capacity of 1 Ah and cycling stability for over 237 cycles. The pioneering attempt of in situ SEI with ion-conducting channels shed light on the design of advanced interfaces for high-performance batteries.

## Experimental Section

### Materials

Zinc sulfate heptahydrate (ZnSO_4_·7H_2_O, AR), N-methyl-2-pyrrolidone (NMP, 99.0%), ethyl acetate (EA, 99.5%), 1,3,5-triformylbenzene (TFB, 97%), ethylenediamine (EDA, 98%) were purchased from Aladdin reagent Co., Ltd. Zn foils (0.02 and 0.10 mm, 99.9%), Cu foil (0.01 and 0.03 mm, 99.9%), Ti foil (0.03 mm, 99.9%) were provided Qingyuan Metal Materials Co., Ltd. Glass microfiber filter (GF/D and GF/F) was purchased from Whatman Co., Ltd. Ketjenblack (KB) and polyvinylidene difluoride (PVDF) were purchased from Canrd Technology Co. Ltd. All chemical reagents were purchased and used directly without further purification.

### Electrolyte Preparation

ZnSO_4_·7H_2_O (Aladdin, AR) was dissolved into deionized water to prepare 2M ZnSO_4_ electrolyte. Additionally, 2M ZnSO_4_ aqueous electrolyte was recorded as ZS. The electrolytes with different mass-to-volume ratio of POCs powder (5, 10, and 20 mg) were prepared by adding into the 1 mL ZnSO_4_ electrolytes and stirring for 6 h. The prepared ternary eutectic electrolytes were abbreviated as ZS-5POC, ZS-10POC and ZS-20POC. Unless otherwise specified in this document, the optimal dosage is expressed by ZS-POC.

### Synthesis of POCs

POCs was synthesized according to a previous work [47]. Ethyl acetate (35 mL) was added to TFB (50 mg, 0.31 mmol) in a beaker at room temperature. After 5 min, a solution of EDA (28 mg, 0.47 mmol) in EtOAc (5 mL) was added. The resulting mixture was left covered for 72 h without stirring. A turbid solution was observed to form within 5 min after ethylenediamine addition to the partially dissolved trialdehyde. This was followed by precipitation of a solid after around 5–6 h, and finally, pale white needles of cage were observed to crystallize from solution after around 72 h. The product obtained was a mixture of needle-like crystals which adhered to the sides of the reaction flask (major component) and a thin layer of amorphous material at the bottom of the flask (minor component). The crystals were harvested carefully from the sides of the flask using a spatula without disturbing the amorphous layer, washed with ethyl acetate, and air-dried to give the ethyl acetate solvate of POCs.

### Preparation of Na_2_V_6_O_16_·1.5H_2_O (NVO) Cathodes

The cathode material NVO was prepared following established literature [48, 49]. 3 g of commercial V_2_O_5_ powder was added to 50 mL of 2M NaCl aqueous solution and stirred for 96 h at 35 °C. Subsequently, an orange-red gel formed, the suspension was washed several times with deionized water. Finally, the black-red product was obtained by freeze-drying. The NVO cathode was prepared using the slurry bonding method. NVO powder, Super P, and polyvinylidene fluoride (PVDF) were mixed with an appropriate amount of N-methyl-2-pyrrolidone (NMP) at a mass ratio of 7:2:1 and thoroughly ground. The mixture was then coated onto stainless steel (SS) as a slurry. The SS coated with the slurry was dried under vacuum at 80 °C for 12 h.

### Electrochemical Measurements

The electrochemical tests were conducted using 2032-type coin half cells, and all measurements were performed using a NEWARE Battery Test System (CT-40080-5V 100 mA-124, Shenzhen, China) on a laboratory bench at an environmental temperature of approximately 25 °C. To investigate the Coulombic efficiency (CE) of Zn plating/stripping, Ti/Cu foil was used as the working electrode, while Zn with different coating layers served as the counter electrode. During the Zn stripping process, the cutoff potential is set at 5 V. Zn deposition was carried out using either Zn foil as the working electrode, and Zn foil or Cu foil as the counter/reference electrode. For the full-cell tests, the Zn foil anode was assembled with a NVO cathode. For the electrochemical experiments described above, a 2M ZnSO_4_ + 0.2M NaSO_4_ or ZS-POC + 0.2M NaSO_4_ aqueous solution was used as the electrolyte. Electrochemical impedance spectroscopy (EIS), Tafel analysis, chronoamperometry (CA), linear sweep voltammetry (LSV), and cyclic voltammetry (CV) tests were recorded on an electrochemical workstation (CHI 760E, China). Tafel plots were obtained from Zn-Zn symmetric cells using the ZS and ZS-POC electrolyte. EIS measurements were conducted over a frequency range of 0.01–10^6^ Hz. Chronoamperograms (CA) of the Zn–Zn symmetric cells were recorded at a scan rate of 10 mV s^−1^ and under a − 150 mV overpotential, respectively.

## Results and Discussion

### LSI Design Methodology and Battery Performance Comparison

**Fig. 1:**
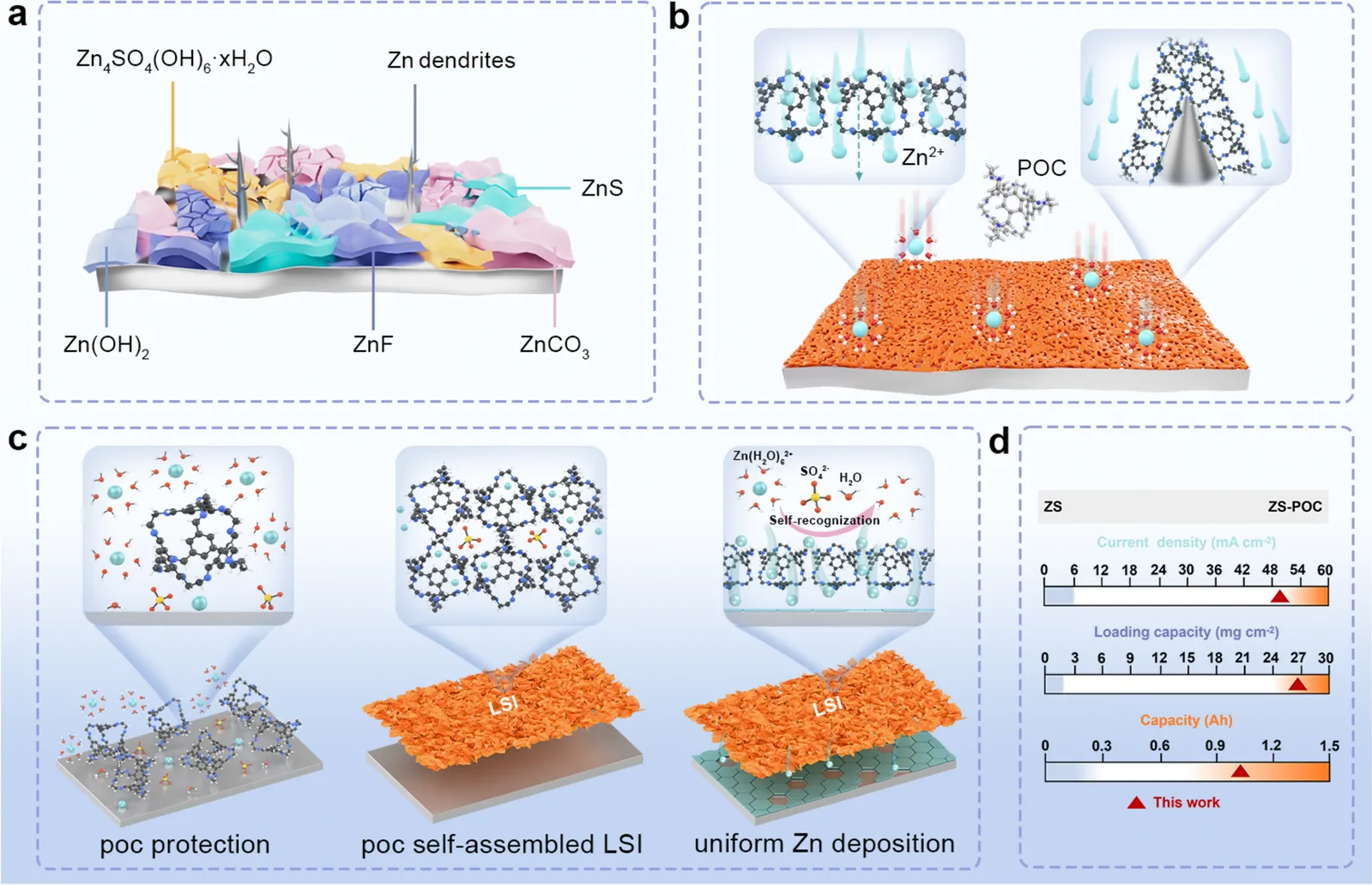
**a** Expansion, cracking and detachment issues of previously reported SEI. **b** POC-derived LSI characterized by loose porous structure, plenty of selective Zn^2+^-conducting channels and dynamic self-healing capability. **c** Assembly process for in situ construction of LSI and interface regulation mechanism for suppressing side reactions and dendrite growth. **d** Outstanding battery performance enabled by LSI

In standard electrolyte (2.0 mol L^−1^ ZnSO_4_, denoted as ZS), abundant reactive water and slow desolvation kinetics at the interface result in the severe HER, corrosion, dendrite growth [50]. As Zn^2+^ stripping/deposition proceeds, dendrite growth becomes more intense, piercing the separator and leading to battery failure (Fig. S1). In situ SEI can effectively mitigate the issues, but most of previously reported SEI relies on the constant consumption of electrolyte additive and is usually thick and fragile with the relatively low ionic conductivity and poor mechanical robustness. These SEIs are prone to expansion, cracking and detachment under high current and high depth of discharge, which subsequently induces dendrite growth and interface deterioration, thus limiting the battery performance for practical application (Fig. 1a). In this contribution, we introduce POC as a functional additive into ZS electrolyte to in situ construct a self-adaptive LSI at the Zn anode, where the LSI is featured with loose porous structure, plenty of selective Zn^2+^-conducting channels and dynamic self-repairing capability (Fig. 1b). When the dendrites form on the Zn anode, POC molecules within electrolyte are preferentially absorbed on these dendrites to induce dynamic interface reconstruction, which can successfully repair the dendritic Zn anode by guiding uniform Zn^2+^ deposition toward surrounding regions.

More specifically, POC additives migrate from the bulk electrolyte to the interface and accumulate on the Zn anode due to preferential adsorption, which subsequently self-assemble into LSI under fine-balance between POC-POC hydrogen-bond interactions and POC–cation/anion coordination interactions (Fig. 1c). The designed LSI confines the interfacial water activity, repels SO_4_^2−^ and selectively transport Zn^2+^, which suppresses side reactions and induces the uniform Zn deposition under harsh conditions (Fig. 1c). The designed LSI achieves breakthrough from laboratory button batteries to practical Ah-level pouch batteries and affords outstanding battery performance (Fig. 1d), where AZMBs can stably work at high current density of 50 mA cm^−2^ and high area capacity of 7.25 mAh cm^−2^ (cathode mass loading of 26.77 mg cm^−2^) and deliver the discharge capacity of over 1 Ah, thus surpassing most reported references (Table S1).

Cage1, a tetrahedral cage, is chosen as a typical representative of POC, which can be easily synthesized via [4 + 6] cycloimination reaction of 1,3,5-triformylbenzene (TFB) and ethylenediamine (EDA) (Figs. S2 and S3). The synthesis is carried out at room temperature and no additional template or catalyst is required. The morphology of synthesized POC is observed by scanning electron microscopy (SEM), revealing rod-shaped crystalline branches (Fig. S4). Energy-dispersive X-ray spectroscopy (EDS) mapping demonstrates the uniform distribution of C, N and O elements of POC (Fig. S5). Moreover, the crystal structure and chemistry are further characterized by X-ray diffraction (XRD) and ATR-FTIR spectroscopy. The obvious diffraction peaks indicate the high crystallinity of POC (Fig. S6), while the presence of C=N stretching vibrations in ATR-FTIR spectra verify the successful fabrication of POC (Fig. S7).

### Morphology and Chemistry of LSI

**Fig. 2:**
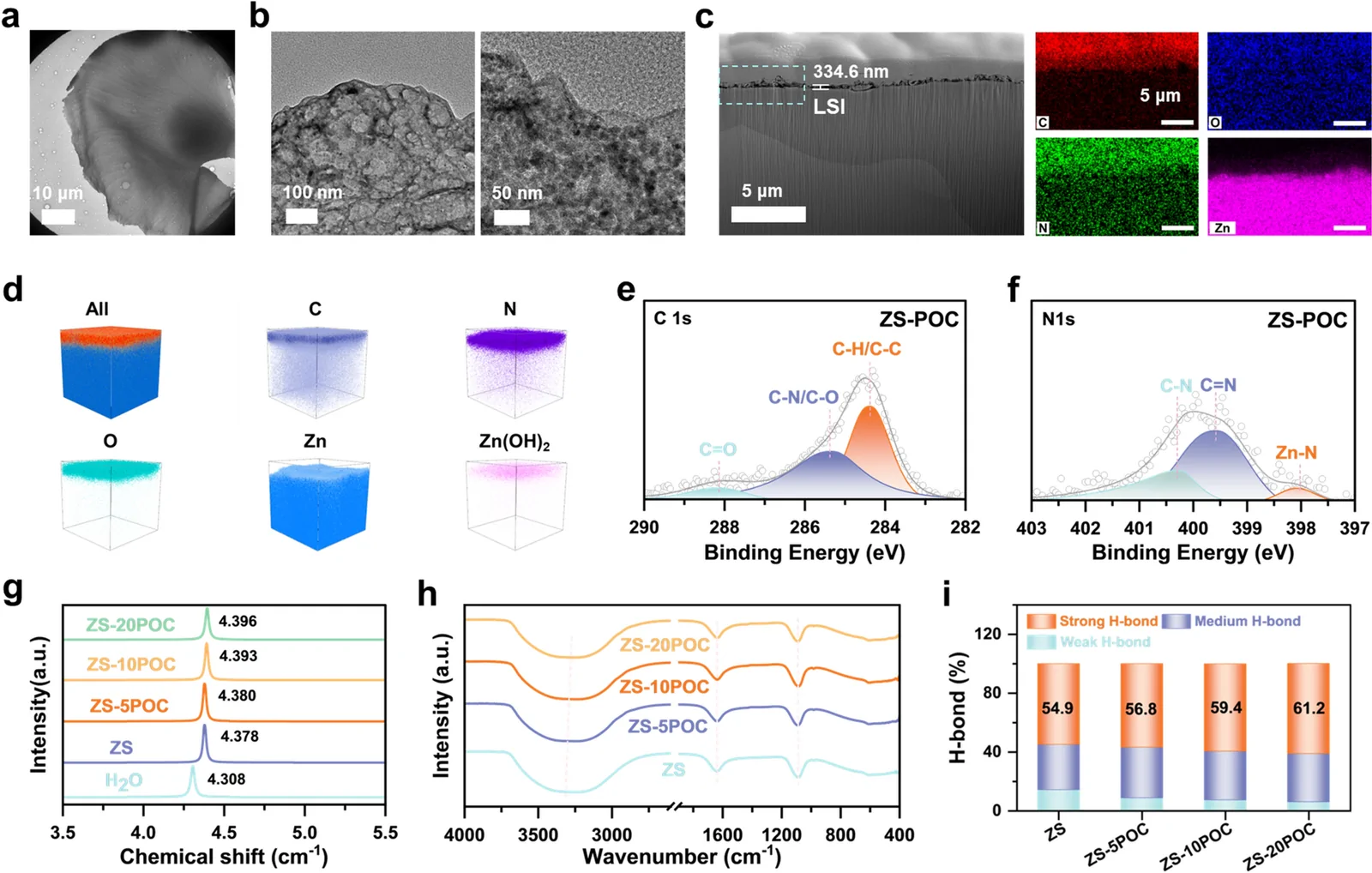
**a** TEM image of self-assembled POC film in pure aqueous solution. **b** TEM images of LSI formed in ZS-POC electrolytes. **c** FIB-SEM and EDS mapping images of self-assembled POC film and LSI. **d** TOF–SIMS 3D mapping of LSI on Zn anode after 7-day immersion in ZS-POC electrolyte and the resultant spatial distribution of C, N, O, Zn of and Zn (OH). **e** C 1*s* and **f** N 1*s* XPS spectra of LSI on Zn anode after 7-day immersion in ZS-POC electrolyte. **g** ^1^H NMR spectra of pure H_2_O, ZS and ZS-POC electrolytes. **h** FTIR spectrum of ZS and ZS-POC electrolytes. **i** Hydrogen-bond network in ZS and ZS-POC electrolytes revealed by Raman spectra

A series of ZS-POC electrolytes were prepared by dissolving POC into the ZS solution to form electrolytes with different concentrations of 5, 10 and 20 mg mL^−1^, which are denoted as ZS-5POC, ZS-10POC and ZS-20POC, respectively (Fig. S8). At concentration of 20 mg mL^−1^, electrolyte changes from transparent to white, which suggested the occurrence of POC aggregation [51]. Tyndall effect further proves that the ZS-POC electrolytes have good dispersibility and form the colloidal electrolytes, which create the prerequisite for LSI formation via POC self-assembly at the electrode interface. Moreover, ZS-POC electrolytes exhibit high ionic conductivity even at POC concentration of 20 mg mL^−1^, and the slight decrease in ion conductivity indicates the addition of POC hardly sacrifices ion conduction dynamics (Fig. S9). The morphology and chemistry of the self-assembled LSI are studied by Transmission electron microscopy (TEM), SEM, focused ion beam-scanning electron microscope (FIB-SEM), time-of-flight secondary ion mass spectrometry (TOF–SIMS) and X-ray photoelectron spectroscopy (XPS). As highlighted by TEM images, POC can self-assemble into a dense film on the interface in pure aqueous solution due to the POC-POC intermolecular interactions (Fig. 2a). In ZS electrolyte, a loose and porous layer on the interface is formed, which suggest the coordination interactions between Zn^2+^/SO_4_^2−^ and POC can precisely tailor the self-assembly morphology of POC (Fig. 2b). The LSI is further corroborated by FIB-SEM. The results showed that the LSI is an ultrathin structure with a thickness of approximately 334.6 nm, and its surface exhibits a large number of loose pores and clear grain boundary features. This porous ultrathin structure provides abundant and continuous channels for the transport of Zn^2+^, which is beneficial for ion diffusion and conduction (Figs. 2c and S10). The EDS mapping image shows a uniform distribution of C, N, Zn, S and O elements, indicating the integrity and homogeneity of LSI on the Zn anode (Figs. S11-S12). The chemical composition of LSI is further studied by TOF–SIMS and XPS. TOF–SIMS 3D mapping images of Zn anode after 7-day immersion in ZS-POC electrolyte confirm the formation of a well-defined LSI constructed by POC, which is composed of C, N, and O elements with uniform spatial distribution (Fig. 2d). More importantly, LSI can effectively suppress side reactions and the formation of by-products, as recognized by the extremely low content of Zn (OH)_2_. As further evidenced by XPS spectra, C 1s spectrum exhibits the characteristic peaks corresponding to C–C/C–H and C=N/C–N bonds of POC, validating the self-assembly of POC for constructing LSI (Fig. 2e). In addition, the N 1s spectrum confirms the formation of Zn–N bonds, indicating coordination between Zn^2+^ and nitrogen sites of POC for forming LSI (Fig. 2f).

^1^H nuclear magnetic resonance (^1^H NMR), ATR-FTIR spectroscopy, and Raman spectroscopy are performed to investigate the structure and intermolecular interactions of ZS-POC electrolytes. The ^1^H NMR spectra of ZS-POC electrolytes reveal the chemical shift of H_2_O moves to the low-field, indicating that the hydrogen-bond network between water molecules is disrupted due to the POC-H_2_O interactions (Figs. 2g and S13). The newly formed interactions are further supported by the slight redshift of H_2_O stretching vibration in ATR-FTIR spectra. Importantly, ATR-FTIR spectra indicate no change of the SO_4_^2−^ characteristic peaks (Figs. 2h and S14), suggesting that the Zn^2+^ solvation structure is not modified by POC addition. Raman spectroscopy provides additional insight into the hydrogen-bond network (Fig. S15). The O–H stretching vibration peak (2600–4000 cm^−1^) can be deconvoluted into three characteristic peaks (Figs. S16 and S17), corresponding to weak, medium, and strong hydrogen-bond networks, respectively. The deconvoluted peak area analysis reveals that POC increases the proportion of strong hydrogen bonds (Fig. 2i), which is conducive to confine water activity for suppressing side reactions [52]. Consistent with the ATR-FTIR results, the SO_4_^2−^ Raman peak remains fixed at 987 cm^−1^, further confirming the unaltered Zn^2+^ solvation shell. Therefore, battery performance enhancement is mainly attributed to the LSI.

### Formation Mechanism of LSI

**Fig. 3:**
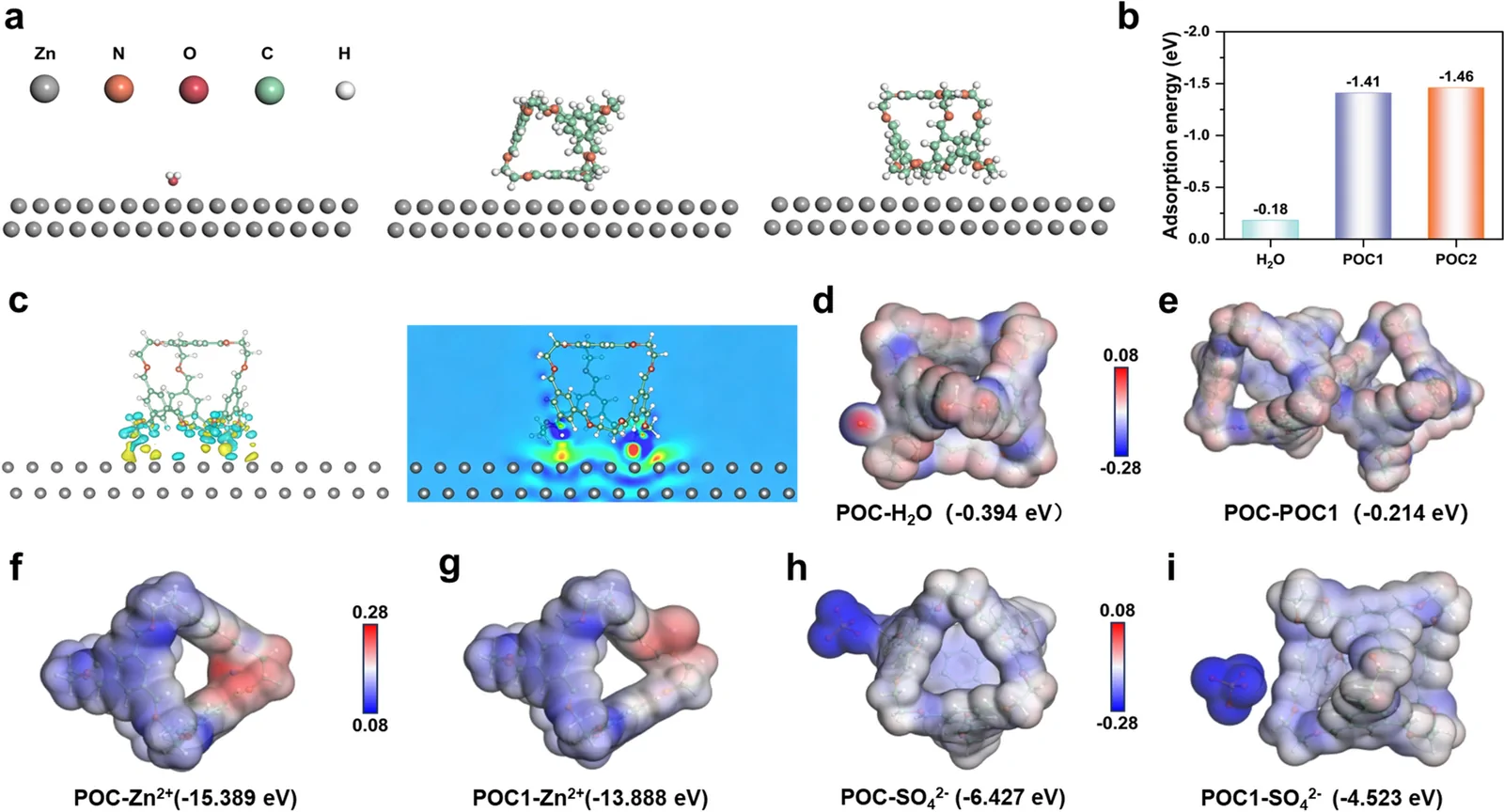
**a** Optimized adsorption modes of H_2_O and POC (two different molecular arrangements) on Zn (002) crystal plane. Color code: purple O, white H, mint green C, orange red N and gray Zn anode. **b** Adsorption energies of H_2_O-Zn anode and POC-Zn anode. **c** The charge density difference distribution of POC on the Zn anode and the corresponding sliced 2D contour map. **d, e** ESP potential distribution and binding energy of POC-H_2_O and POC-POC. **f, g** ESP potential distribution and binding energy of POC-Zn^2+^ with different interaction sites. **h, i** ESP potential distribution and binding energy of POC-SO_4_^2−^ with different interaction sites

The formation mechanism of LSI is investigated by a series of first-principles density functional theory (DFT) calculations. The calculated adsorption energy of the POC (− 1.47 and − 1.50 eV) toward (002) crystal plane of Zn anode is notably higher than the value obtained for H_2_O (− 0.20 eV), indicating the preferential adsorption of POC molecules on the Zn anode (Figs. 3a, b and S18). Moreover, POC delivers the isotropic adsorption behavior, which arises from the three-dimensional cage-like structure of POC. The charge density difference analysis reveals the significant electron transference from Zn anode to POC (Figs. 3c and S19). Notably, the lowest unoccupied molecular orbital (LUMO) and the highest occupied molecular orbital (HOMO) for H_2_O and POC are also compared. The narrower LUMO–HOMO gap of POC indicates its high electrochemical reactivity for interfacial regulation (Fig. S20) [19, 53]. Specifically, the higher HOMO energy level of POC (− 0.21 vs. − 0.25 eV of H_2_O) suggests that POC favorably captures Zn^2+^ to facilitate interfacial kinetics, and meanwhile, the lower LUMO energy level of POC (− 0.09 vs. 0.04 eV of H_2_O) indicates the POC easily accepts electrons from the Zn metal anode to achieve strong adsorption. Therefore, LSI formation is attributed to the isotropic preferential adsorption of POC toward Zn anode, which promotes the diffusion of POC from the bulk electrolyte to the interface and subsequent self-assembly.

DFT calculations are performed to reveal the electrostatic potential (ESP) and binding energies of POC-H_2_O, POC-POC, POC-Zn^2+^ and POC-SO_4_^2−^. The ESP map indicates POC molecules possess the abundant positively and negative charged sites, which endows them as ideal platform for constructing robust LSI via multi-site collaboration. As shown in Fig. 3d–i, the lower binding energy of POC-POC compared with that of POC-H_2_O suggests hydrogen-bonding interactions of POC-POC are too weak to afford the LSI formation. Notably, POC-Zn^2+^ and POC-SO_4_^2−^ deliver much higher binding energies than that of POC-H_2_O, which indicates POC–anion/cation interactions play a crucial role for self-assembly of POC. Importantly, SO_4_^2−^ and Zn^2+^ can bind with POC via different sites, which acts as cross-linker for LSI construction. Combined with above morphology characterization of LSI, it is anticipated that the delicate balance between hydrogen-bonding interactions of POC-POC and coordination interactions of POC–anion/cation endows LSI with porous loose structure. Moreover, the binding energy of other electrolyte components is also explored (Figs. S21 and S22). The higher binding energy of POC-H_2_O and SO_4_^2−^-H_2_O in relative to H_2_O-H_2_O means the LSI can feasibly confine H_2_O activity via the hydrogen-bond reconstruction to suppress HER. And the stronger interaction between POC molecules and Zn^2+^ suggests the polar functional groups of POC prefer to recognize and coordinate strongly with Zn^2+^ ions, thus facilitating Zn^2+^ conduction and uniform deposition.

### Regulation of Zn^2+^ Interfacial Diffusion and Desolvation Kinetics

**Fig. 4:**
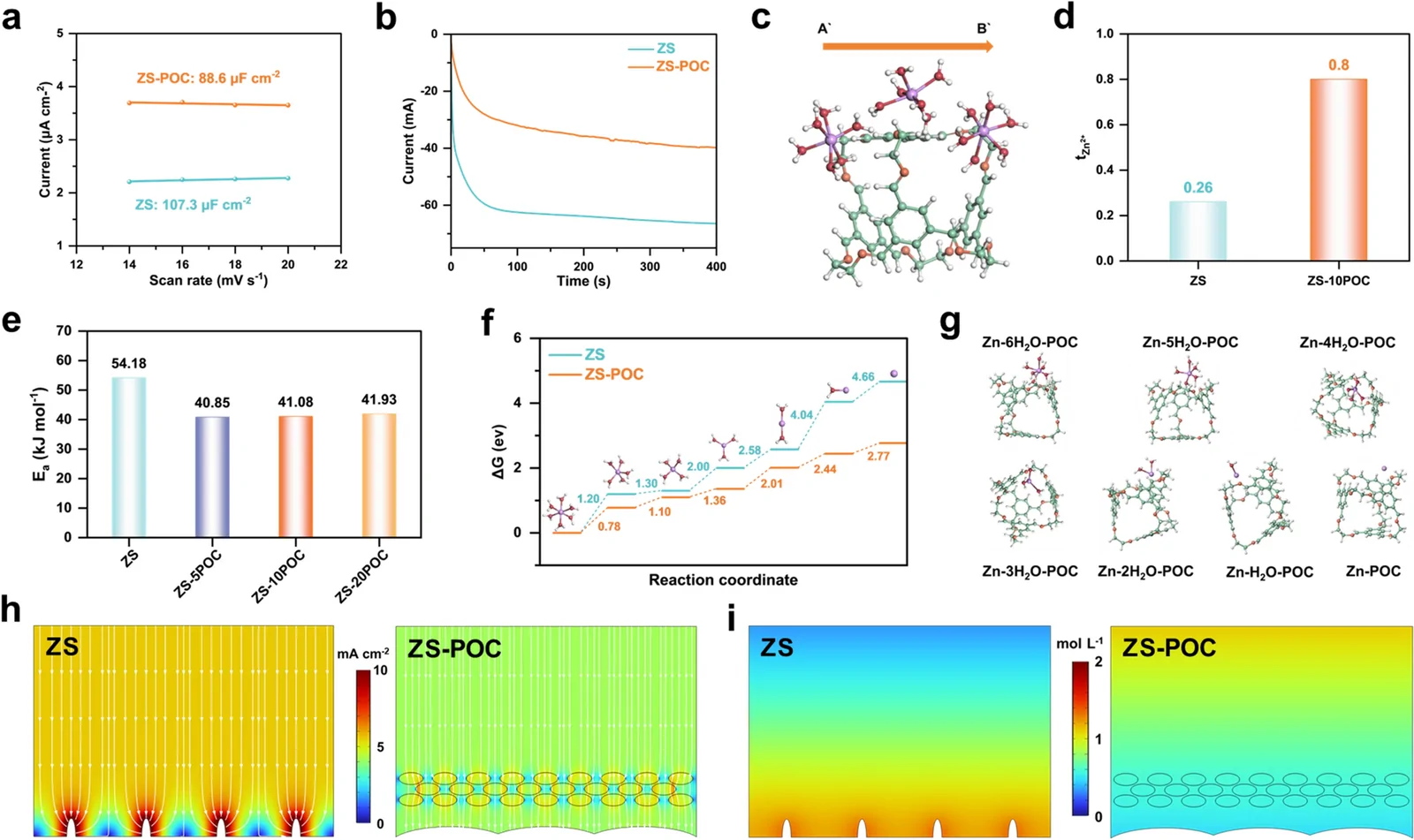
**a** Differential capacitance curves of ZS and ZS-POC electrolytes. **b** Chronoamperometry (CA) curves of ZS and ZS-POC electrolytes. **c** Zn^2+^ migration pathways in lattice structures of POC. **d** Zn^2+^ transference numbers in ZS and ZS-POC electrolytes. **e** Activation energies in ZS and ZS-POC electrolytes. **f** Desolvation energy barrier for hydrated Zn^2+^ in ZS and ZS-POC electrolytes. **g** Desolvation models for Zn^2+^ with different hydration degrees. **h** Simulated electric field distributions and **i** Zn^2+^ concentration field distributions during the Zn plating process on the Zn anode in ZS and ZS-POC electrolytes

The designed LSI effectively regulates the electric double layer (EDL) structure, Zn^2+^ diffusion flux and desolvation kinetics. As highlighted in Figs. 4a and S23, both the EDL capacitance and differential capacitance are significantly reduced for ZS-POC electrolytes, which indicates the LSI tailors the EDL structure and POC molecules enter the EDL. These POC molecules within the EDL can readily repair cracks and defects of LSI, thus enabling the self-repairing capacity. Additionally, LSI manipulates Zn^2+^ diffusion flux through the selective Zn^2+^-conducting channels (Fig. 4b), which exhibits a three-dimensional dominance diffusion behavior in the ZS-POC electrolyte. Cyclic voltammetry (CV) curves further reveal that LSI increases the nucleation overpotential, inducing dense and uniform Zn nucleation (Fig. S24). Therefore, the favorable Zn^2+^ flux and nucleation are conducive to dendrite-free Zn deposition.

DFT calculations demonstrate that polar functional group sites of the POC promote Zn^2+^ diffusion (Fig. 4c), where the diffusion energy barrier of Zn^2+^ migration along POC molecular chains is approximately 0.62 eV, suggesting efficient ion transport (Fig. S25). Consistently, Zn^2+^ transference number increases dramatically from 0.26 to 0.8 in the presence of LSI (Figs. 4d and S26), which further confirms the selective Zn^2+^-conducting channels favor Zn^2+^ transfer but immobilize SO_4_^2−^ anion. Moreover, the interfacial Zn^2+^ transfer through LSI and desolvation kinetics are further analyzed via calculating activation energy (*E*_a_) using the Arrhenius equation. Give the high electrical conductivity of Zn anode and high ion conductivity of ZS-POC electrolyte, the contributions from the Zn^2+^ reduction and bulk diffusion barrier are negligible. The *E*_a_ of ZS-POC electrolyte is 41.08 kJ mol^−1^, substantially lower than that of ZS electrolyte (54.18 kJ mol^−1^), confirming the accelerated interfacial kinetics with faster Zn^2+^ transfer and desolvation (Figs. 4e and S27) [54]. The desolvation energy barrier of solvated Zn^2+^ is also examined via DFT by stepwise removal of water molecules from the typical solvation structure of [Zn (H_2_O)_6_]^2+^. As shown in Figs. 4f, g and S28–S29, the desolvation energy of Zn^2+^ is significantly reduced in the ZS-POC electrolyte, which further confirms the POC within LSI contributes to the improvement of desolvation process, thus alleviating concentration polarization and avoiding Zn^2+^ depletion.

COMSOL multiphysics field simulation is conducted to analyze the concentration and electric field distributions [55]. Zn anode with ZS electrolyte shows a distinct non-uniform electric field distribution with pronounced tip effects (Fig. 4h), whereas ZS-POC electrolyte exhibits a uniform electric field distribution due to the existence of LSI. The Zn^2+^ concentration field in ZS electrolyte exhibits that the protrusions on the Zn anode heighten Zn^2+^ local accumulation and induce a non-uniform Zn^2+^ concentration gradient, which results in the formation of sharp dendrites. In comparison, Zn anode with ZS-POC electrolyte demonstrates a homogeneous concentration distribution with strengthened Zn^2+^ ion flux due to the abundant Zn^2+^-conducting channels within LSI (Fig. 4i), ensuring the rapid and even supplement of Zn^2+^ ion during Zn^2+^ deposition. Therefore, the predominant Zn^2^⁺ transport, accelerated desolvation, and the uniform electric field distribution enabled by the LSI facilely support high reversibility of Zn anode under harsh conditions.

### Dendrite-Free Zn Anode Enabled by LSI

**Fig. 5:**
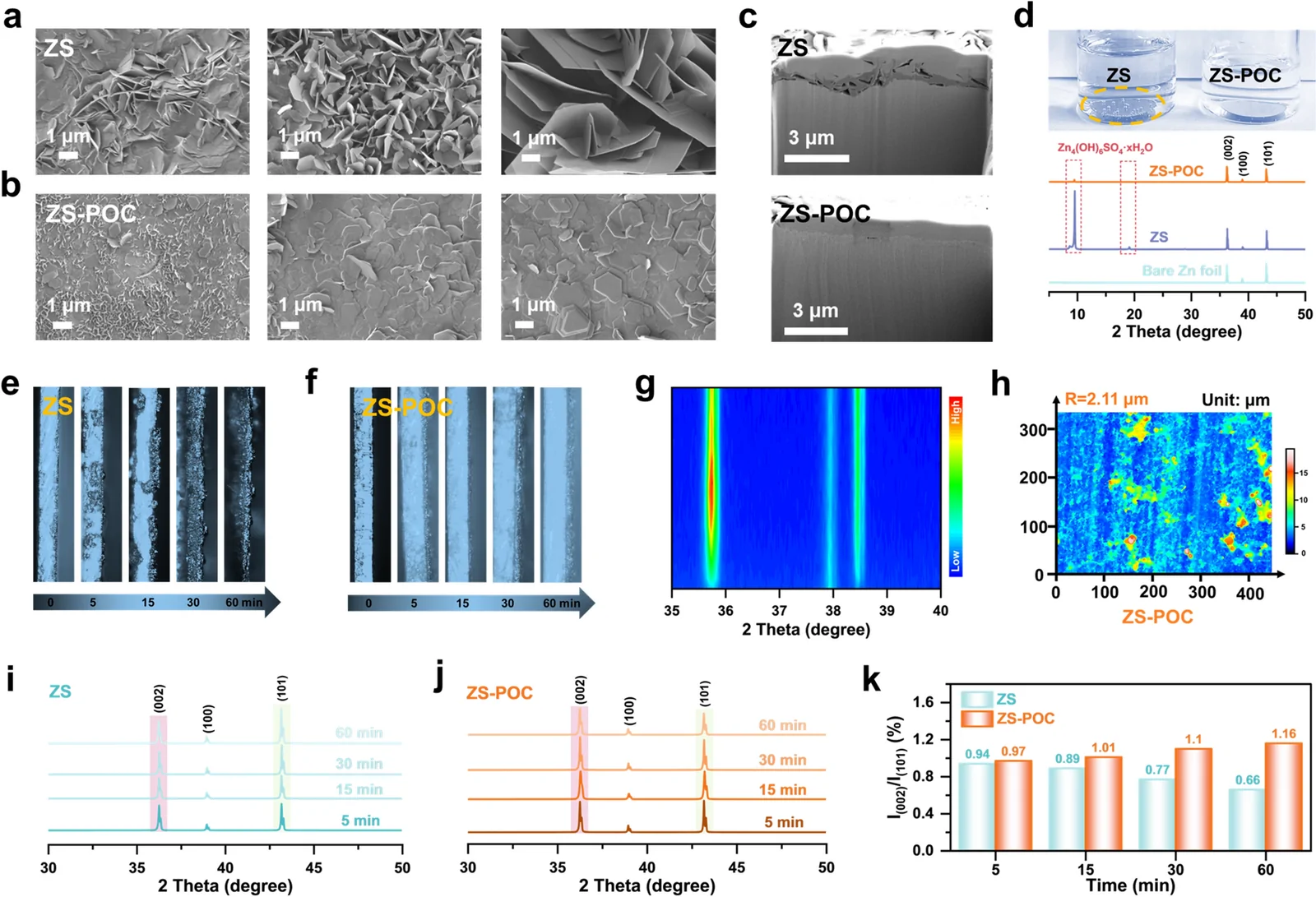
**a, b** SEM of Zn anode in the ZS and ZS-POC electrolytes after the specified plating/stripping cycles. **c** FIB-SEM images of Zn anodes after 30 cycles in ZS and ZS-POC electrolytes. **d** XRD patterns of Zn anode soaked in ZS and ZS-POC electrolytes after 7 days, and the illustration is an optical image of immersion experiments for Zn anode after 7 days. **e, f** In situ optical microscope images of Zn anode during Zn^2+^ plating in the ZS and ZS-POC electrolytes. **g** In situ XRD patterns of Zn anode during Zn^2+^ plating in ZS-POC electrolyte. **h** CLSM 2D images of Zn anode in ZS-POC electrolyte after 60 min plating. **i-k** XRD patterns of Zn anode in ZS and ZS-POC electrolytes and the corresponding Zn(002)/(101) crystal plane ratio with different plating times

The morphology and crystallographic evolution of the Zn anode with LSI are investigated by the in situ and ex situ characterizations. The morphology of Zn anode after 10, 30 and 100 cycles at 0.5 mA cm^−2^ and 0.5 mAh cm^−2^ is observed by SEM (Figs. 5a, b and S30-S31) [56]. In ZS electrolyte, Zn dendrites grow irregularly and are randomly distributed throughout the anode surface. As the cycle proceeds, dendrites growth worsens and their size sharply increases, ultimately penetrating the separator and causing battery failure. In contrast, Zn anode in ZS-POC electrolyte remains flat and compact throughout 100 cycles, demonstrating the effective dendrite suppression by the Zn^2+^-conducting LSI. Furthermore, FIB-SEM is performed to observe the cross-sectional microstructure of Zn anode after 30 cycles (Figs. 5c and S32). In ZS electrolyte, a rough deposition layer with irregular internal voids is formed on Zn anode (Figs. 5c and S32a), while a dense deposition layer yields in ZS-POC electrolytes (Figs. 5c and S32b) [57]. Moreover, LSI remarkably suppress self-corrosion and HER during the static period, as evidenced by the SEM images and XRD patterns of Zn anode soaked in ZS and ZS-POC electrolytes for 7 and 14 days (Figs. 5d and S33–S35). As highlighted by the immersion experiments after 7 days, Zn anode in ZS electrolyte shows many bubbles, which suggest the severe HER. In contrast, no bubbles are generated in the ZS-POC electrolyte due to the in situ formation of LSI. SEM is performed to observe the morphology of Zn anode after immersion experiment, and Zn anode in ZS electrolyte is covered with plate-like by-products after 7 days, which are identified as Zn_4_(OH)_6_SO_4_·xH_2_O by-products based on XRD analysis. Their thickness and size become larger after 14 days, which suggest the sustained self-corrosion. In ZS-POC electrolyte, Zn anode remains the smooth surface and free of detectable by-products even after 14 days.

In situ optical microscopy (OM) and in situ XRD are employed to monitor Zn^2+^ deposition behavior. OM observations reveal Zn anode in ZS electrolyte forms many irregular protrusions that gradually evolve into large dendrites as plating proceeds (Fig. 5e). In contrast, ZS-POC electrolyte facilitates the uniform and dense Zn deposition benefiting from LSI (Fig. 5f). As revealed by in situ XRD, Zn anode in ZS-POC electrolyte exhibits a significantly enhanced (002) diffraction peak with the increase in plating time, indicating that the LSI promotes the (002) crystal plane orientation (Fig. 5g) [58]. Moreover, surface topology of the deposited Zn is analyzed by 2D confocal laser scanning microscope (CLSM) images (Figs. 5h and S36) [59]. Zn surface in ZS electrolyte is extremely rough (*R* = 53.86 μm), where the rough deposition structure increases the electroactive area, thus accelerating HER and the formation of by-products. Encouragingly, Zn surface in ZS-POC electrolyte is significantly smoother (*R* = 2.11 μm). Furthermore, ex situ XRD is conducted to further investigate the crystal evolution of deposited Zn at high current density of 5 mA cm^−2^ (Fig. 5i–k). The ratio intensity of I_002_: I_101_ rises from 0.97 to 1.16 in ZS-POC electrolytes as the plating time increases from 5 to 60 min, while the opposite trend occurs in the ZS electrolyte, which strongly confirm that LSI can promote the preferential deposition along the (002) plane, thus contributing to the inhibition of HER and dendrite growth [60, 61].

The inhibition of HER and by-product formation enabled by LSI are confirmed by the combination of experiments and theoretical calculations. The hydrophobicity of LSI on Zn anode is evaluated by contact angle tests. The contact angle rises from 87.1° to 108.7° with the increase in POC content, which indicates the enhanced hydrophobicity (Figs. S37 and S38). Meanwhile, the pH of ZS-POC electrolyte is slightly elevated relative to the ZS electrolyte (Fig. S39). DFT calculations reveal that POC effectively confines the thermodynamic activity of solvated H_2_O, which boosts the deprotonation energy barrier of solvated H_2_O (Fig. S40) [62]. Therefore, the enhanced hydrophobicity, the improved pH and the confined H_2_O of LSI collectively inhibit HER and the formation of by-products. Electrochemical tests further validate the suppression of HER and by-product (Fig. S41). As indicated by Tafel curves, ZS-POC electrolyte has shifted the corrosion potential of the Zn anode from − 28.65 to − 23.63 mV, indicating improved corrosion resistance. Linear polarization (LSV) curves suggest that the presence of LSI can effectively inhibit the HER (Fig. S42), as verified by the increased HER overpotential (Fig. S43).

### Suppression of HER and By-Product Formation via LSI

**Fig. 6:**
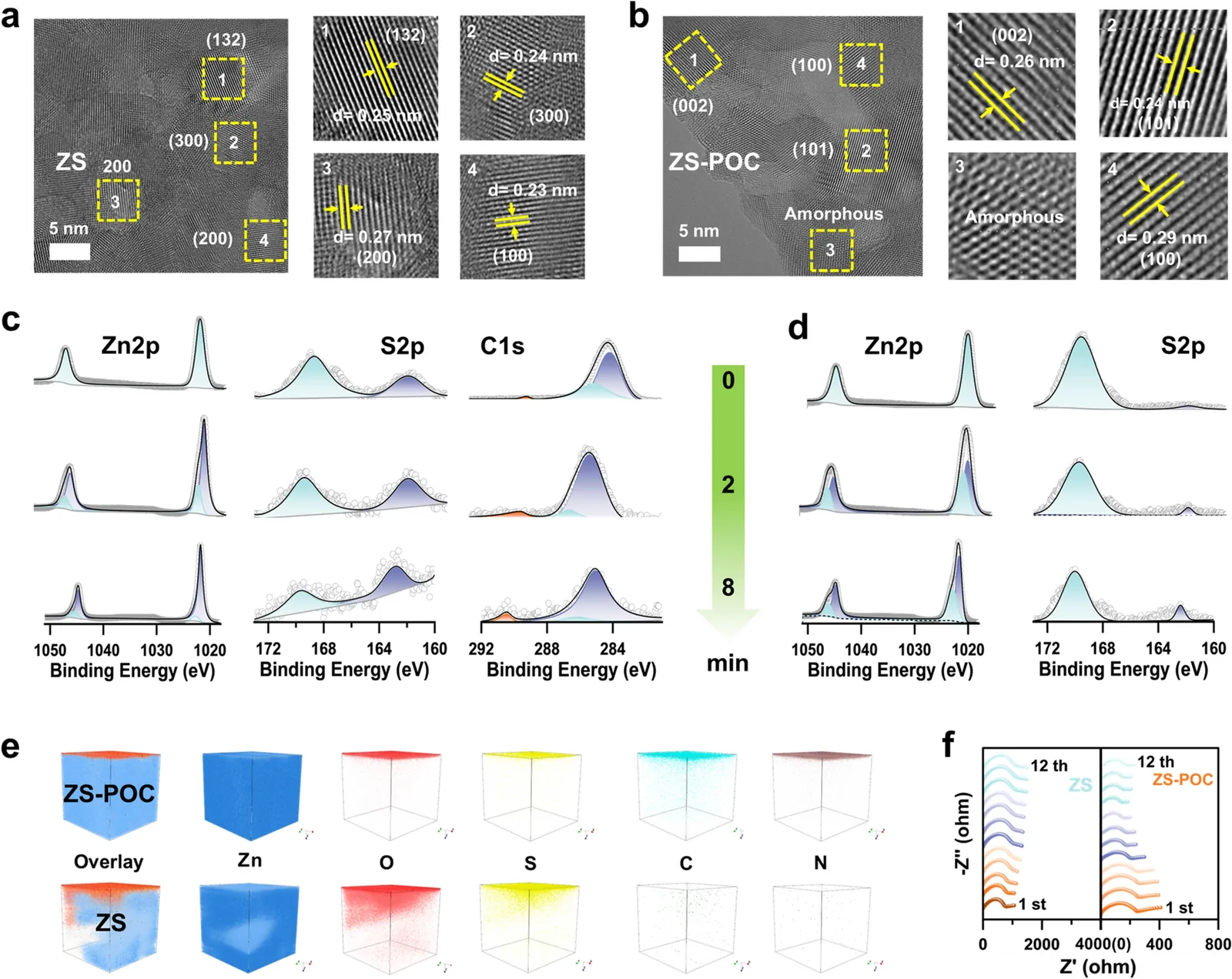
**a, b** HRTEM images of Zn anode cycled in ZS and ZS-POC electrolytes. **c** Zn 2*p*, S 2*p* and C 1*s* XPS spectra with depth profiles of Zn anodes cycled in ZS-POC electrolyte after Ar⁺ sputtering for 0, 2 and 8 min. **d** Zn 2*p* and S 2*p* XPS spectra with depth profiles of Zn anode cycled in ZS electrolyte after Ar^+^ sputtering for 0, 2 and 8 min. **e** TOF–SIMS 3D mapping of Zn anode after 30 cycles in ZS-POC and ZS electrolytes, showing the spatial distribution of Zn, O, S, C and N. **f** In situ EIS curves of Zn//Zn symmetric cells using ZS and ZS-POC electrolytes

The interfacial chemistry of Zn anode cycled in ZS and ZS-POC electrolytes are further investigated by TEM, XPS and TOF–SIMS. As seen from TEM and HRTEM images, Zn anode in ZS electrolyte shows the typical lattice fringes of Zn (OH)_2_ and ZnSO_4_, which resulting from interfacial by-product (Fig. 6a). On the contrary, Zn anode cycled in ZS-POC electrolyte exhibits the amorphous regions of organic components and inorganic crystalline regions of Zn metal with (002), (100), and (101) crystal planes, where further confirms the in situ formation of LSI and the highly reversible Zn deposition (Fig. 6b). XPS survey spectra demonstrate a higher Zn and a lower O elemental content for the Zn anode in the ZS-POC electrolyte relative to the ZS electrolyte (Fig. S44). The high-resolution Zn 2*p* XPS spectra of Zn anodes after 30 cycles clearly discern the Zn^2+^ and metallic Zn (Fig. 6c, d), and Zn^2+^ is the dominant species on the Zn anode surface for both ZS and ZS-POC electrolytes. However, as sputtering time increases, the Zn^2+^ signal sharply decreases and metallic Zn become the dominant component in ZS-POC electrolyte. By contrast, Zn^2+^ maintains a relatively high proportion in ZS electrolyte even after 8 min sputtering, which originates from the continuous side reactions from the surface to inner layer of the Zn anode. The S 2*p* spectra of Zn anode in ZS-POC electrolyte show the much lower SO_4_^2−^ content compared with that of ZS electrolyte, which aligns with Zn 2*p* XPS spectra [63]. A small amount of ZnS probably root in the decomposition of SO_4_^2−^ induced by high-energy Ar^+^ sputtering. Moreover, Zn anode in ZS-POC electrolyte is detected with C 1*s* peaks corresponding to C–N/C=N and C–H/C–C species, and these peaks are primarily attributed to the residual POC on Zn anode after washing treatment (Fig. 6c).

The elemental spatial distribution of Zn anode interface is further analyzed by TOF–SIMS (Fig. 6e). Zn anode with ZS-POC electrolyte exhibits the uniform O, S, C and N distribution on Zn anode surface, and the dense Zn deposition under LSI. In contrast, the anode with ZS electrolyte exhibits severe heterogeneity, with intense and uneven signals for O, S, Zn (OH)₂ and SO_4_^2−^, indicative of substantial by-product formation (Fig. S45). The interface stability of Zn anode with LSI is observed via in situ EIS. Due to the absence of stable interface in ZS electrolyte, the resistance of Zn anode gradually decreases due to the repeated exposure of fresh Zn and dendritic growth (Fig. 6f). On the contrary, the charge-transfer resistance rises slightly during initial LSI formation, then decreases and stabilizes in subsequent cycles. Therefore, LSI effectively suppresses HER and by-product formation, thus enhancing the reversibility and cycling stability of the Zn anode.

### Superior Battery Performance

**Fig. 7:**
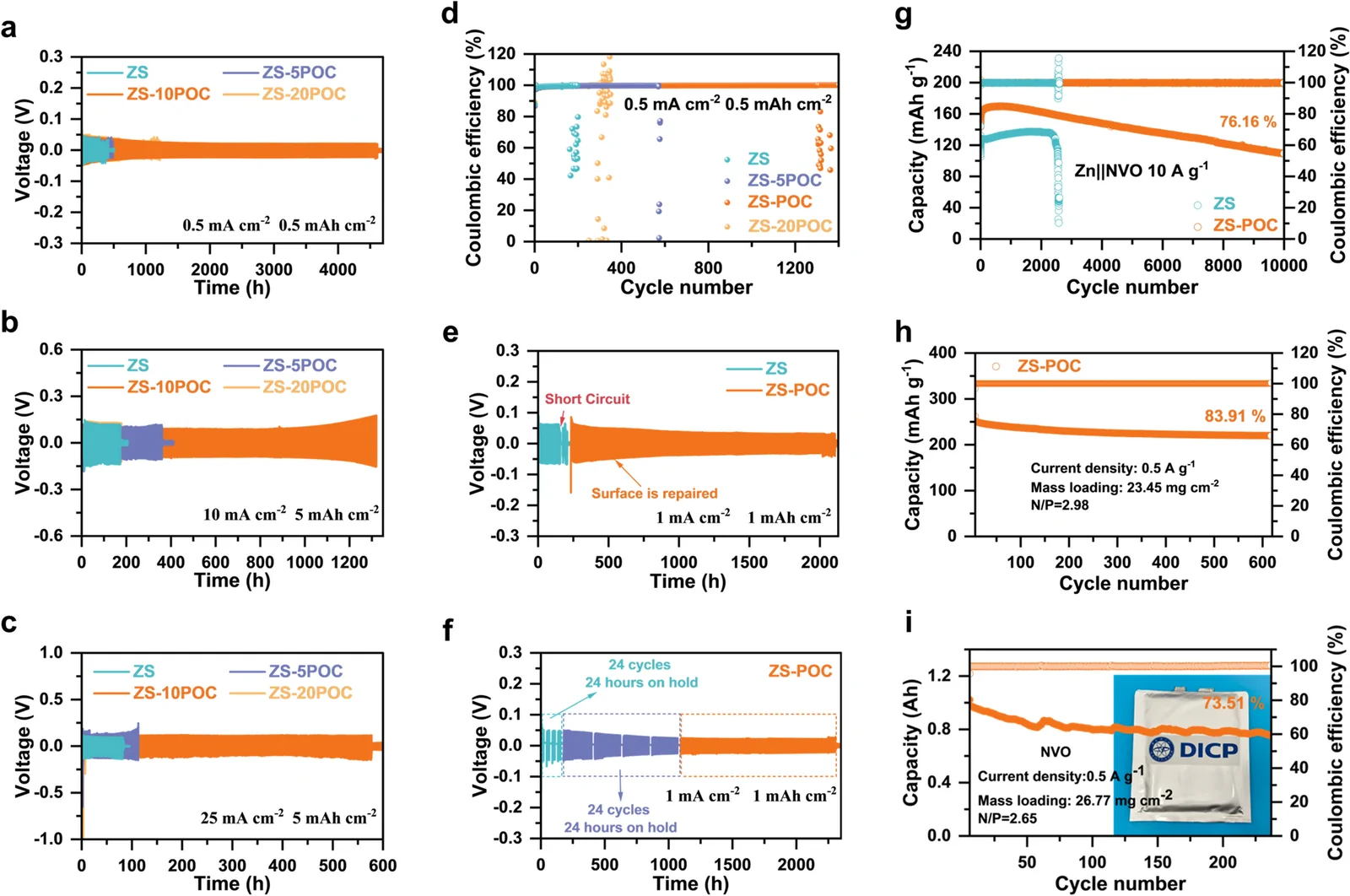
Galvanostatic plating/stripping of Zn//Zn symmetric cell with ZS and ZS-POC electrolytes at **a** 0.5 mA cm^−2^ and 0.5 mAh cm^−2^, **b** 10 mA cm^−2^ and 5 mAh cm^−2^, **c** 25 mA cm^−2^ and 5 mAh cm^−2^. **d** CE of Zn//Cu cell with ZS and ZS-POC electrolytes at 0.5 mA cm^−2^ and 0.5 mAh cm^−2^. **e** Cycling performance of Zn//Zn symmetric cell in ZS electrolyte and reconstituted in ZS-POC electrolyte after short circuit. **f** Cycling performance of Zn//Zn symmetric cell under repeated resting–cycling test. **g** Cycling perormance of Zn//NVO full cells with ZS and ZS-POC electrolyte at 10 A g^−1^. **h** Long-term cycling stability of Zn//NVO cell with mass loading of 23.45 mg cm^−2^ at a current density of 0.5 A g^−1^. **i** Cycling performance of Zn//NVO pouch cell assembled with ZS-POC electrolyte at 0.5 A g^−1^

The feasibility of LSI is confirmed by the superior battery performance. Zn//Zn symmetric cells with ZS-POC electrolytes exhibit better cycling life than that of ZS electrolyte (Fig. 7a–c). Optimization of the POC concentration identifies 10 mg mL^−1^ as optimal, balancing the formation of a uniform LSI and efficient Zn^2+^ transport. Lower concentration leads to incomplete interface formation, while higher concentration thickens the LSI and hinders ion conduction. With the optimized electrolyte, Zn//Zn cells achieve exceptional long-term stability at both low and high current densities, operating for over 4620 h at 0.5 mA cm^−2^ and 0.5 mAh cm^−2^, 1320 h at 10 mA cm^−2^ and 5 mAh cm^−2^ and 580h at 25 mA cm^−2^ and 5 mAh cm^−2^, respectively. Conversely, the cells with ZS electrolyte present poor cycling stability and suffer from short-circuiting. Notably, ZS-POC electrolyte enables the Zn//Zn cell to sustain excellent cycling stability even at an ultra-high current density of 50 mA cm^−2^ (Fig. S46), demonstrating the robustness of the LSI under harsh conditions. The rate performance is determined over a range of current densities of 0.5–10 mA cm^−2^ (Fig. S47). Zn//Zn cell with the ZS-POC electrolyte shows a more stable and lower voltage hysteresis compared to that with ZS electrolyte. Upon returning the current density to 1 mA cm^−2^, Zn//Zn cell with ZS-POC electrolyte resumes normal operation without degradation. Furthermore, using an ultrathin Zn anode (20 μm), the cell maintains a long cycle life of 370 h at an exceptionally high utilization rate (85.4%) (Fig. S48). As summarized in Table S1, the cycling life span and current density surpass most previously reported values, underscoring the practical promise of LSI.

CE is a critical metric for evaluating the commercial viability of electrolytes. Zn//Cu cells with ZS-POC electrolyte achieve an ultra-high average CE of 99.93% and stable operation for over 1200 h, whereas cell using ZS electrolyte displays the significant CE fluctuations and rapid failure within 200 cycles (Figs. 7d and S49). In addition, cycling stability of Zn//Zn cell fabricated by the pre-soaked Zn anode is also tested. Zn//Zn cell in ZS witnesses the abnormal voltage–time profiles and fast battery failure, while Zn//Zn cell with ZS-POC electrolyte maintains stable cycling, confirming the effectiveness of the in situ formed LSI in both static and cycling periods (Fig. S50). More importantly, ZS-POC electrolyte offers self-repairing capacity. A Zn anode is first cycled in the ZS electrolyte to induce dendrite formation (Fig. 7e) and then reassembled into a fresh Zn//Zn cell using ZS-POC electrolyte. After a brief polarization increase corresponding to rapid LSI reformation by POC, Zn//Zn cell quickly revives and cycles stably for over 2000h, demonstrating the good self-repairing capacity. Then, SEM characterization of the failed Zn anode in ZS electrolyte revealed a rough surface densely populated with zinc dendrites, whereas the anode retrieved from the ZS-POC electrolyte after 30 cycles exhibited a notably smooth and compact morphology, which directly corroborates the self-repairing capability of the POC-derived interface (Fig. S51). Moreover, ZS-POC electrolyte effectively suppresses self-corrosion at static period and extends the cycling life of battery under practical charge–discharge conditions, as evidenced by the prolonged cycling life of Zn//Zn cells subjected to repeated resting-cycling test (Fig. 7f).

In situ LSI remarkably improves the full-cell performance, where NaV_3_O_8_·1.5H_2_O (NVO) is selected as the cathode. The successful synthesis of NVO nanobelts is confirmed by SEM and EDS mapping (Figs. S52 and S53). CV curves of Zn//NVO full cell employing ZS and ZS-POC electrolytes present two similar redox peaks (Fig. S54), which proves that the POC additive does not alter the NVO reaction mechanism. Using the ZS-POC electrolyte, Zn//NVO cell maintains cycling stability for over 1200 cycles with 99.6% capacity retention at 1 A g^−1^ (Fig. S55) and exhibits exceptional long-term cycling stability for over 10,000 cycles at 10 A g^−1^ (Fig. 7g). In contrast, the cell with ZS electrolyte experiences the rapid capacity degradation. Furthermore, Zn//NVO cells demonstrates excellent rate capability from 0.5 to 10 A g^−1^, with 100% capacity recovery upon returning to 1 A g^−1^ (Fig. S56). At practical condition, Zn//NVO cell with a high active a mass load of 23.45 mg cm^−2^ delivers the stable operation for over 618 cycles with capacity retention of 83.91% (Fig. 6h). Notably, Zn//NVO cell also displays the suppressed self-discharge behavior, as evidenced by the CE after a 48h rest period, where cell with ZS-POC electrolyte retains a CE of 92.53% compared to only 79.54% for the cell using the ZS electrolyte (Fig. S57). Additionally, a practical Zn//NVO pouch cell delivers a high initial capacity of 1 Ah and excellent cycling stability over 237 cycles, even under a high cathode mass loading of 26.77 mg cm^−2^ (7.25 mAh cm^−2^) and limited N/P ratio of 2.65 (Fig. 7i), underscoring the feasibility of the LSI design. The detailed parameters of the soft-pack battery are listed in Table S2.

## Conclusions

In this work, we first demonstrate in situ construction of robust interface via porous materials and design a unique in situ LSI with selective Zn^2+^ conduction channels and dynamic self-repairing capability via self-assembly of POC, advancing the development of practical AZIBs. Combining by experiments and theoretical calculations, we reveal the LSI originates from a delicate balance between POC-POC hydrogen-bonding and POC–anion/cation coordination, which stabilizes a porous and loosely packed architecture. In situ and ex situ characterizations demonstrate the designed LSI effectively restructures the EDL, raises the Zn^2^⁺ transference number to 0.8, accelerates desolvation kinetics and homogenizes the interfacial electric field, thereby guiding dendrite-free Zn deposition along (002) crystal plane orientation. Meanwhile, LSI confines the activity of interfacial water and enhances the hydrophobicity of the interface, thereby collectively inhibiting HER and the formation of by-products. Consequently, Zn//Zn symmetric cells achieve an ultra-long cycling life span of 3200 cycles at 50 mA cm^−2^ and Zn//NVO full cell retains stable operation for over 10,000 cycles at 10 A g^−1^. Notably, the practical Zn//NVO pouch cell delivers a high initial capacity of 1 Ah and excellent cycling stability over 237 cycles under a high cathode mass loading of 26.77 mg cm^−2^ (7.25 mAh cm^−2^) and limited N/P ratio of 2.65. This study offers a general methodology for building highly conductive interfaces using porous materials and paves the way toward next-generation battery systems.

## Supplementary Information

Below is the link to the electronic supplementary material.

Supplementary file1 (DOCX 17696 KB)
