# Supplementary material for: Engineering In Situ Loose Selective Interface with Conducting Channels for Practical Ah-Level Aqueous Zinc Metal Batteries
Source: Nanomicro Lett. 2026 Jul 8;18:431. doi: 10.1007/s40820-026-02264-y (PMC13346383; doi:10.1007/s40820-026-02264-y)
Supplement: Supplementary file 1 — Supplementary file1 (DOCX 17696 KB) [file 40820_2026_2264_MOESM1_ESM.docx]

Supporting Information for

**Engineering In-Situ Loose Selective Interface with Conducting Channels for Practical Ah-Level Aqueous Zinc Metal Batteries**

Dongdong Wang^1, 2^, Shuo Dou^1,^ *, Qiongying Huang^2^, Jiaying Li^1^, Mi Xu^2^, Jingshuai Li^2^, Zhendong Guo^2^, Siqi Qin^2^, Tieyan Wang^2^, Haipeng Yu^1^, Haozhen Dou^2,^ *, and Zhongwei Chen^2,^ *

^1^State Key Laboratory of Utilization of Woody Oil Resource, Key Laboratory of Bio-Based Material Science and Technology of Ministry of Education, Northeast Forestry University, Harbin 150040, P. R. China

^2^Power Battery & Systems Research Center, State Key Laboratory of Catalysis, Dalian Institute of Chemical Physics, Chinese Academy of Sciences, Dalian 116023, P. R. China

*Corresponding authors. E-mail: doushuo@nefu.edu.cn (Shuo Dou); haozhen@dicp.ac.cn (Haozhen Dou); zhwchen@dicp.ac.cn (Zhongwei Chen)

**S1 Materials Characterization**

The electrode morphology was analyzed using field emission scanning electron microscopy (FE-SEM, JEOL, JSM-6510), which enabled the observation of material morphologies at high resolution. X-ray diffraction (XRD) measurements were conducted using a Bruker D8 Advance instrument over the 2θ range of 5 to 90°. Chemical bonding characteristics and the solvation structure of Zn^2+^ in both ZF and various DMEEs were explored using diverse analytical methods. The techniques employed for data acquisition included Fourier transform infrared spectroscopy (FTIR) (BRUKER TENSOR II), nuclear magnetic resonance H spectroscopy (Bruker 400M), and Raman microscopy (Renishaw in Via). Focused ion beam (FIB) was applied on the FEI Scios microscope to observe the cross-section of deposited zinc. X-ray photoelectron spectroscopy (XPS) was performed using the Thermo Scientific ESCALAB Xi^+^ spectrometer to analyze surface and depth chemical compositions. The Ar^+^ etching rate was estimated to be 1.0 nm·min^-1^. Time-of-flight secondary ion mass spectrometry (TOF-SIMS) was performed on the TOF–SIMS5-100 spectrometer for in-depth analysis of element distribution. During this process, a 1 KeV Cs^+^ ion beam was used for sputtering.

The Zn²⁺ transference number ($t_{{Zn}^{2+}}$) was determined using a combination of chronoamperometry (CA) and electrochemical impedance spectroscopy (EIS) based on the Bruce–Vincent–Evans method. The detailed procedure has now been added to the revised manuscript and is described as follows:

Symmetric Zn||Zn cells were assembled using the corresponding electrolyte and separator. A small DC polarization voltage (20 mV) was applied to the cell, and the current response was recorded over time until a steady-state current was reached. Meanwhile, EIS measurements were conducted before and after polarization to obtain the initial resistance (R₀) and steady-state resistance (Rs), respectively.

The Zn^2+^ transference number was then calculated according to:

$$t_{{Zn}^{2+}}=\frac{I_{s}(\Delta V-I_{0}R_{0})}{I_{0}(\Delta V-I_{s}R_{s})}$$

where I₀ and I_S_ are the initial and steady-state currents, respectively, ΔV is the applied polarization voltage, and R₀ and R_S_ are the interfacial resistances before and after polarization.

All measurements were conducted at room temperature, and the cells were allowed to rest for sufficient time prior to testing to ensure equilibrium. This method has been widely used to evaluate ion transport behavior in electrolyte systems.

The electrochemical impedance spectroscopy (EIS) at different temperatures from 30 to 80 ºC was used to elaborate on the Zn^2+^ desolvation and transport behavior, which can be calculated according to Arrhenius equation:

$$\frac{1}{R_{ct}}=A exp(-\frac{E_{a}}{RT})$$

where R_ct_, A, R, E_a_, and T represent the interfacial resistance, pre-exponential factor, the gas constant, activation energy, and temperature, respectively.

**S2 Computational methods**

**S2.1 Binding energy and electrostatic potentials (ESP) calculation by Dmol3**

The binding energies between H_2_O, SO_4_^2-^, Zn^2+^, POC and POC as well as corresponding electrostatic potentials (ESP) and HOMO/LUMO were respectively calculated by Dmol3 module in Materials Studio (MS) 2020. Firstly, the generalized gradient approximation (GGA) with Perdew–Burke–Ernzerhof (PBE) exchange-correlation functional was employed to fully relax H_2_O, SO_4_^2-^, Zn^2+^ and POC. The double-numeric quality basis sets with polarization functions were used. The iterative tolerances for energy change, force and displacements were 1 × 10^−5^ Ha, 0.002 Ha Å^−1^ and 0.005 Å, respectively. In the self-consistent field (SCF) procedure, 10^−6^ a.u. was used for the convergence standard electron density. After structure optimization, the Adsorption Locator Tools [S1] in MS were used to construct interaction pairs. Then each interaction pair were freely optimized by Dmol3 module. Finally, single point energy calculation was executed. The binding energies *E_b_* [S2, S3] were calculated according to the following equation:

$$E_{b}=E_{total}-E_{POC}-E_{B}$$

Where *E_total_* is the total energy of the optimized complex structure, *E_POC_* is the energy of POC in interaction pair, *E_B_* is the energy of another component in interaction pair, namely H_2_O, SO_4_^2-^, Zn^2+^ and POC. The HOMO and LUMO of H_2_O and POC were also calculated by Dmol3 module in MS 2020.

**S2.2 Adsorption energy calculation by VASP**

The adsorption calculations for H_2_O and POC adsorption on Zn (002) surface were separately performed within the framework of density functional theory (DFT) as implemented in the Vienna *Ab-initio* Simulation Package (VASP) code by using the projector augmented wave method with the Perdew-Burke-Ernzerhof (PBE) exchange-correlation functional [S4-S6]. The influence of vdW interactions is considered by using a modified version of vdW-DF, referred to as “optB86b-vdW” [S7, S8]. The projector augmented wave potentials [S9] were used with an energy cutoff of 600 eV. The lattice constraints for Zn (002) slab which is composed of 2 layers Zn is 20.86 Å × 20.86 Å. There exists a vacuum layer of large than 25 Å perpendicular to the surface plane. A 3 × 3 × 1 Monkhorst method *k*-mesh was used for geometry optimization during which the bottom layer of Zn substrate was fixed. Energy convergence of 1.0 × 10^-4^ me V/atom was ensured during the self-consistent field calculations. And the convergence criteria for the atomic forces was 0.01 eV/Å. After structure optimization, H_2_O and POC were respectively adsorbed on Zn (002) surface_._ Geometry optimization was then performed for each adsorption system followed by static calculation. The *k*-meshes were doubled for single-point calculation. The adsorption energies *E_ad_* was calculated according to the following equation:

$$E_{ad}=E_{total}-E_{solvent}-E_{slab}$$

Where *E_total,_ E_solvent_* and *E_slab_* are the energies of the adsorption system, adsorbed solvent, i.e., H_2_O or POC and Zn (002) slab, respectively. The more negative adsorption energy means the more stable adsorption configuration. For the most stable configuration of POC adsorption on Zn (002) surface, charge density difference and corresponding slice display were visualized by VESTA.

**S2.3 Diffusion energy barrier of Zn^2+^ along POC**

The diffusion energy barrier of Zn^2+^ along POC structure was evaluated based on density functional theory (DFT). All DFT calculations were carried out using Dmol3 module in MS 2020. Here, the generalized gradient approximation (GGA) with Perdew–Burke–Ernzerhof (PBE) exchange-correlation functional was employed. The double-numeric quality basis sets with polarization functions were used. The iterative tolerances for energy change, force and displacements were 1 × 10^−5^ Ha, 0.002 Ha Å^−1^ and 0.005 Å, respectively. In the self-consistent field (SCF) procedure, 10^−6^ a.u. was used for the convergence standard electron density. After structure optimization, the Adsorption Locator Tools in MS were used to locate Zn^2+^ at two different sites of POC. Then each adsorption structure was freely optimized by Dmol3 module serving as initial state (IS) and final state (FS) structure, respectively. Finally, the complete LST/QST method in Dmol3 module was adopted to conduct transition state (TS) search and obtain diffusion energy barrier.

**S2.4 Desolvation energy calculation**

The step-by-step desolvation energy barriers of solvation sheath [Zn (H_2_O)_6_]^2+^ with POC present or not were evaluated based on density functional theory (DFT) by utilizing Dmol3 module in MS 2020. The generalized gradient approximation with Perdew–Burke–Ernzerhof exchange-correlation functional was employed to fully relax solvation shells. The double-numeric quality basis sets with polarization functions were used. The iterative tolerances for energy change, force and displacements were 1 × 10^−5^ Ha, 0.002 Ha Å^−1^ and 0.005 Å, respectively. In the self-consistent field (SCF) procedure, 10^−6^ a.u. was used for the convergence standard electron density. The desolvation process of POC and hydrated zinc ions consists of the following five steps: [Zn^2+^·6(H_2_O)]@POC→[Zn^2+^·5(H_2_O)]@POC→[Zn^2+^·4(H_2_O)]@POC→[Zn^2+^·3(H_2_O)]@POC → [Zn^2+^·2(H_2_O)]@POC → [Zn^2+^·1(H_2_O)]@POC → [Zn^2+^]. The desolvation energy barrier for each step is illustrated using the example of [Zn^2+^·6(H_2_O)]@POC being dehydrated to [Zn^2+^·5(H_2_O)]@POC and H_2_O, the free energy evolution (∆G) can be obtained from the following formula: ∆G = G[Zn^2+^·5(H_2_O)]@POC +G[H_2_O] - G[Zn^2+^·6(H_2_O)]@POC [S10]. The Gibbs free energy of reaction species is derived from ∆*G* = ΔE + Δ*E*_ZPE_ – *T*Δ*S*, where ΔE, Δ*E*_ZPE_ and Δ*S* are the changes in electronic energy, zero-point energy and entropy, respectively.

**S2.5 Deprotonation Energy calculation**

The deprotonation energies of solvation structure Zn (H_2_O)_6_^2+^ with POC presence or not were respectively calculated by Dmol3 module in Materials Studio 2023. Firstly, the generalized gradient approximation with Perdew–Burke–Ernzerhof exchange-correlation functional was employed to fully relax solvation structure Zn (H_2_O)_6_^2+^ and Zn (H_2_O)_6_^2+^@POC. The double-numeric quality basis sets with polarization functions were used. The iterative tolerances for energy change, force and displacements were 1 × 10^−5^ Ha, 0.002 Ha Å^−1^ and 0.005 Å, respectively. In the self-consistent field procedure, 10^−6^ a.u. was used for the convergence standard electron density. After structure optimization, single point energy calculation was executed [S11]. The deprotonation energies *E_d_* was calculated according to the following equation:

$$E_{d}=E_{SS^{-}}+E_{H^{+}}-E_{SS}$$

Where *E_SS_* is the total energy of the optimized solvation structure, *E_SS_^-^* is the energy of optimized solvation structure after deprotonation, *E_H_^+^* is the energy of the proton.

**S3 COMSOL simulation calculation**

Finite element simulations were performed using COMSOL Multiphysics 16.2 software. The Zn²⁺ concentration in the electrolyte was set to 2 M, and the Butler-Volmer equation was used to solve the surface reaction kinetics at the lower boundary. The ion flux at the electrolyte interface was coupled to the electrochemical reaction according to Faraday's law. The electric field-driven Zn²⁺ migration was described by the Nernst-Planck equation. The simulation region near the Zn electrode was set to 140 × 150 μm. The model was calculated using the MUMPS transient solver in COMSOL Multiphysics. The governing equations are as follows:

Mathematical description of the Nernst-Planck equations:

$$N_{i}=-D_{i}\nabla c_{i}- \mu_{i}c_{i}\nabla\varphi, i=1, 2\ldots,$$

Where Ni and Di represent the flux and diffusion coefficient of a specific ion, and i represents the component. μ_i_, c_i_, and φ are the ion mobility, ion concentration, and electrolyte potential, respectively.

**S4 Calculation of the consumption rate of POC additives**

In the assembly procedure of the Zn//Zn symmetric battery, the amount of ZS-POC electrolyte is 80 μL (with a POC concentration of 10 mg mL^-1^). Based on the addition amount and the cycle period, its consumption rate is estimated to be approximately the mass of POC divided by the cycle life of the battery. Therefore, we initially estimate its consumption rate to be about 0.0017 mg h^-1^.

**S5 Supplementary Figures**


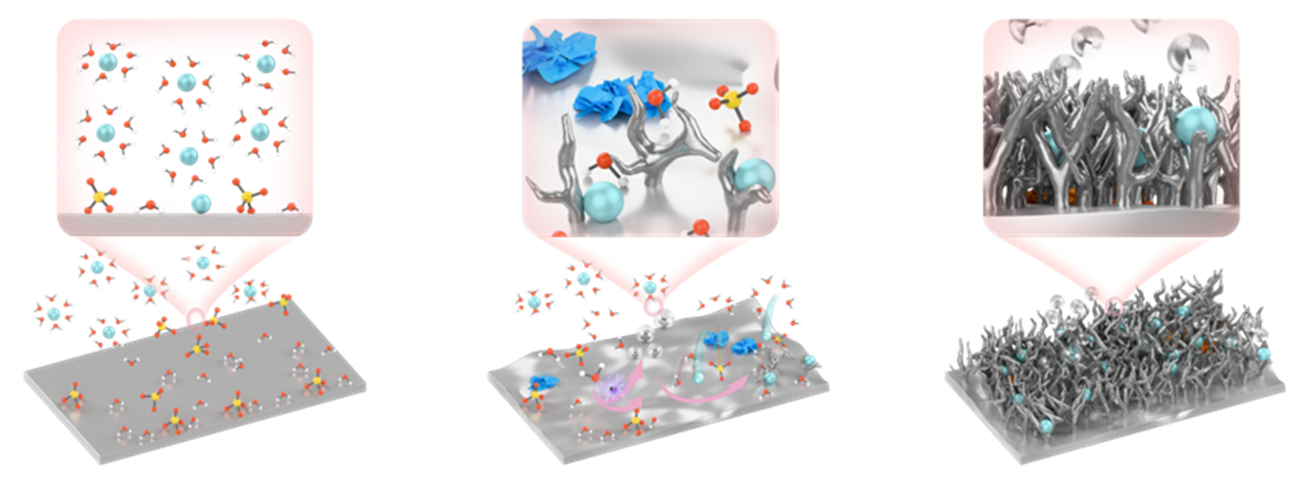


**Fig. S1** In ZS electrolyte, the stripping/deposition process of Zn²⁺ exacerbates problems such as dendrite growth, hydrogen evolution, and byproduct formation at the zinc anode.


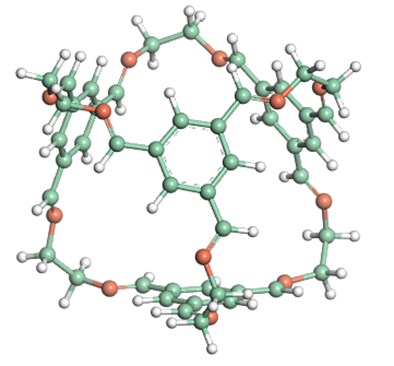


**Fig. S2** Molecular structure of POC.


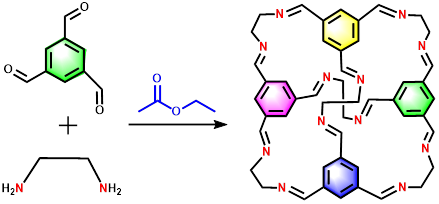


**Fig. S3** Synthesis process of POC.


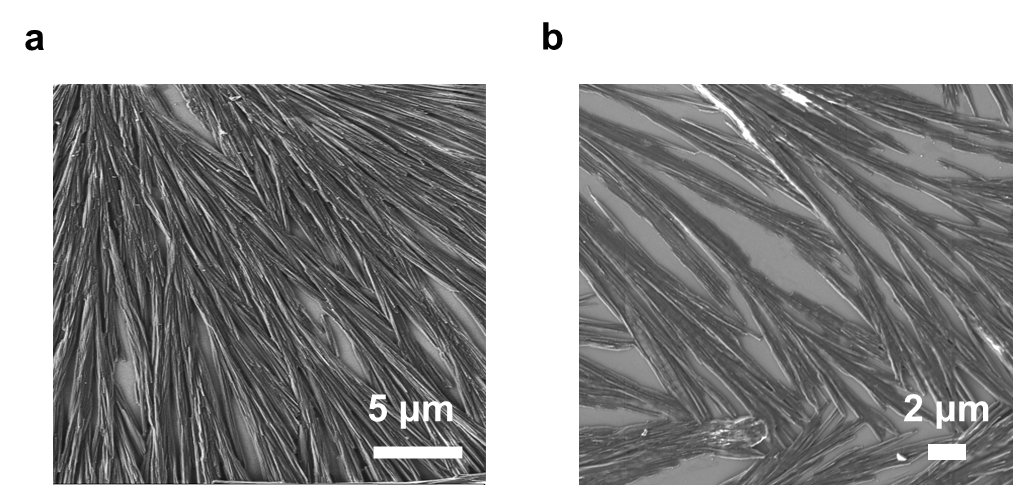


**Fig. S4** SEM images of POC.


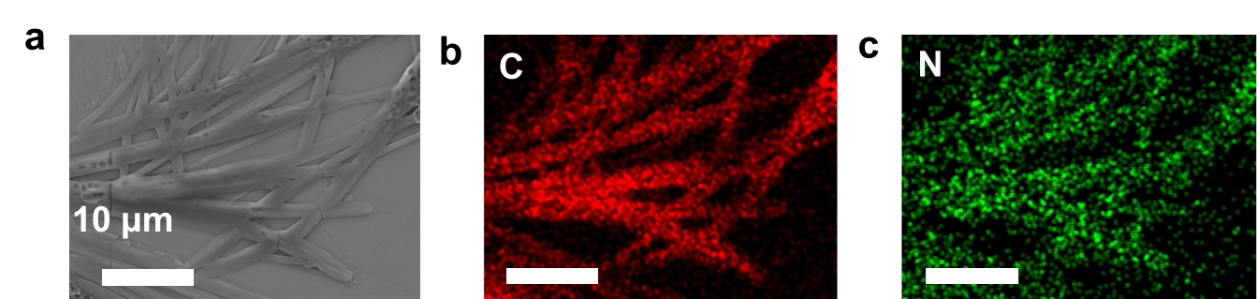


**Fig. S5 a** SEM images of POC; EDS mapping images of **b** C and **c** N in POC molecules.


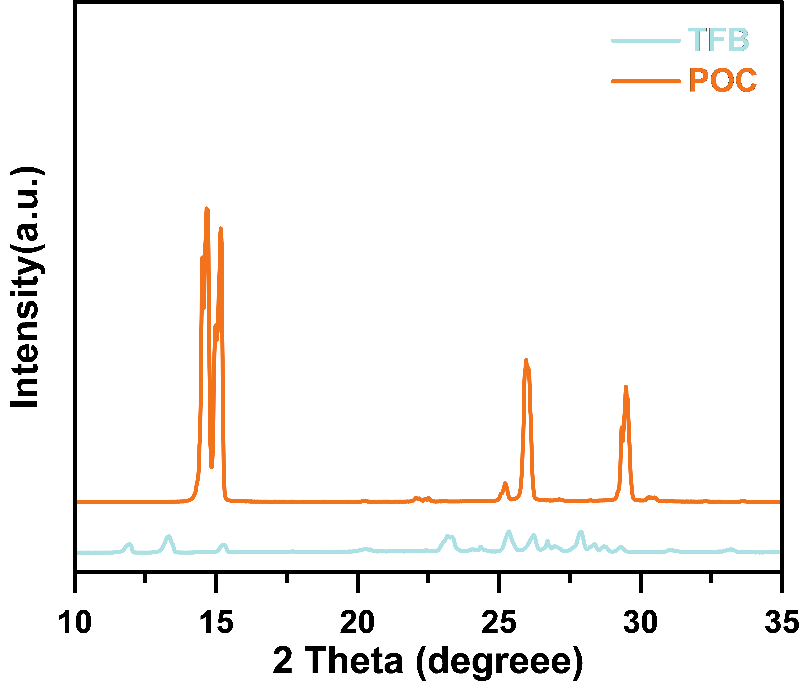


**Fig. S6** XRD pattern of the POC.

**Fig. S7** ATR-FTIR spectra of the EDA, TFB and POC.


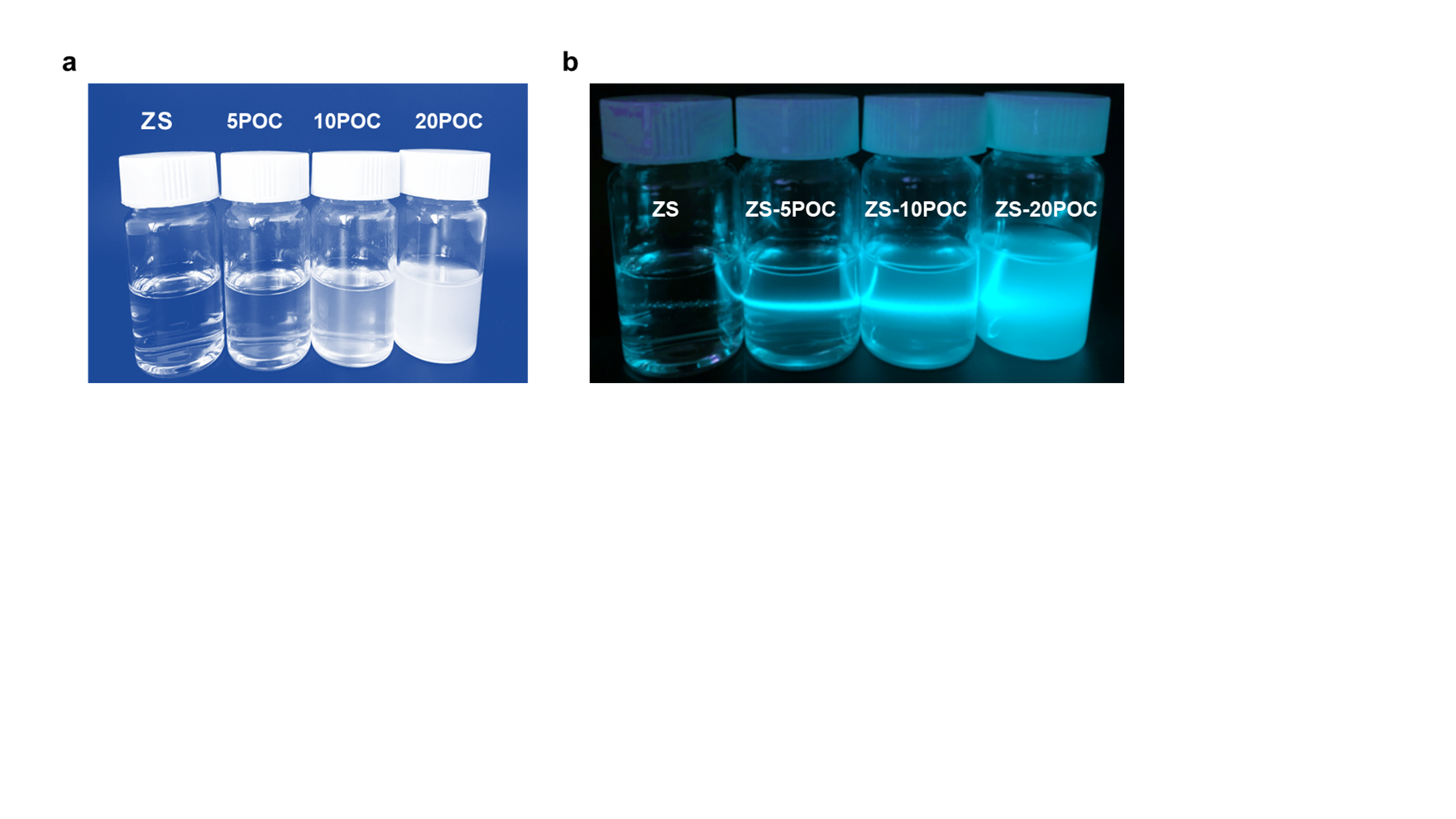


**Fig. S8** **a** Digital photographs and **b** Tyndall effect optical path diagrams of ZS, ZS-5POC, ZS-10POC and ZS-20POC electrolytes.


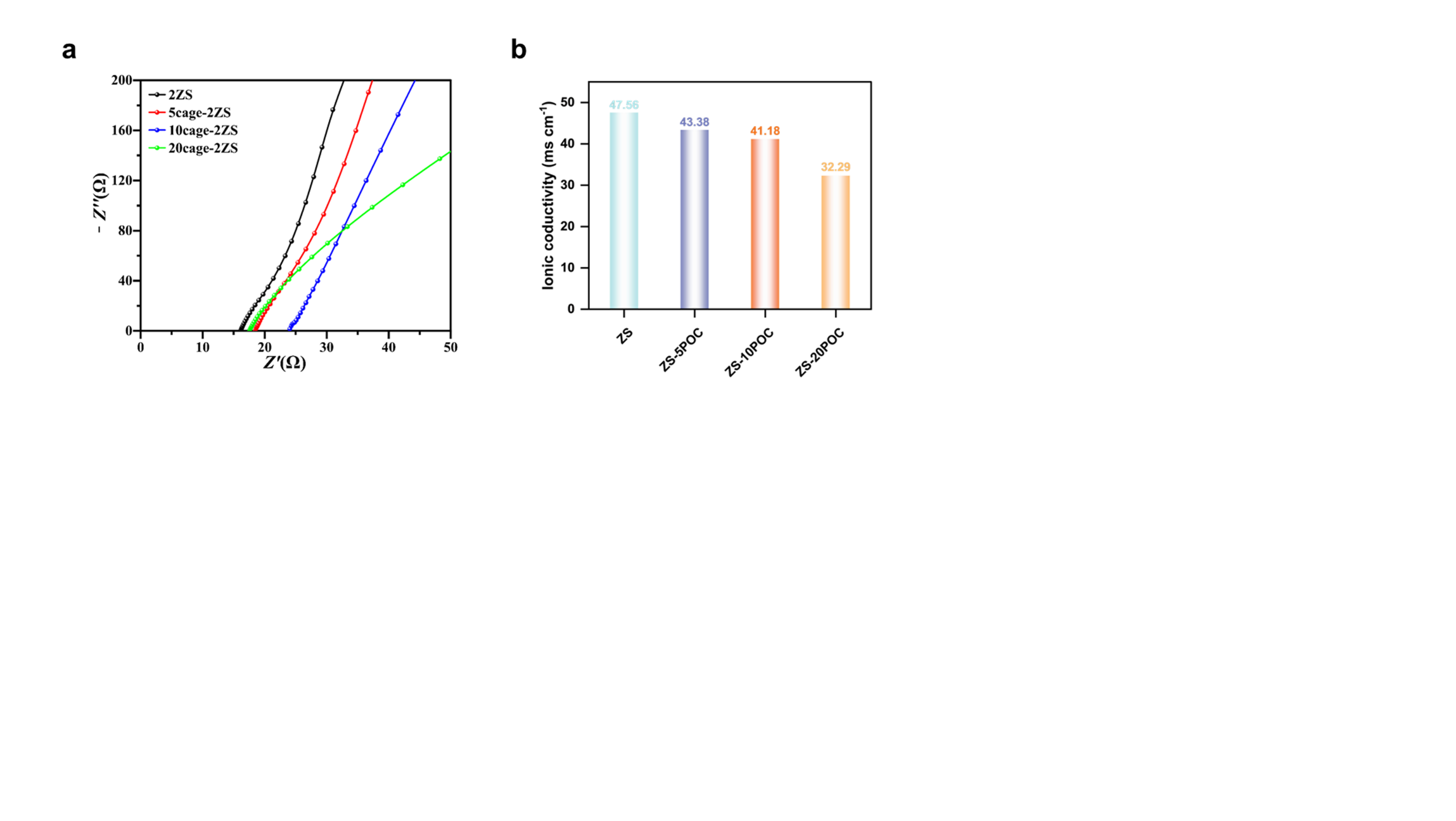


**Fig. S9** **Ionic conductivity of ZS and ZS-POC electrolytes.** EIS curves and **b** ion conductivities of the ZS and ZS-5POC, ZS-10POC and ZS-20POC electrolytes.


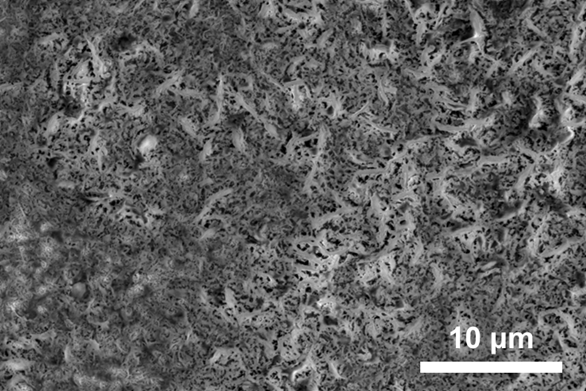


**Fig. S10** FIB**-**SEM images of self-assembled POC film and LSI.


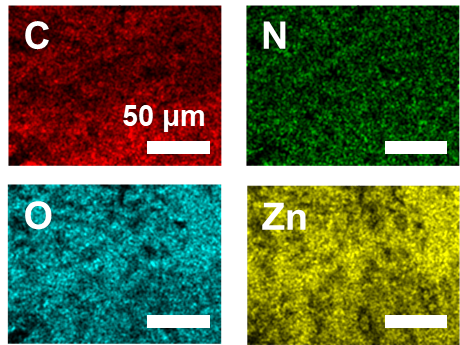


**Fig. S11** EDS images mapping of films formed from POC-H_2_O solutions on zinc foil.


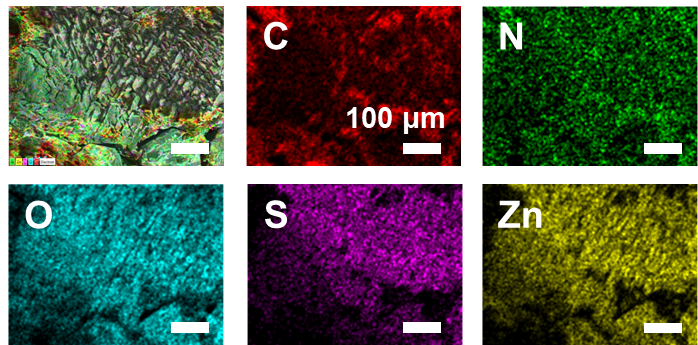


**Fig. S12** SEM and EDS mapping images of films formed from solutions on zinc foil.


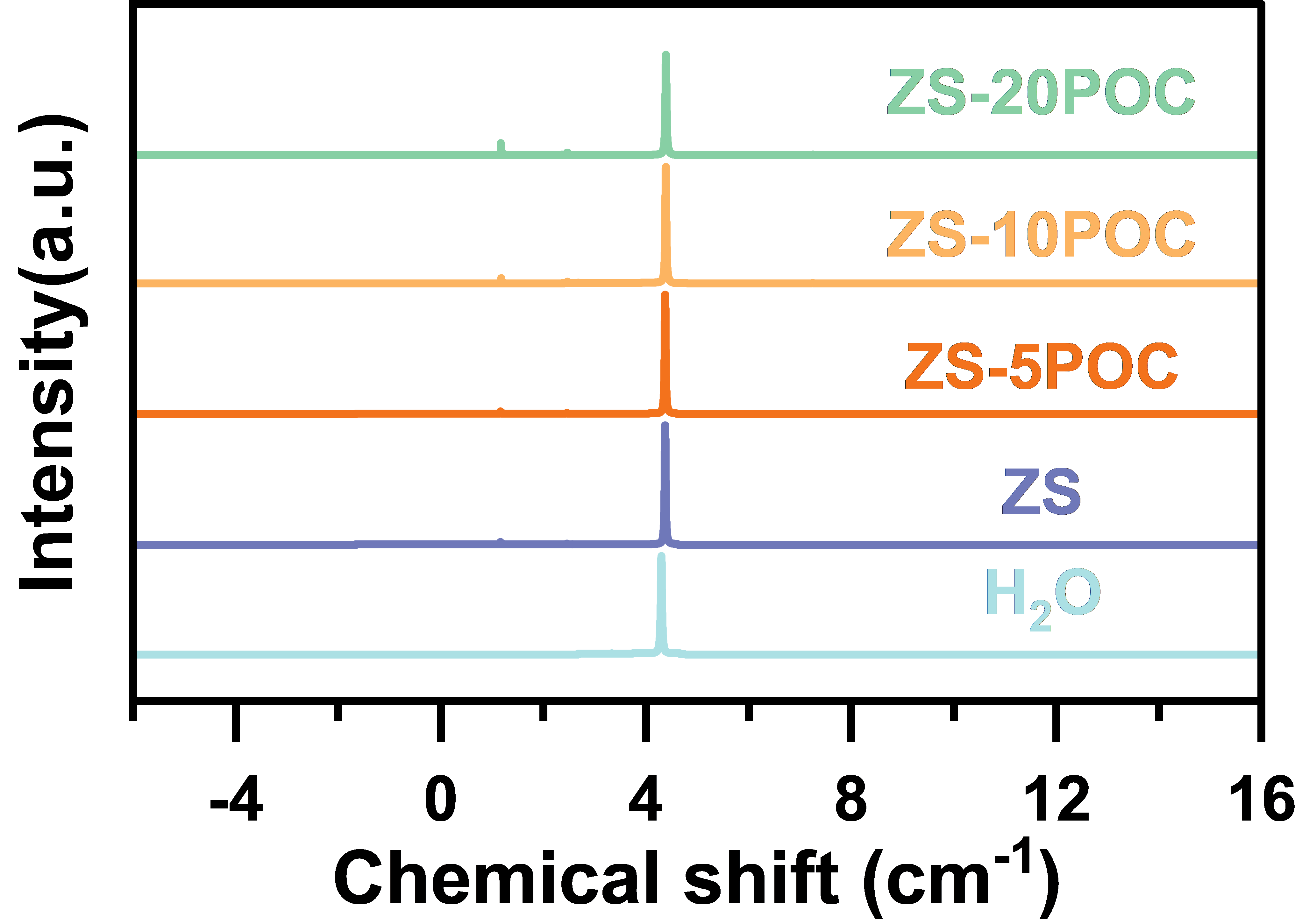


**Fig. S13** ^1^H NMR spectra of H_2_O, ZS, ZS-5POC, ZS-10POC and ZS-20POC electrolytes.


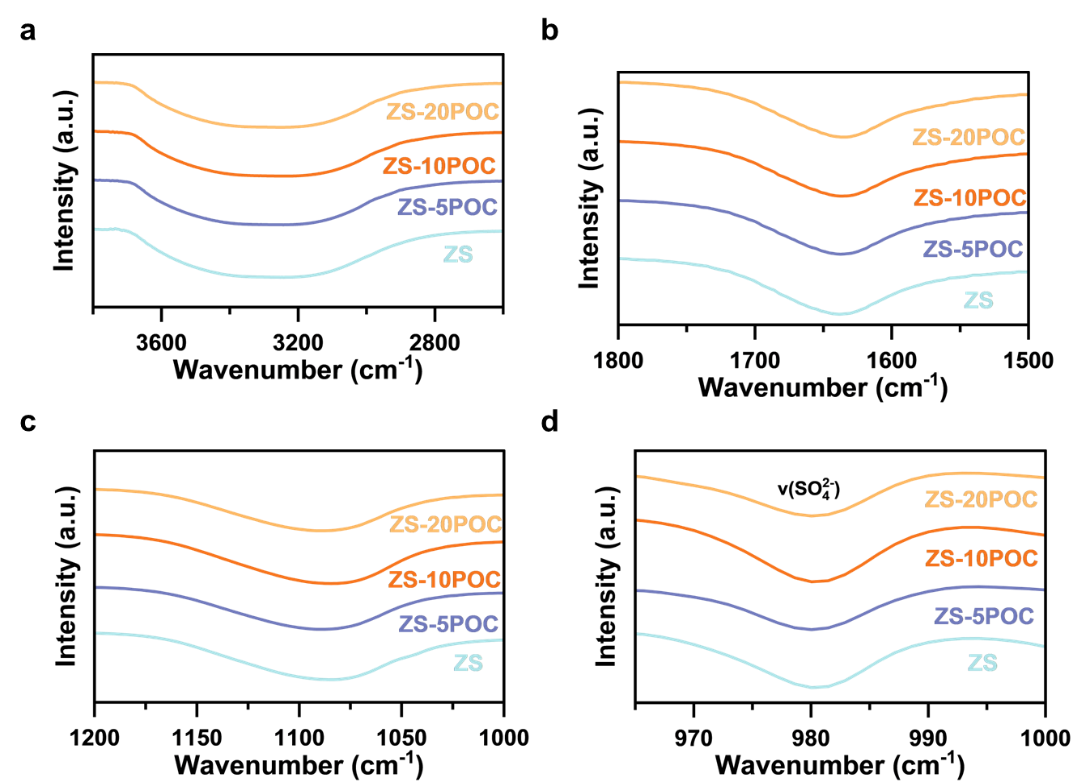


**Fig. S14** ATR-FTIR spectra of ZS, ZS-5POC, ZS-10POC and ZS-20POC electrolytes.


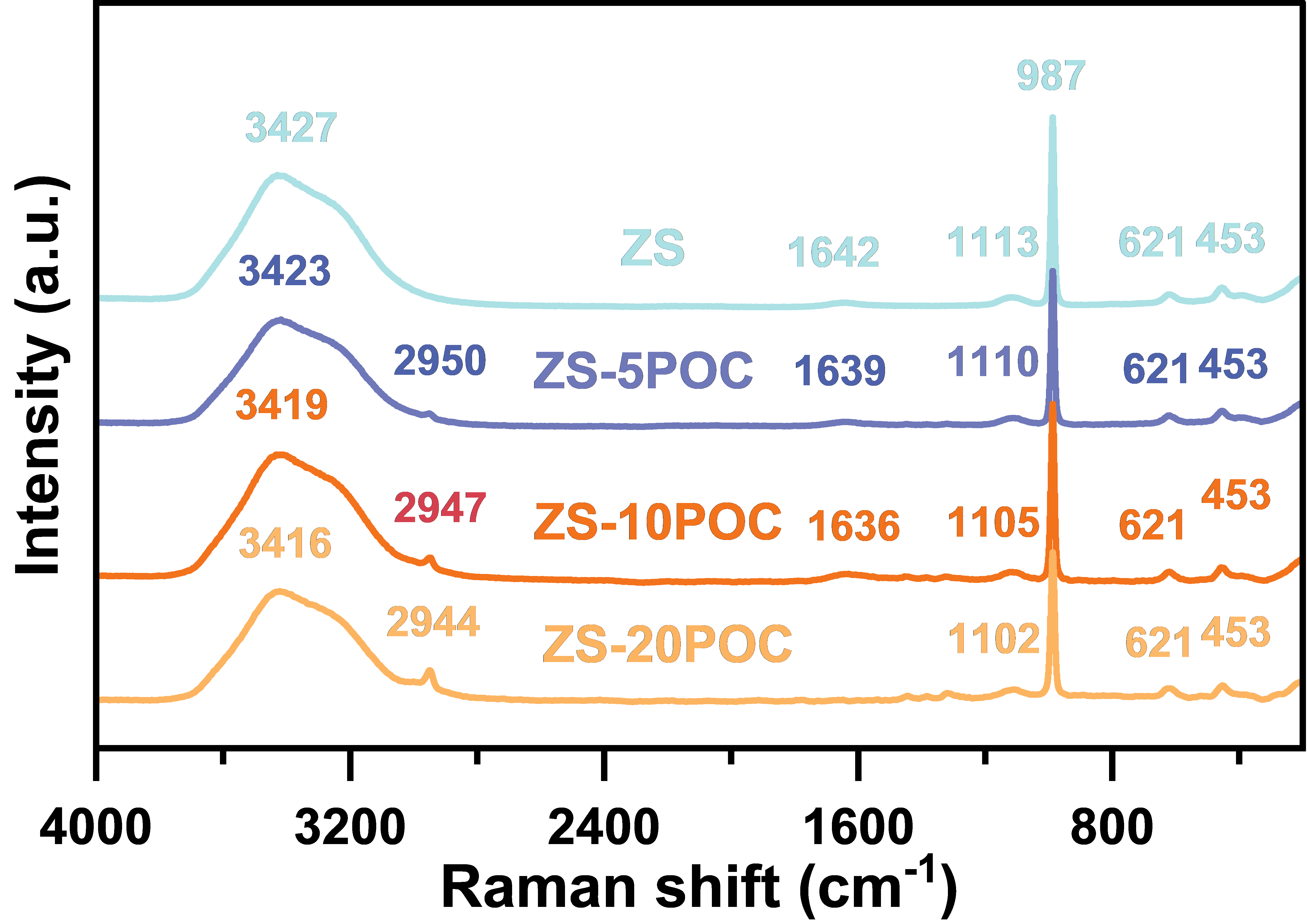


**Fig. S15** Raman spectra of ZS, ZS-5POC, ZS-10POC and ZS-20POC electrolytes.


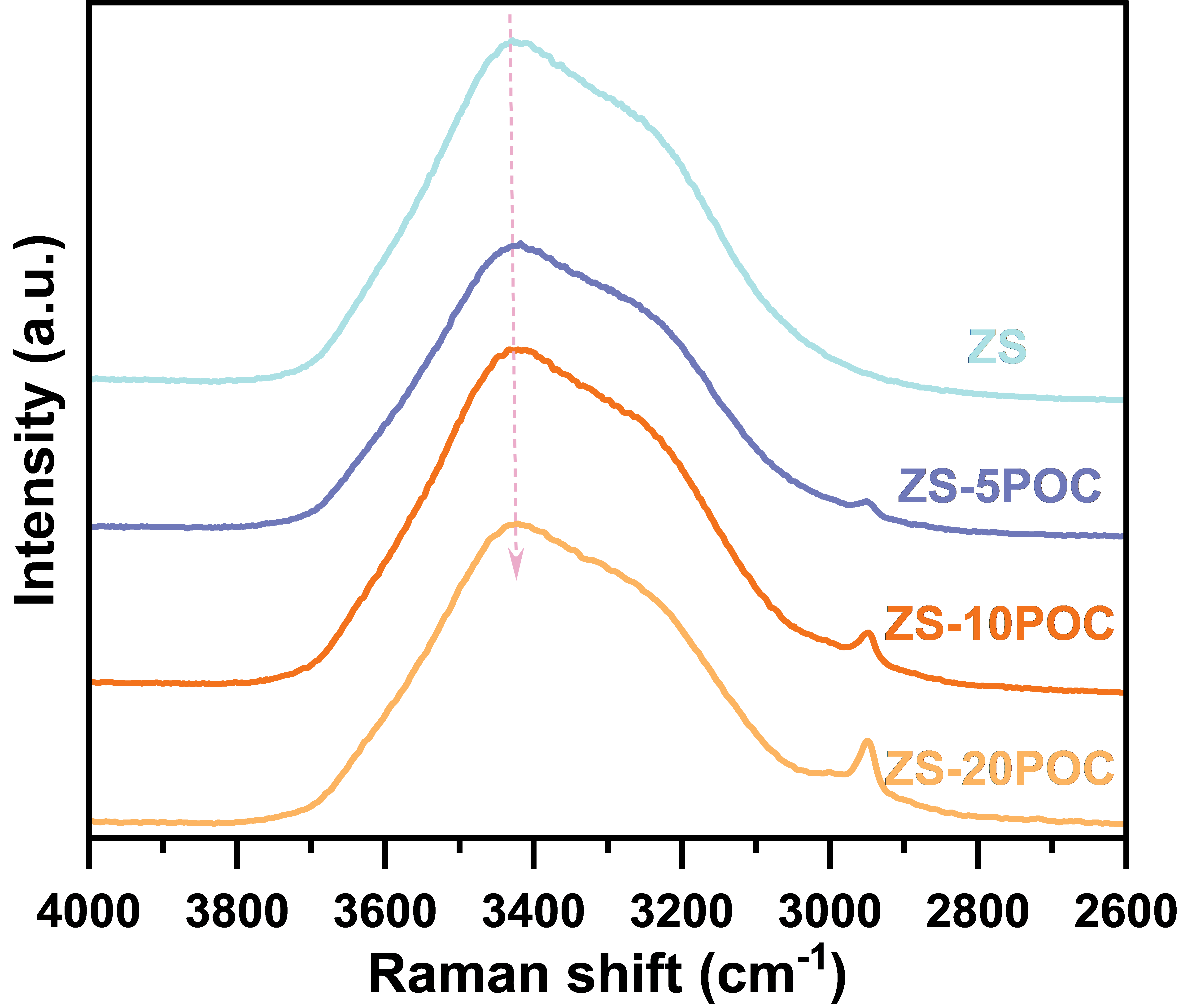


**Fig. S16** Raman spectra of v-OH in the different electrolytes.


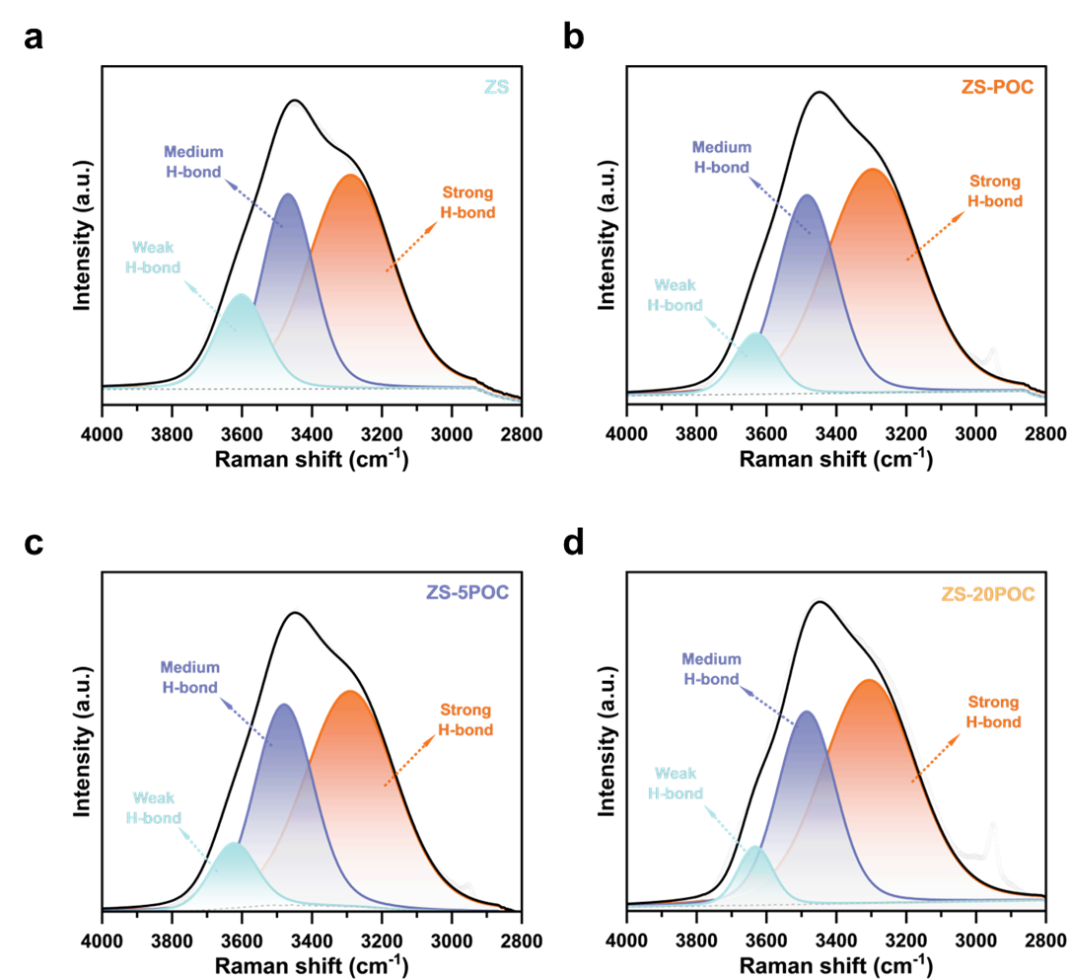


**Fig. S17** Fitted Raman spectra of v-OH in the different electrolytes.


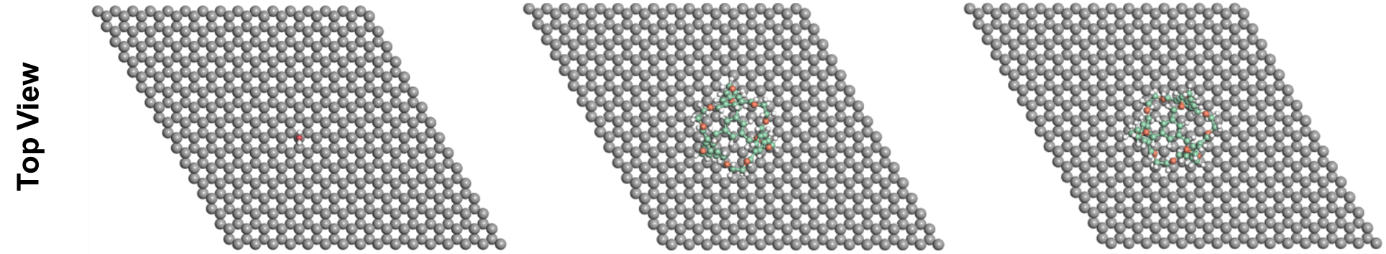


**Fig. S18** Adsorption configuration of H_2_O, POC1 and POC2 on the Zn (002) plane (Top view).


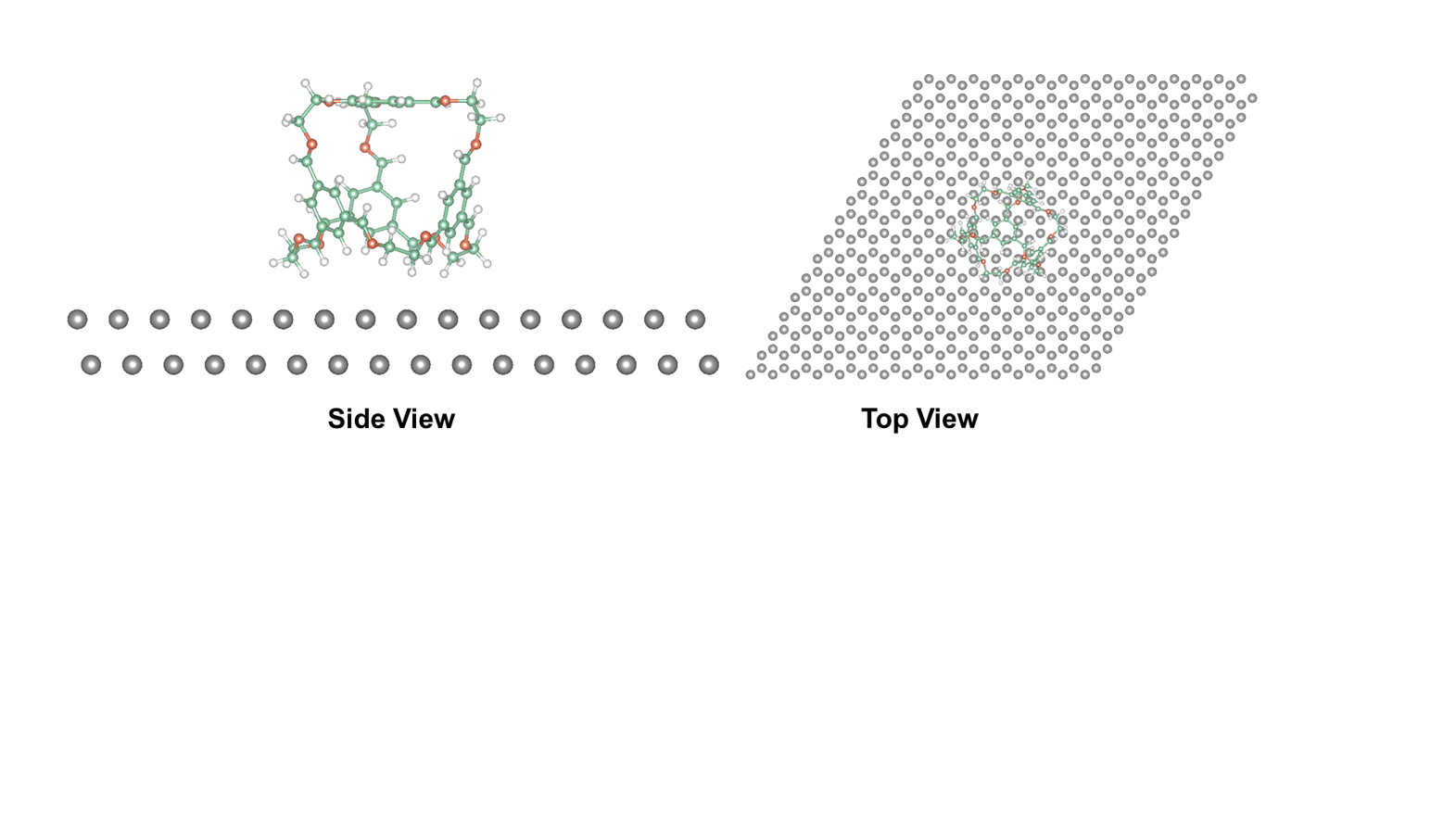


**Fig. S19** Charge density difference of the Zn (002) with POC adsorption (Side and Top view).


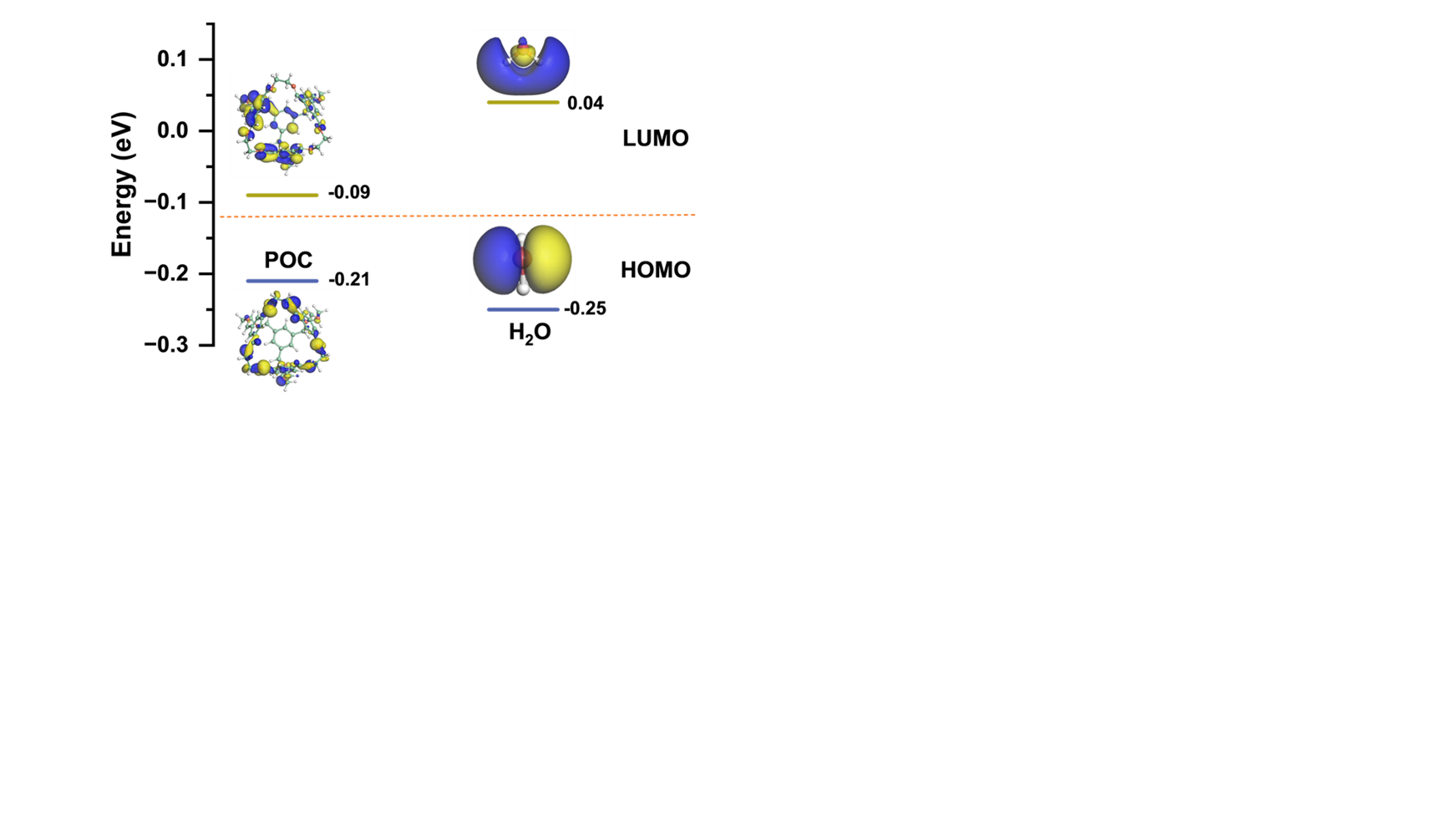


**Fig. S20** LUMO, HUMO iso-surfaces of POC (left) and H_2_O molecules (right).


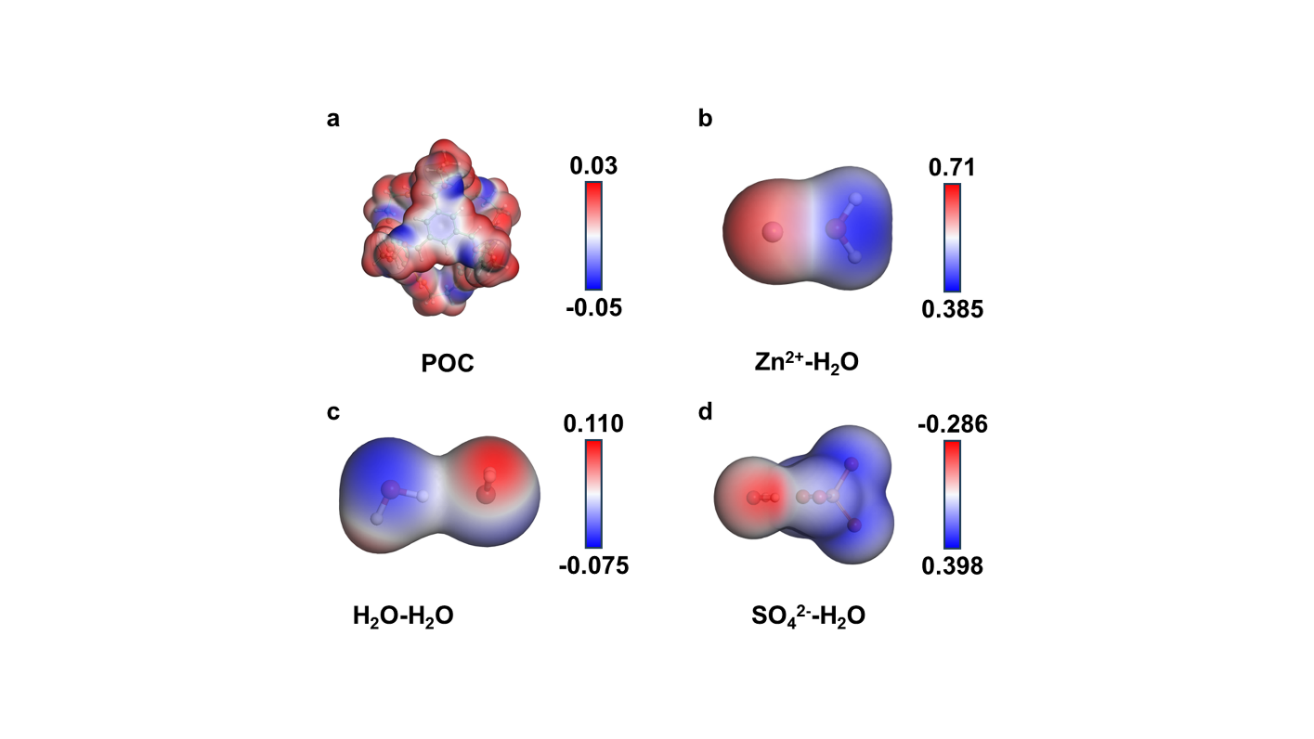


**Fig. S21** ESP potential distribution and binding energy of **a** POC, **b** Zn^2+^-H_2_O, **c** H_2_O - H_2_O, **d** SO_4_^2-^-H_2_O.


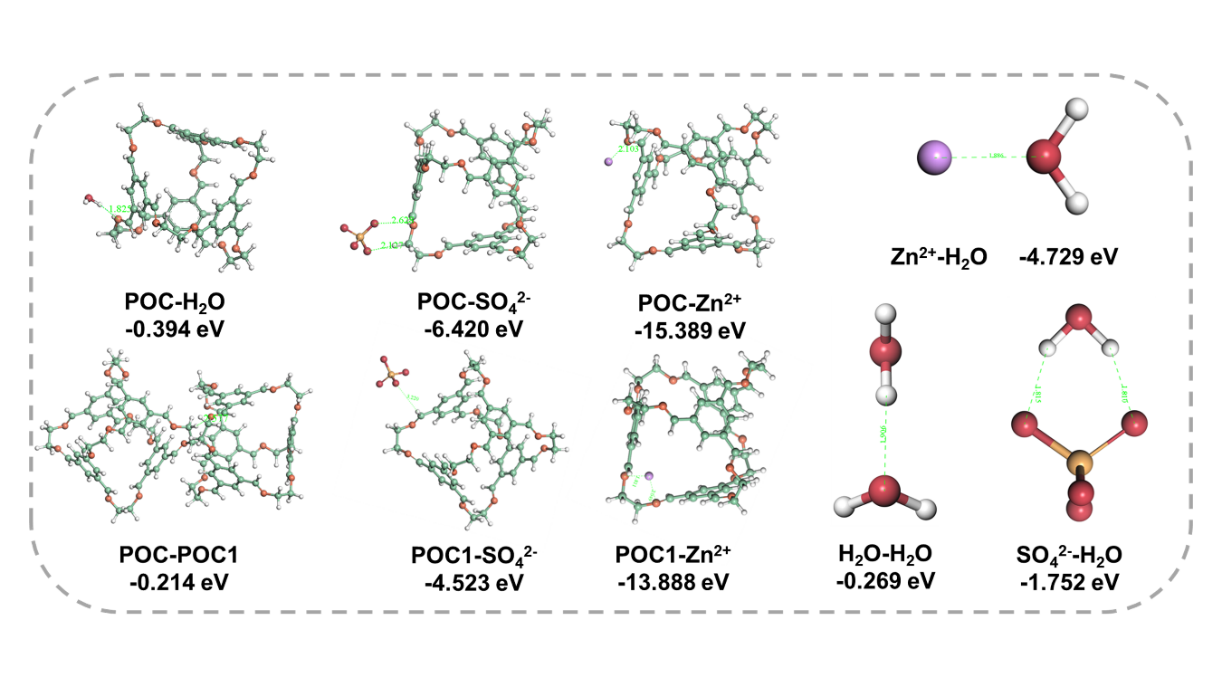


**Fig. S22** Interaction energies between components in ZS-POC electrolytes calculated by DFT.


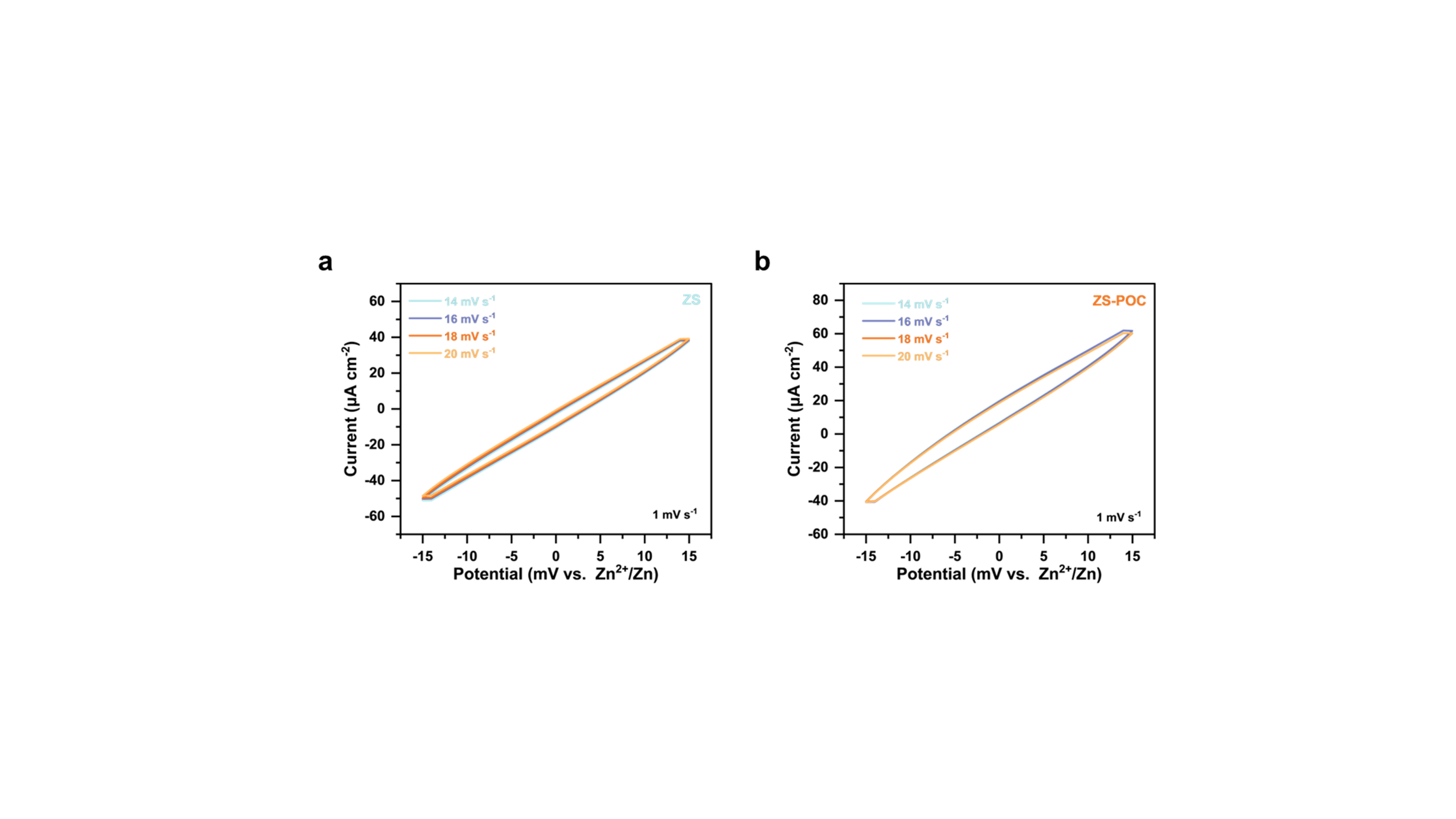


**Fig. S23** CV curves and double layer capacitance of Zn//Zn symmetric cells in ZS and **b** ZS-POC electrolytes.


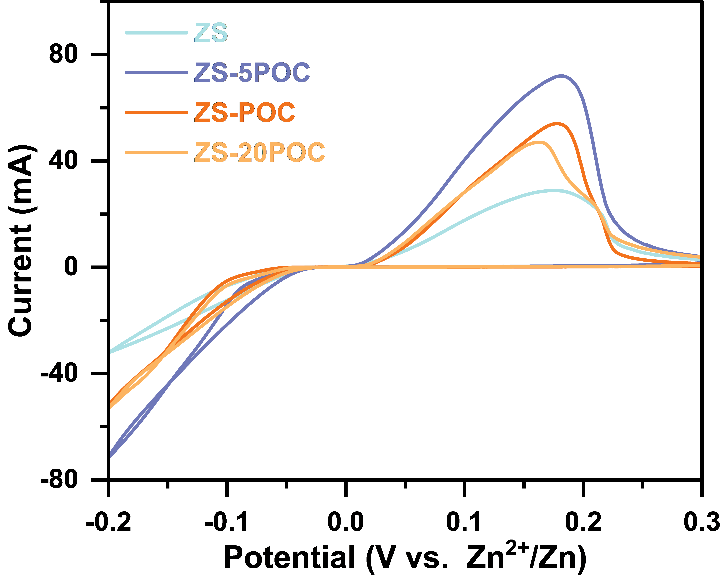


**Fig. S24** CV curves for different electrolytes measure the Nucleation Overpotential (NOP) of the ZS electrolyte containing different concentrations of POC at a scan rate of 1 mVs^-1^_._

_
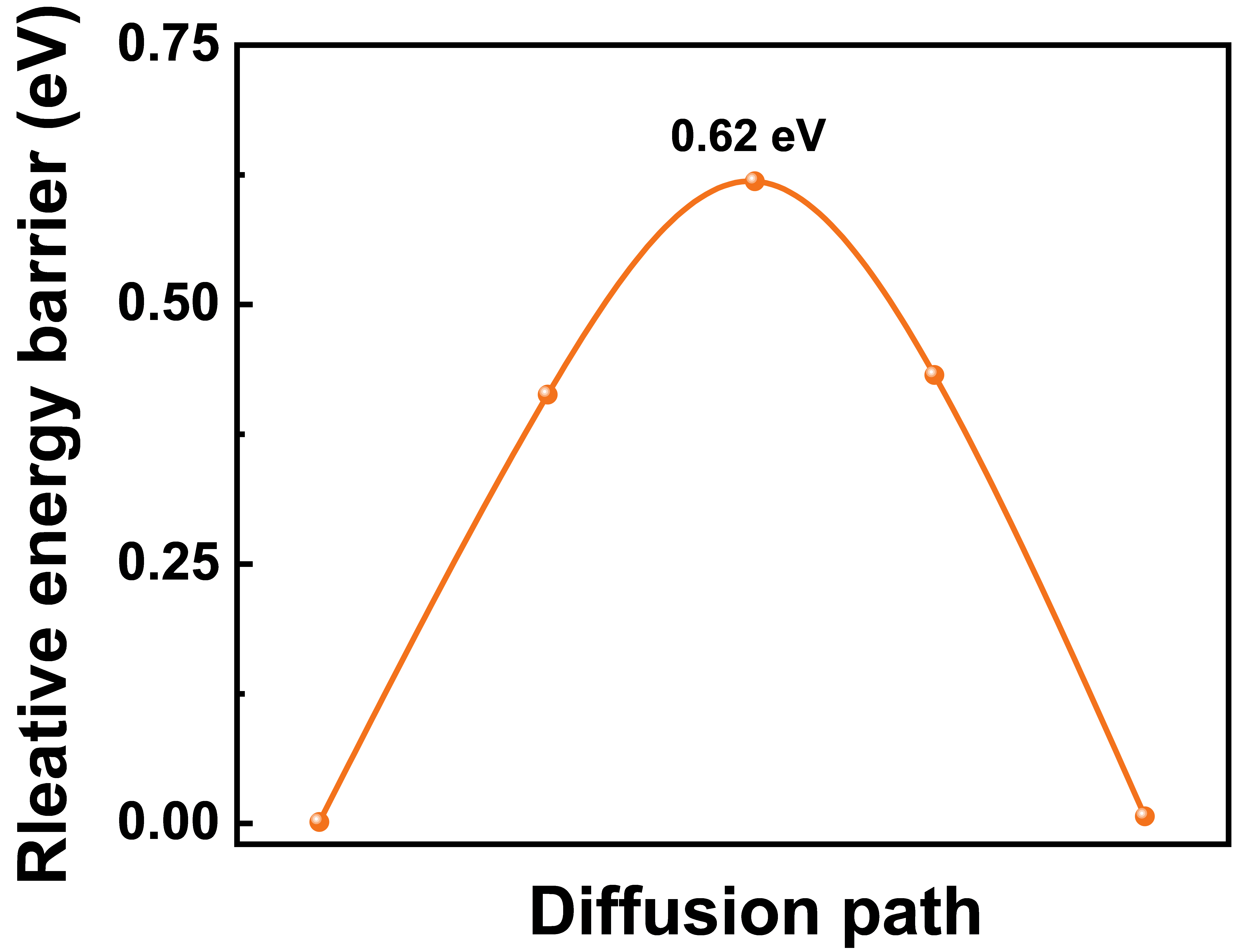
_

**Fig. S25** Diffusion energy barrier of Zn^2+^ ion between POC polymeric chains.


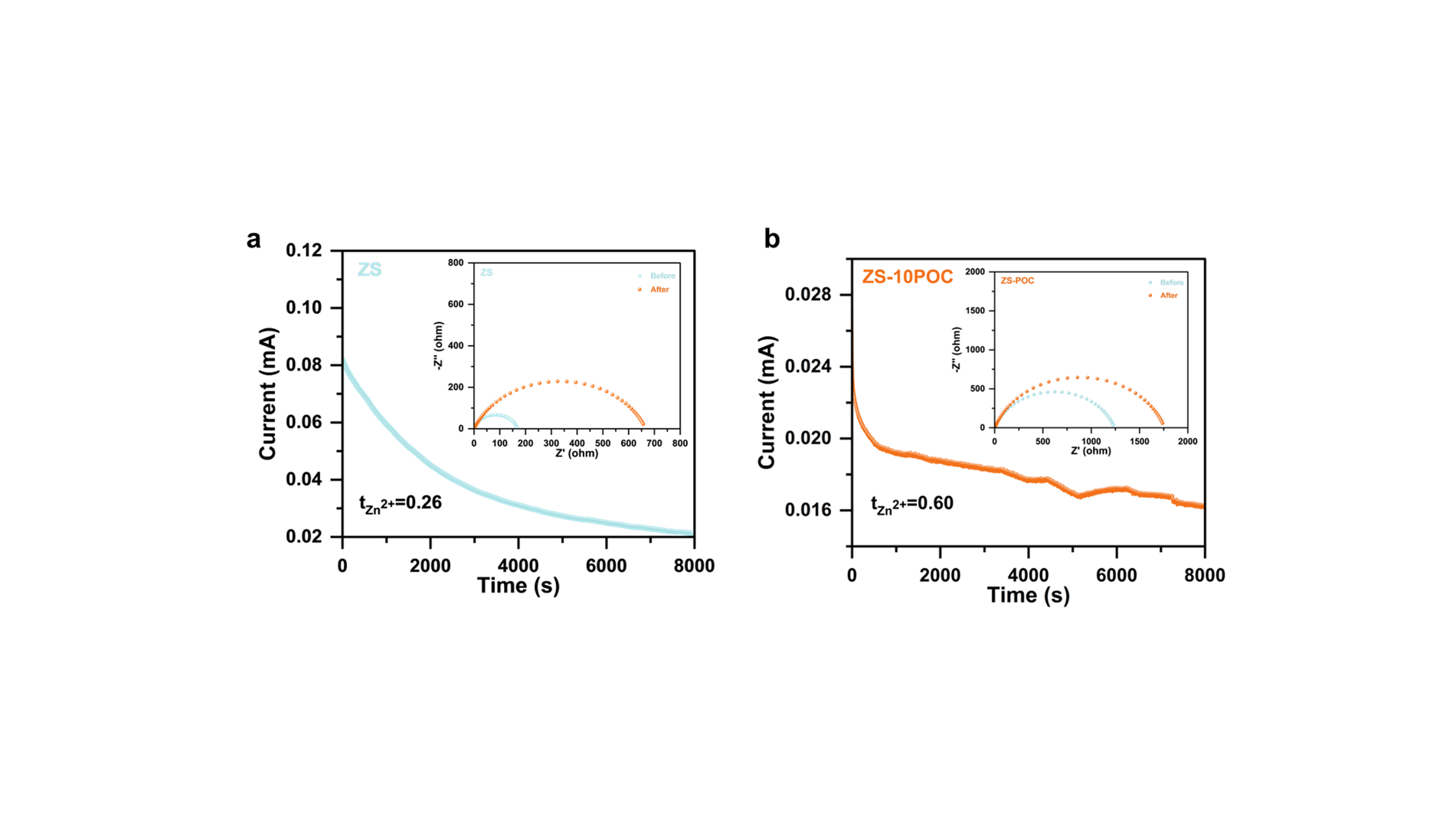


**Fig. S26** Calculation of the transference number of Zn^2+^ ($t_{{Zn}^{2+}}$). **a** i-t curve of Zn//Zn symmetric cell in ZS and **b** ZS-POC electrolyte after 8000 s of polarization at constant potential (20 mV).


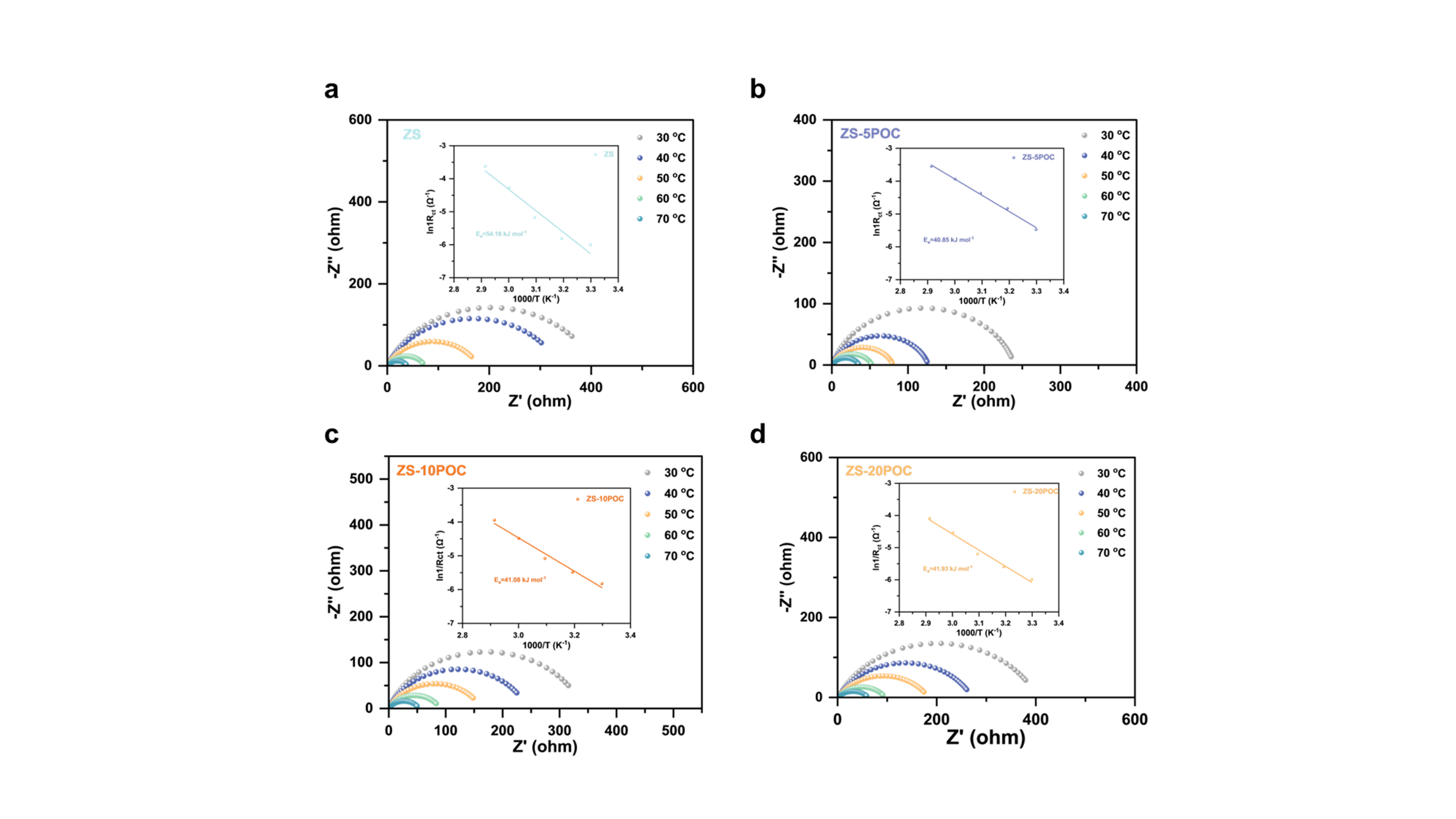


**Fig. S27** Arrhenius curves and comparison of activation energies of **a** ZS, **b** ZS-5POC, **c** ZS-10POC and **d** ZS-20POC electrolytes.


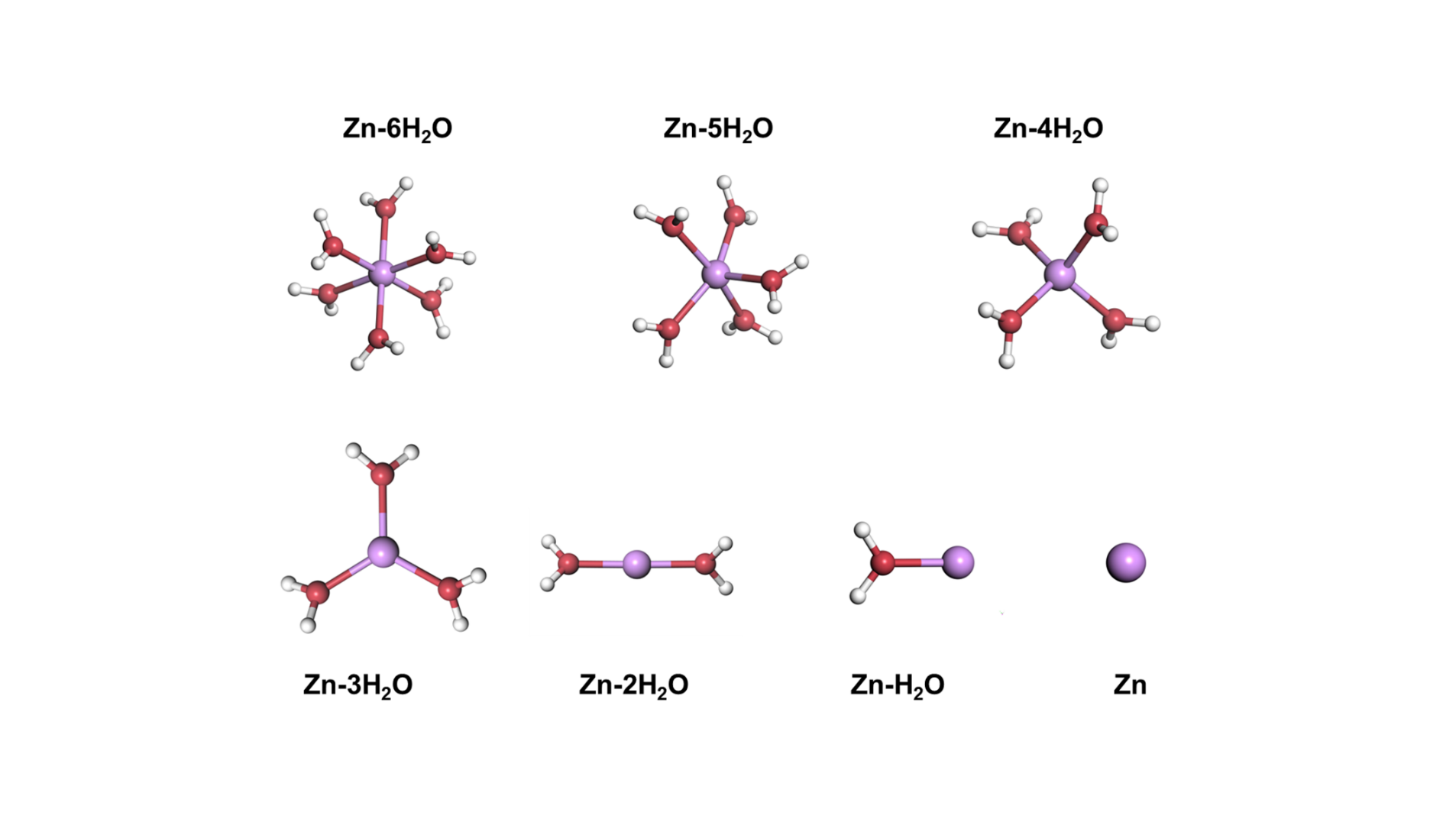


**Fig. S28** Atomic models for Zn ions with different hydrates in the ZS electrolyte.


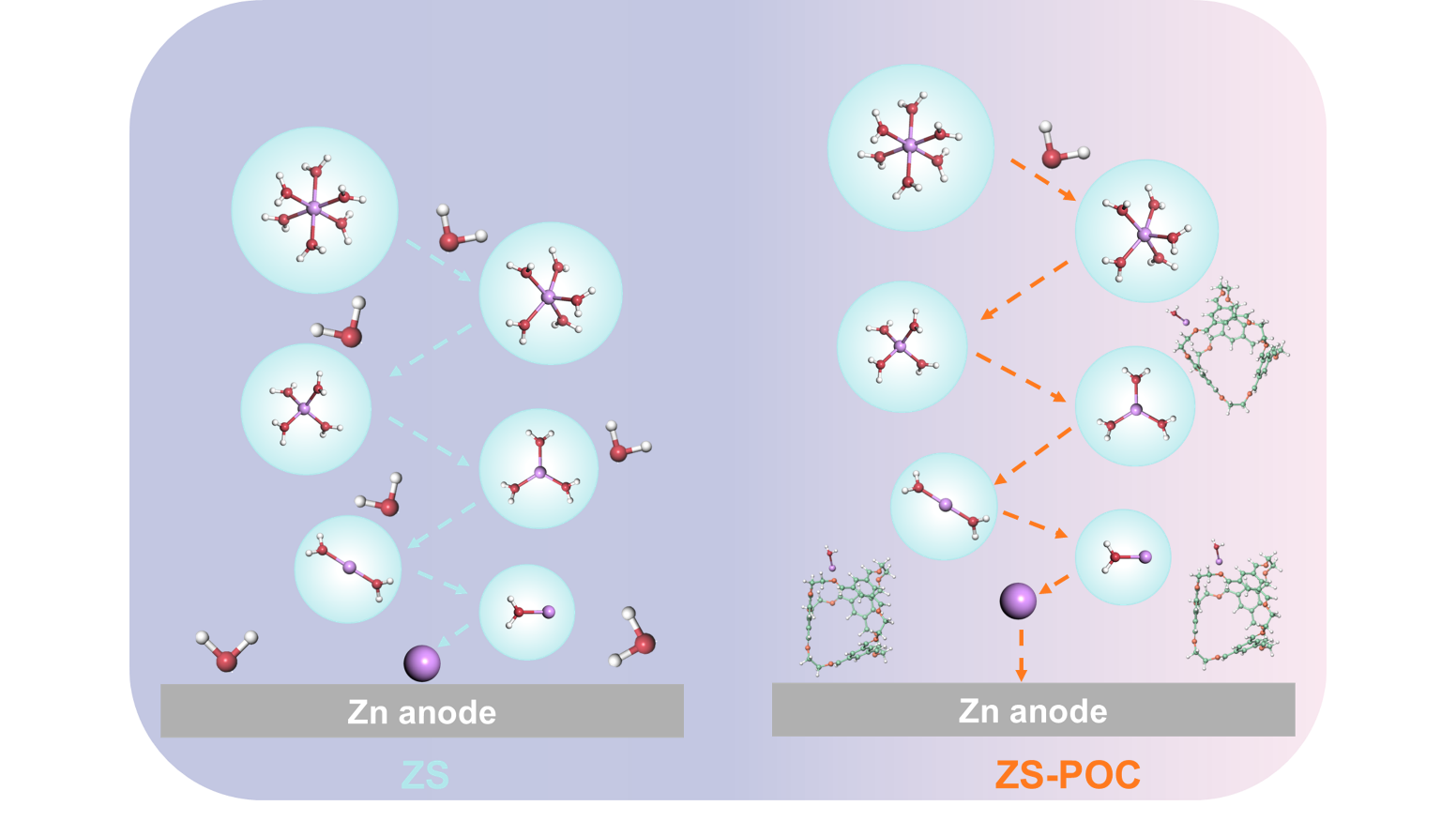


**Fig. S29** Schematic diagram of desolvation in different electrolytes.


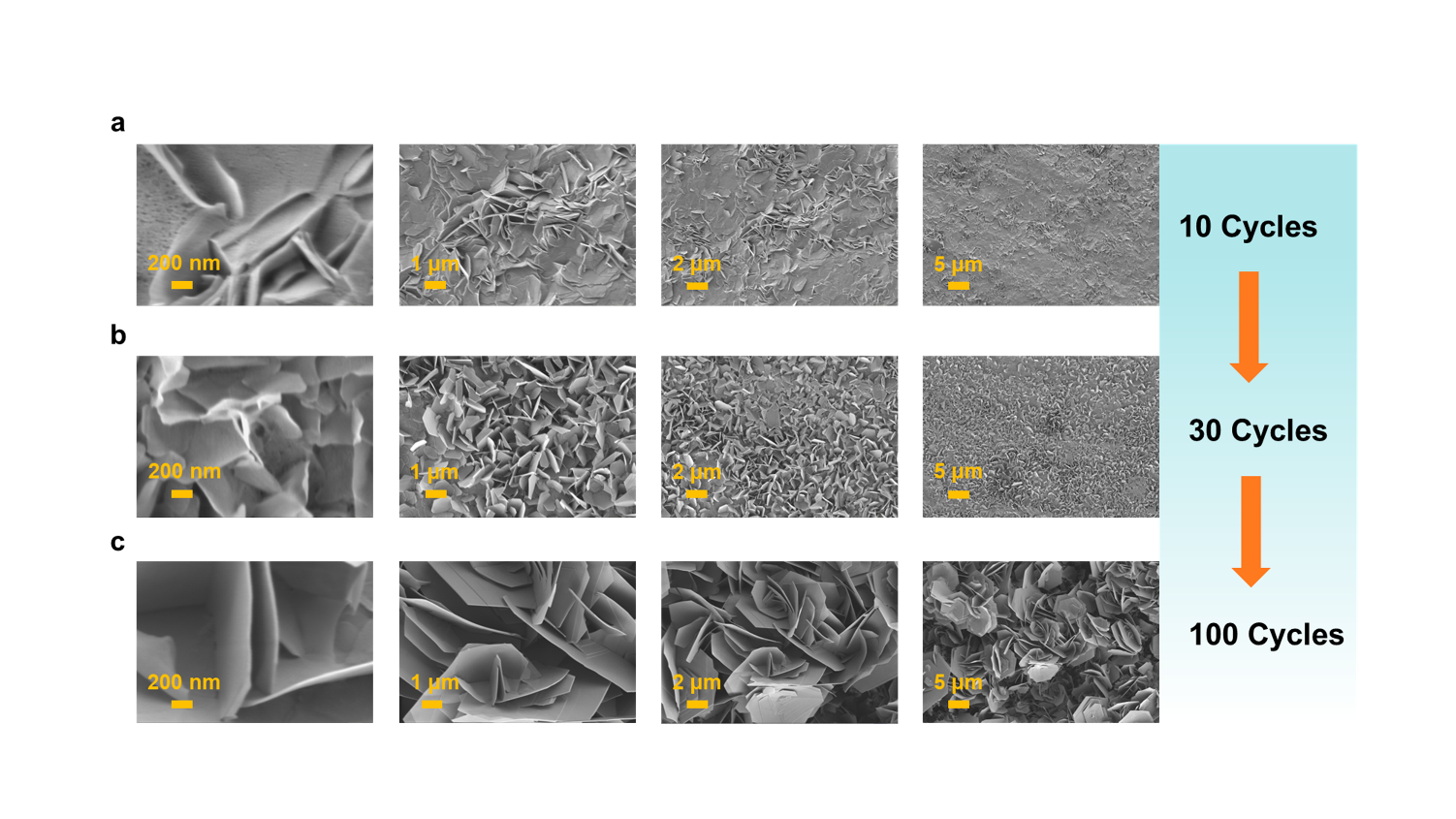


**Fig. S30** SEM images of zinc anode surfaces after cycling in ZS electrolyte.


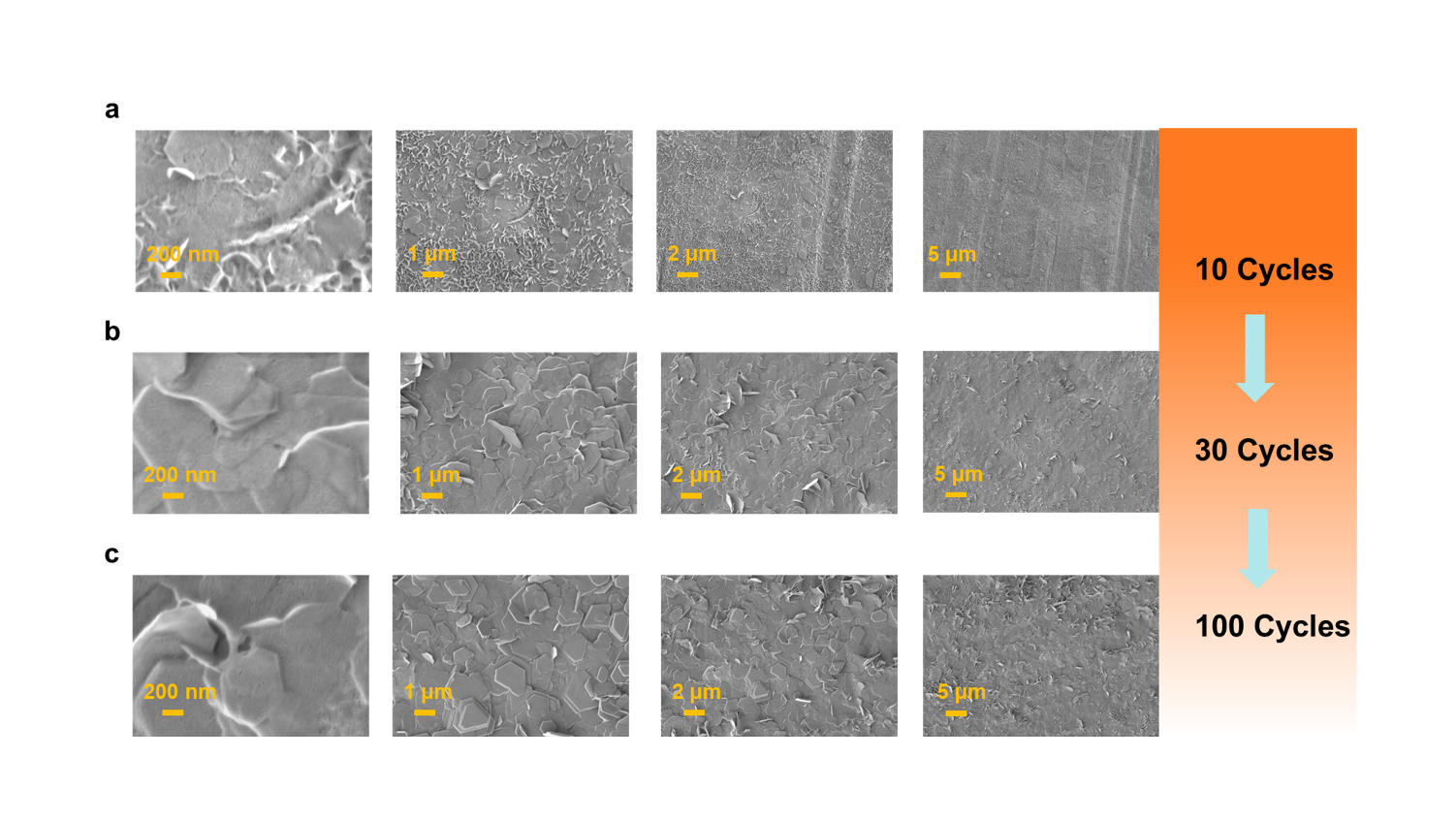


**Fig. S31** SEM images of zinc anode surfaces after cycling in ZS-POC electrolyte.


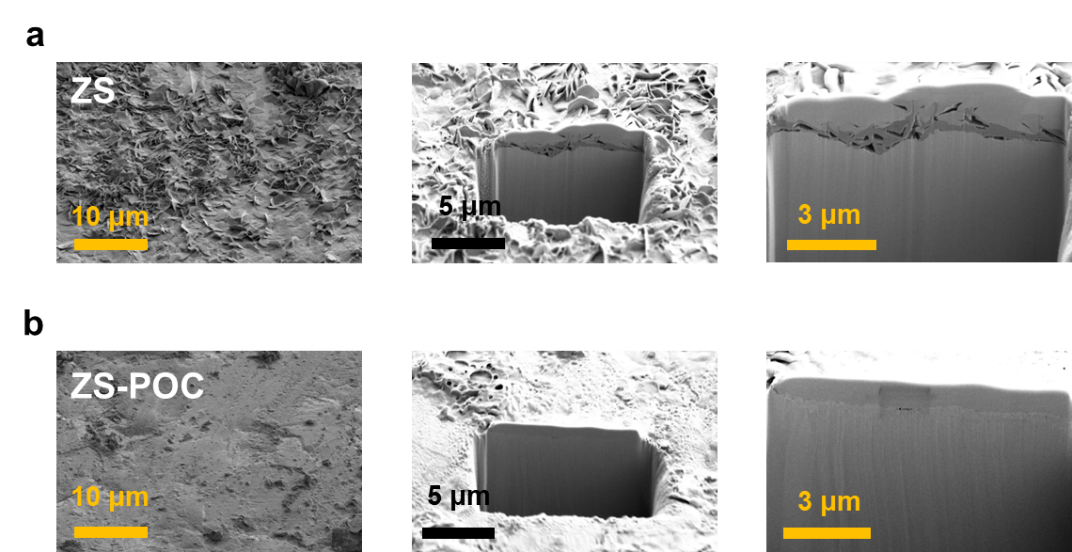


**Fig. S32** Focused Ion Beam Scanning Electron Microscopy (FIB-SEM) images of zinc anode after 100 cycles in ZS and ZS-POC electrolytes.


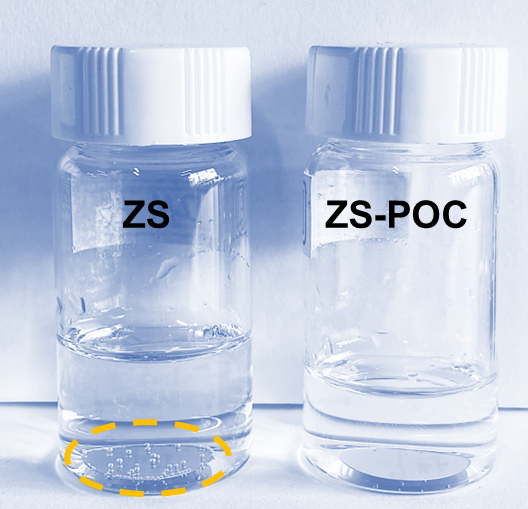


**Fig. S33** Optical image of Zn anode immersion experiment after 7 days.


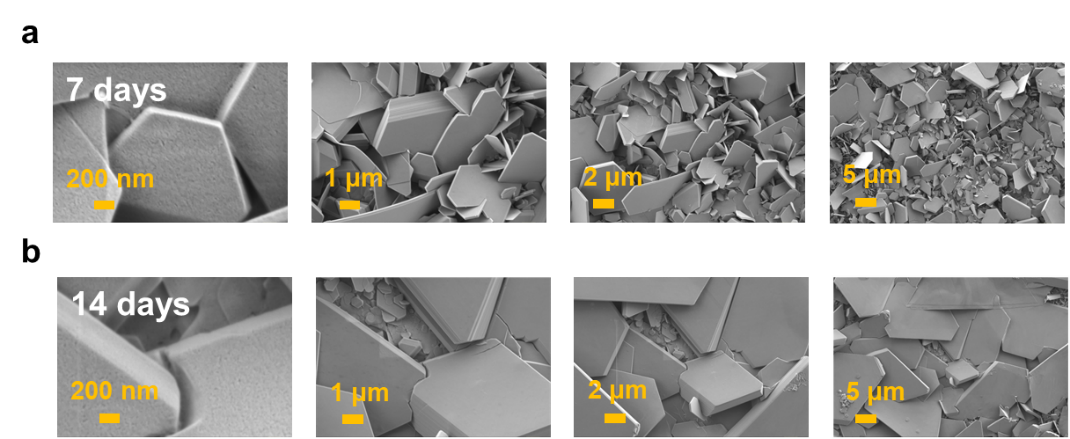


**Fig. S34** SEM images of zinc foil after **a** 7 and **b** 14 days of immersion in ZS electrolyte.


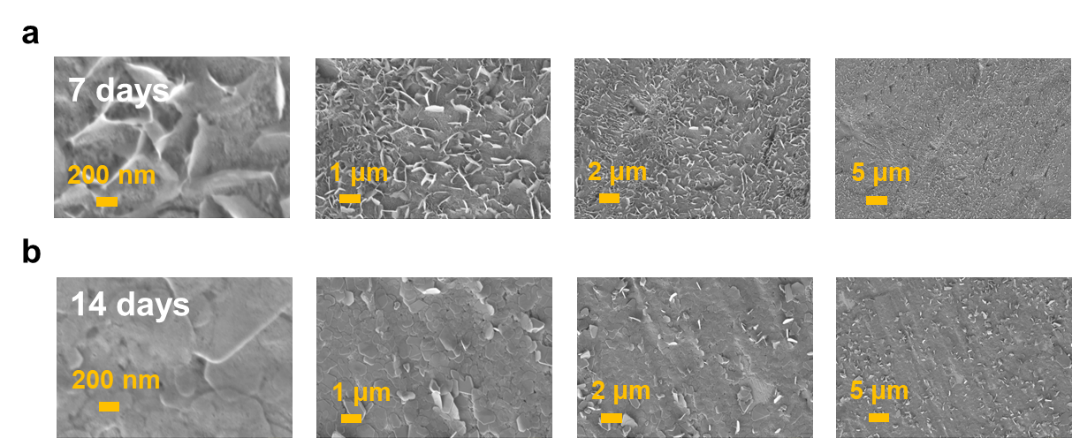


**Fig. S35** SEM images of zinc foil after **a** 7 and **b** 14 days of immersion in the ZS-POC electrolytes.


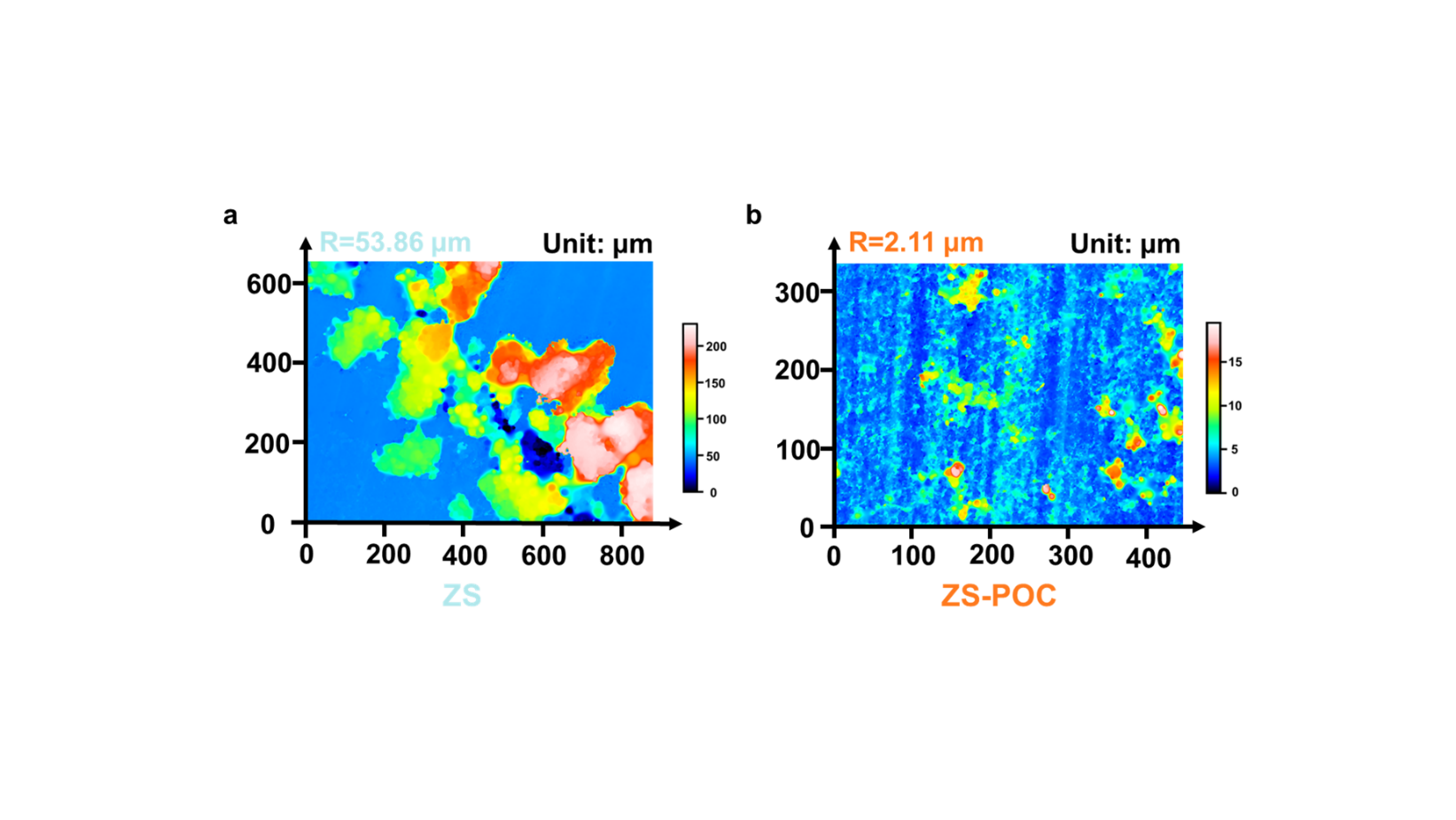


**Fig. S36** CLSM 2D height images of plating Zn in the **a** ZS electrolyte and **b** ZS-POC electrolyte after 60 minutes.


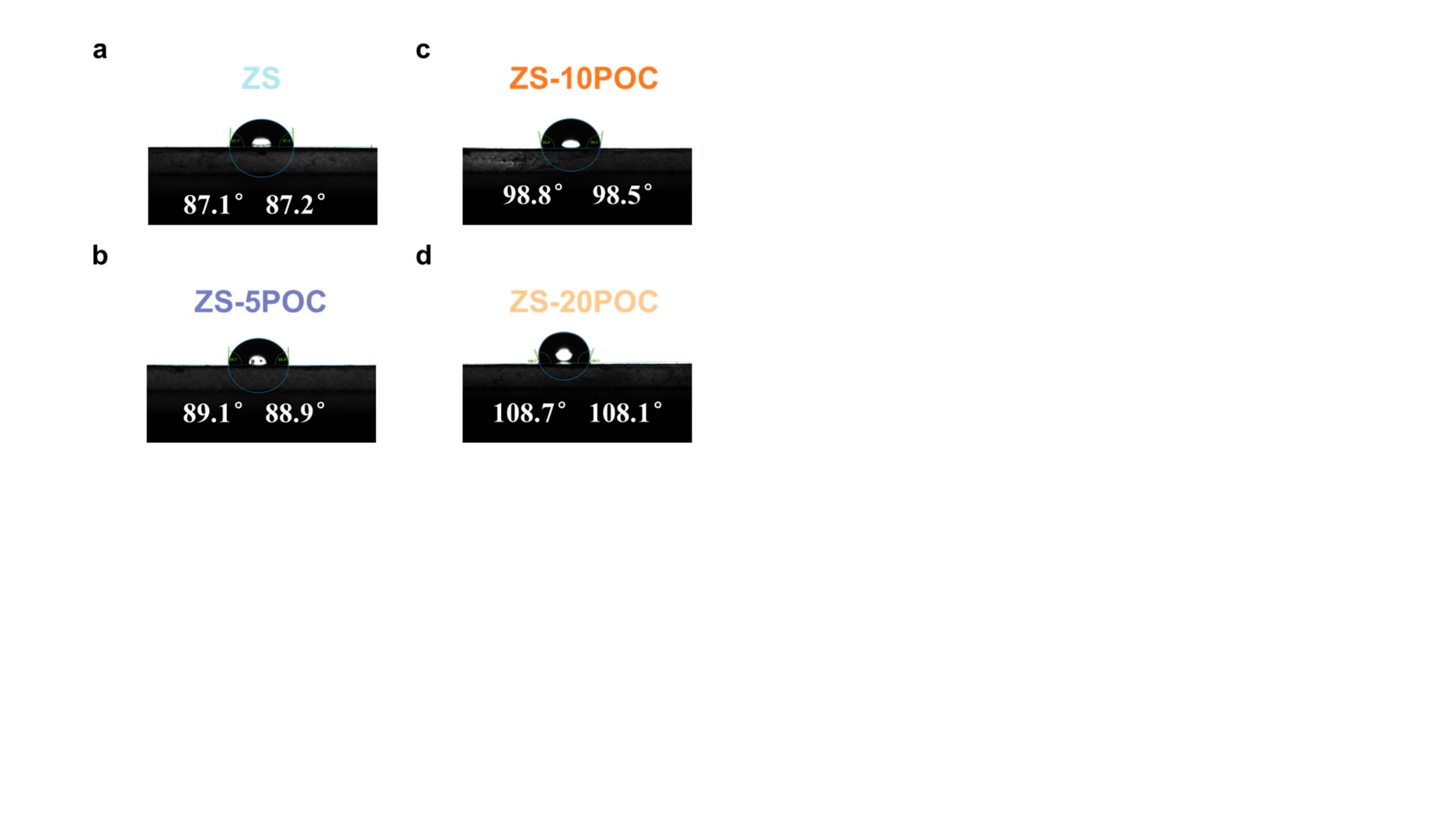


**Fig. S37** Contact angles of the ZS, ZS-5POC, ZS-10POC and ZS-20POC electrolytes.


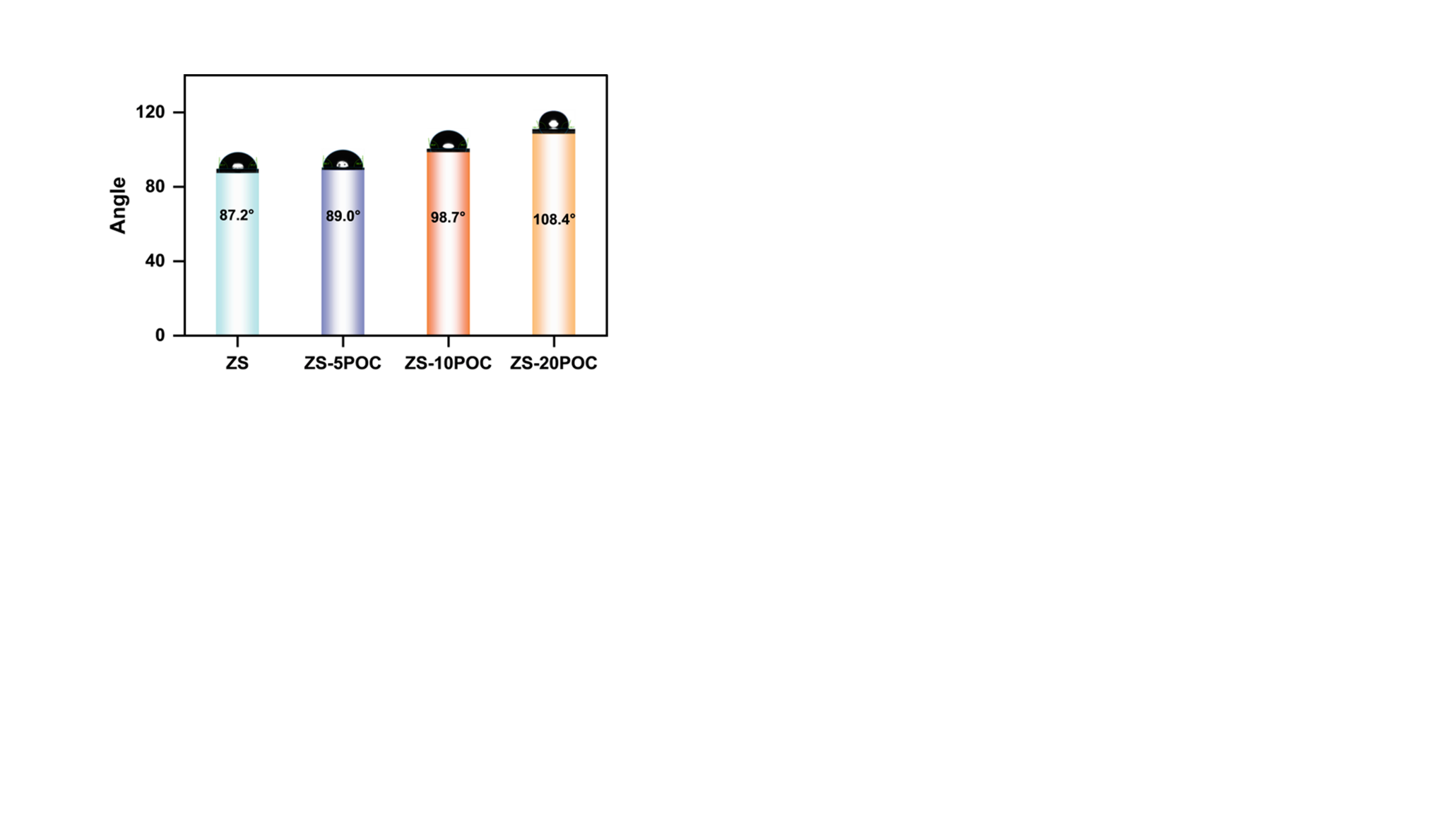


**Fig. S38** Contact angles histograms of the ZS, ZS-5POC, ZS-10POC and ZS-20POC electrolytes.


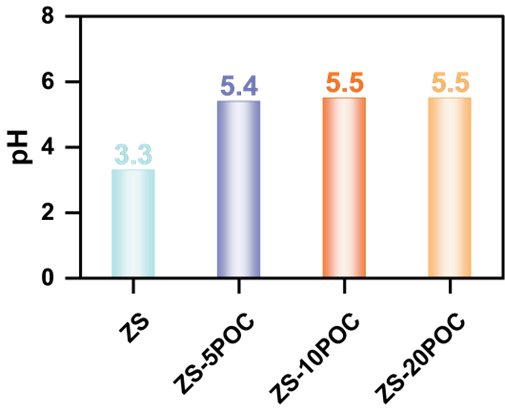


**Fig. S39** The pH of ZS with different POC concentrations.


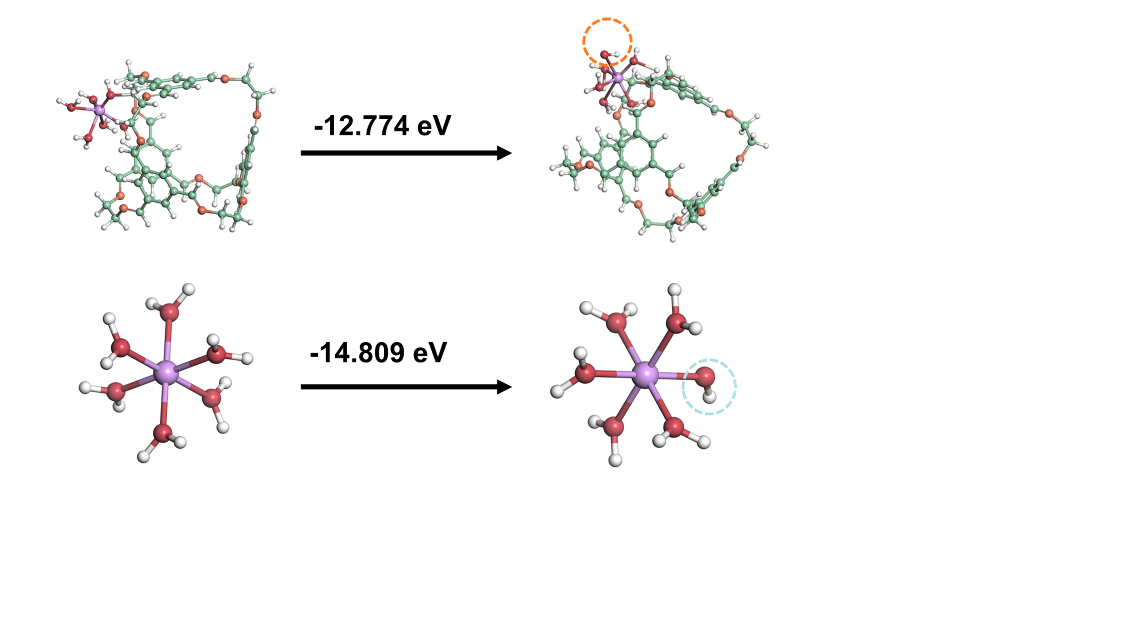


**Fig. S40** The deprotonation (H^+^ dissociation) energy of Zn^2+^ solvation shell in the ZS and ZS-POC electrolytes.


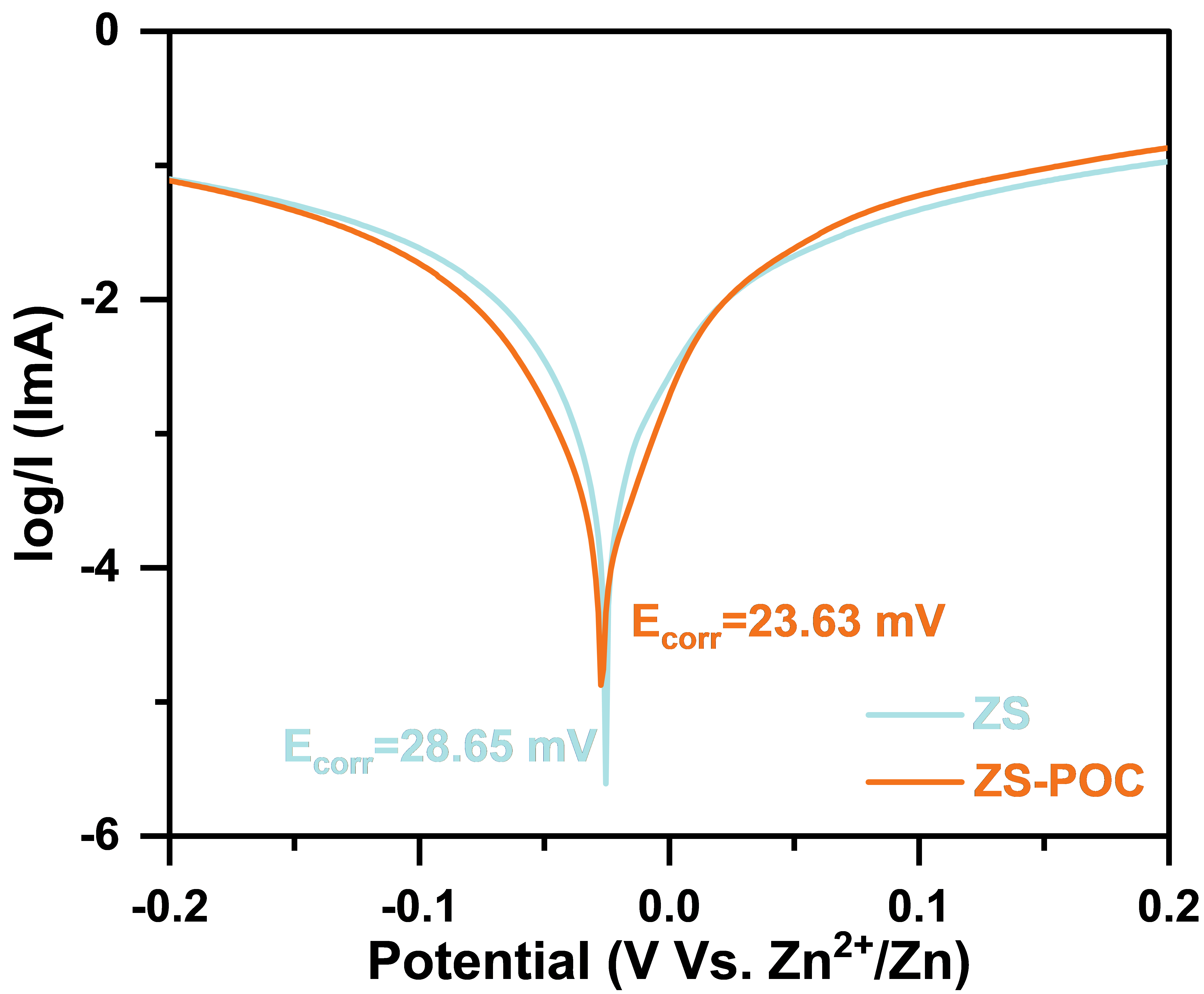


**Fig. S41** Tafel plots of Zn anodes in the ZS and ZS-POC electrolytes.


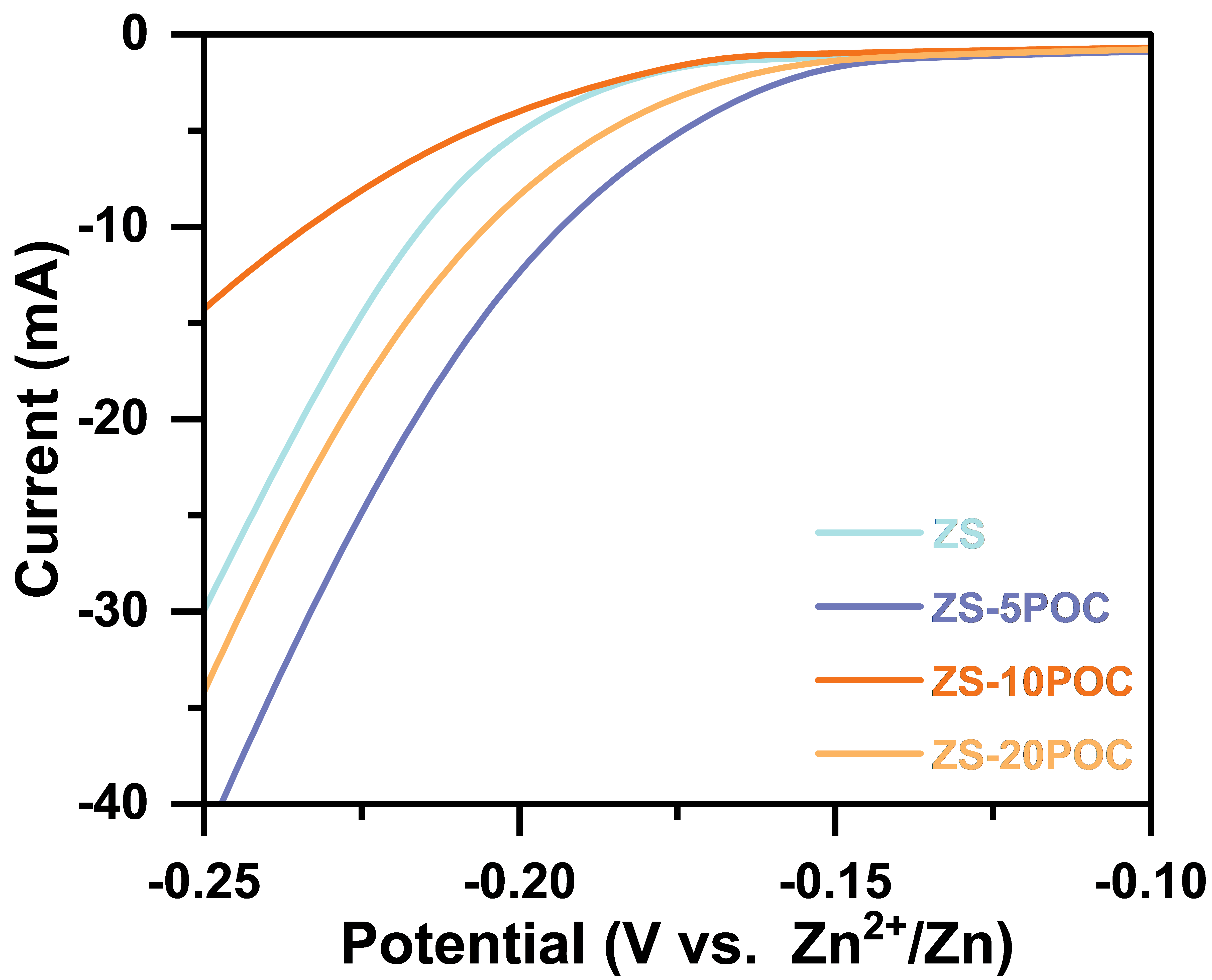


**Fig. S42** LSV curves tested of Zn//Ti asymmetric cells.


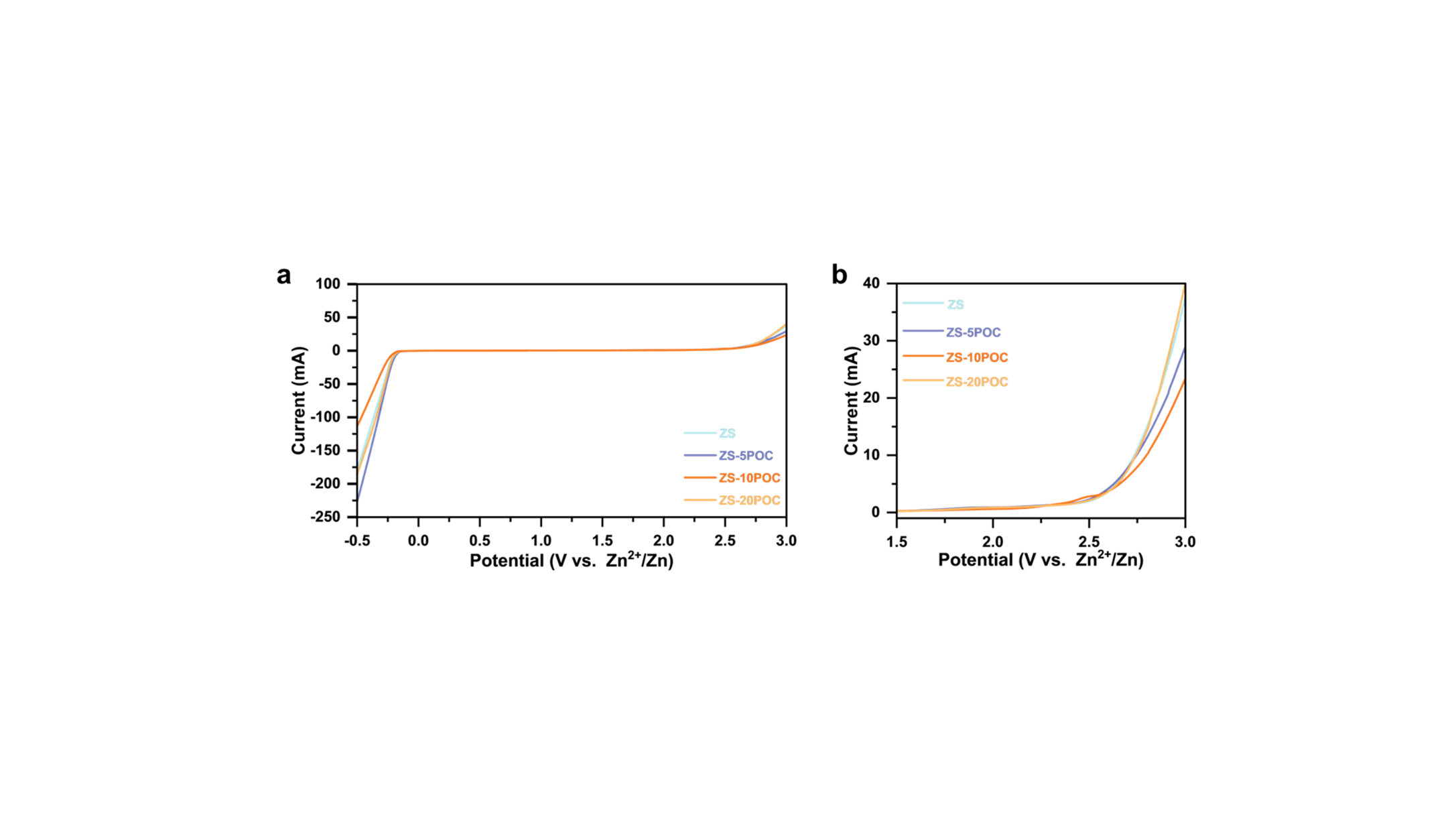


**Fig. S43** LSV of Zn-Ti cells tested in the ZS, ZS-5POC, ZS-10POC and ZS-20POC electrolytes.

**
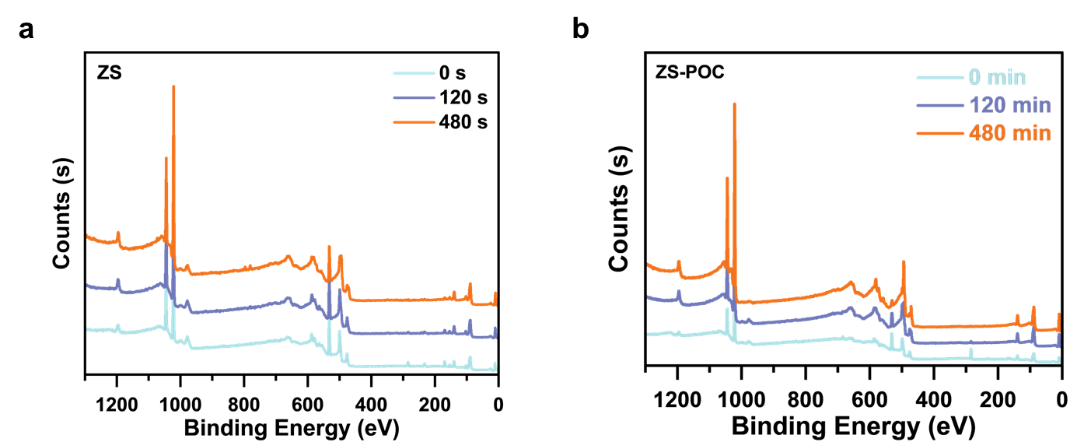
**

**Fig. S44** XPS spectra and depth profiles of zinc anodes after 30 cycles in the **a** ZS and **b** ZS-POC after 0 min, 2 min and 8 min of Ar^+^ sputtering.


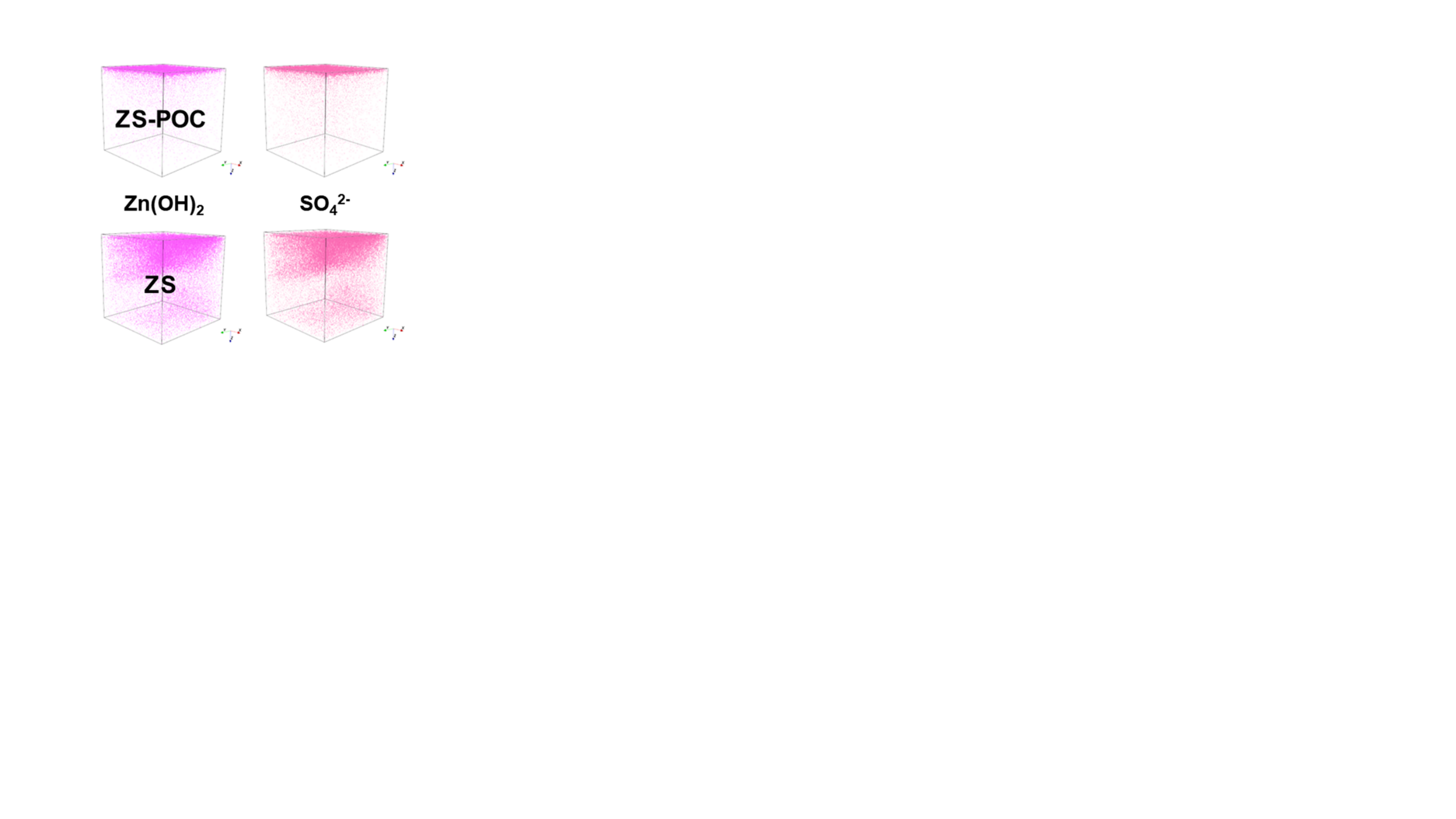


**Fig. S45** 3D spatial distribution of surface elements of the zinc anode after 30 cycles in different electrolytes via TOF-SIMS.

**

**

**Fig. S46** Galvanostatic Zn plating/stripping in the Zn//Zn symmetric cells with ZS-POC electrolytes with different concentrations at 50 mA·cm^-2^ and 2.5 mA h·cm^-2^.

**
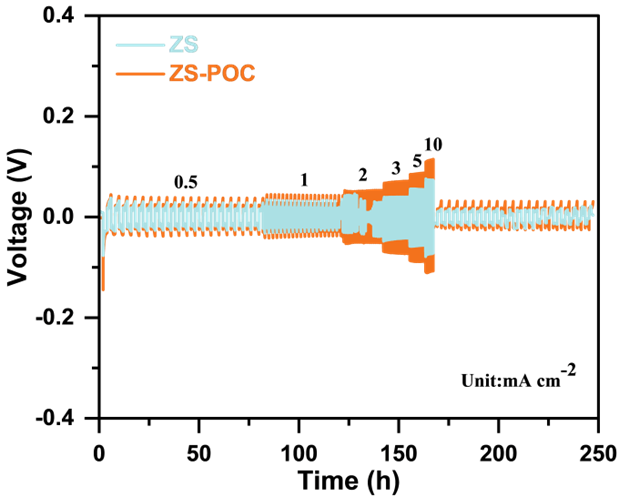
**

**Fig. S47** Effect of the ZS and ZS-POC electrolytes on the multiplicity performance of Zn//Zn symmetric cells.


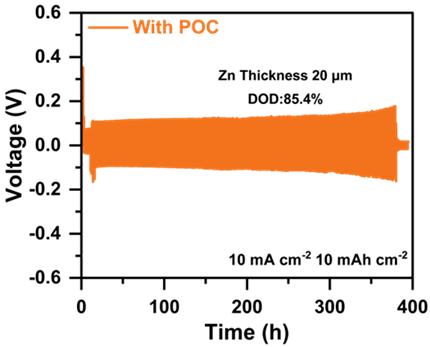


**Fig. S48** Time-voltage profiles with high depth of discharge (DOD) of 85.4% (20 μm Zinc foil);


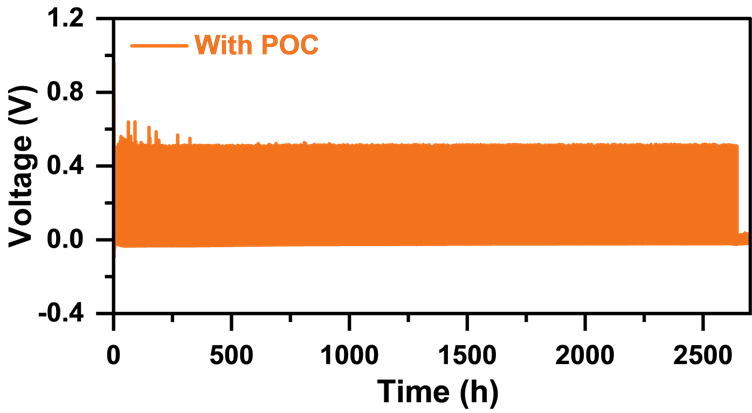


**Fig. S49** Cycling performance of Zn^2+^ plating/stripping of Zn//Cu cells in ZS-POC electrolytes.


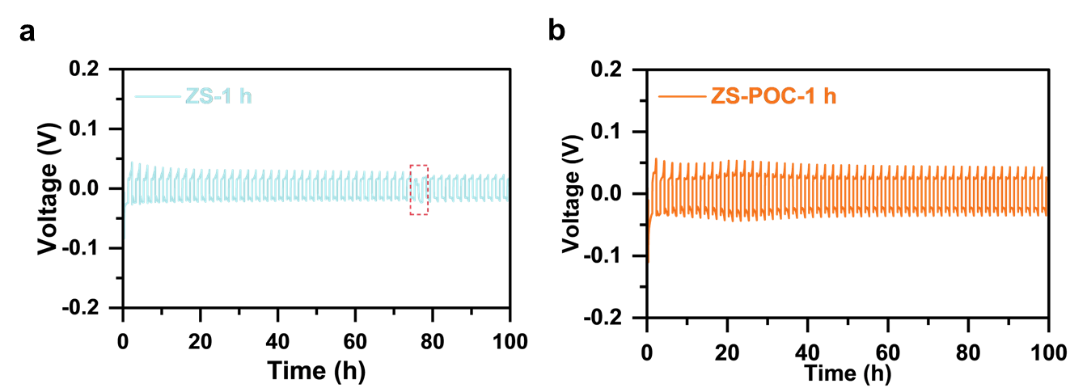


**Fig. S50** The voltage-time profiles of Zn//Zn cells, where the Zn anode was soaked in the **a** ZS and **b** ZS-POC electrolytes for 1 h before battery assembly.


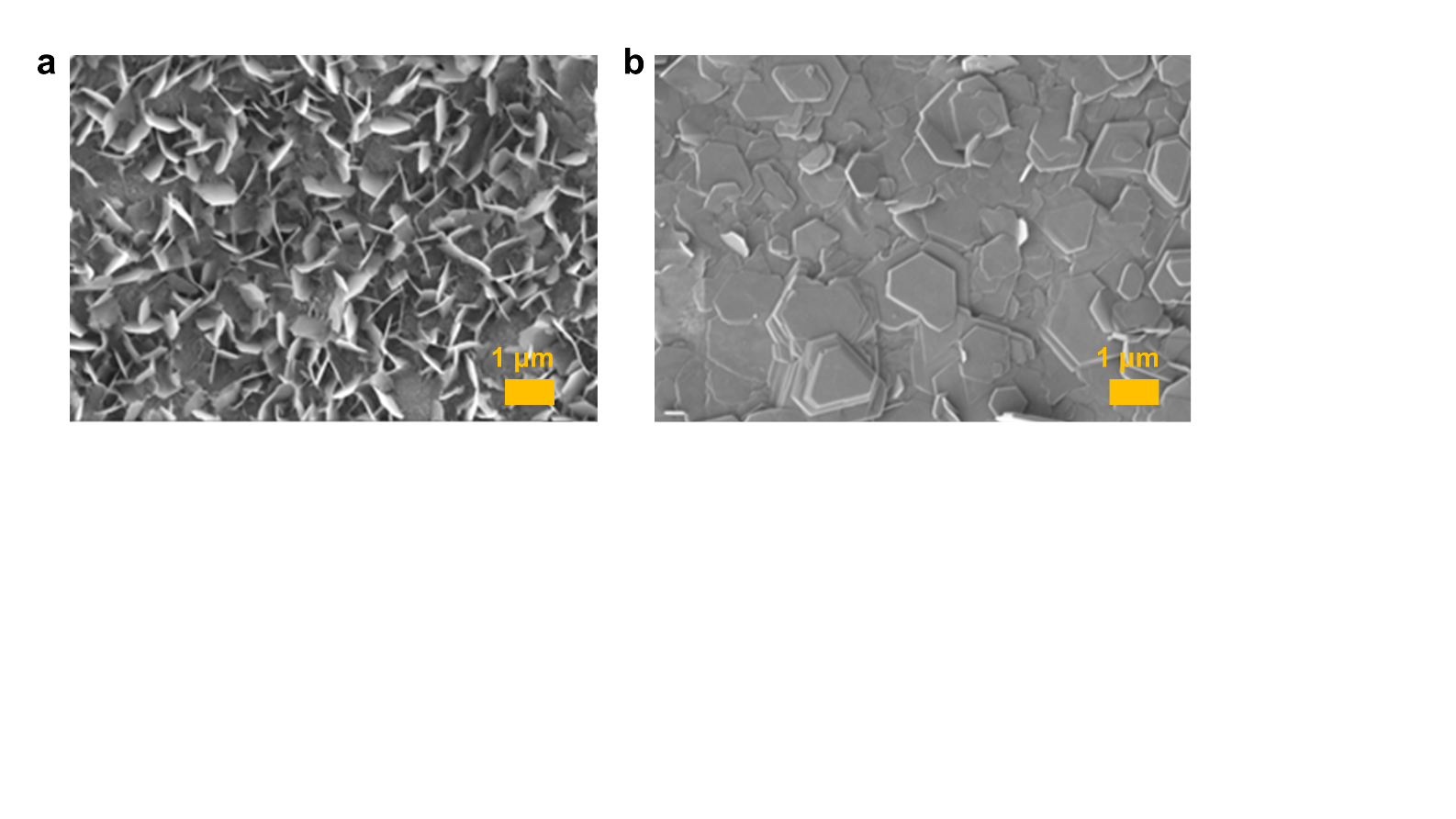


**Fig. S51 a** SEM image of the failed zinc anode in ZS electrolyte; **b** SEM image of the failed zinc anode after 30 cycles in ZS-POC electrolyte.


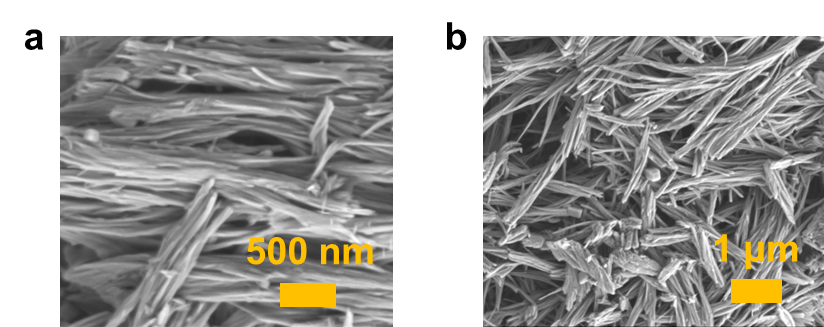


**Fig. 52** SEM images of NVO.


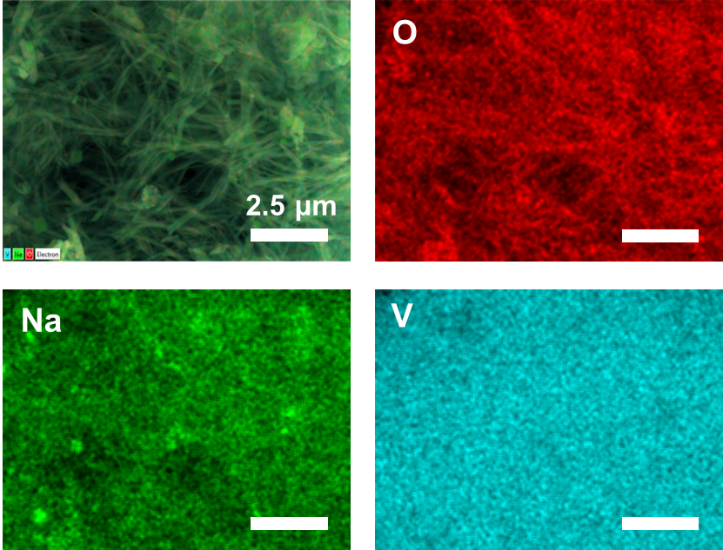


**Fig. S5****3** Mapping images of NVO.


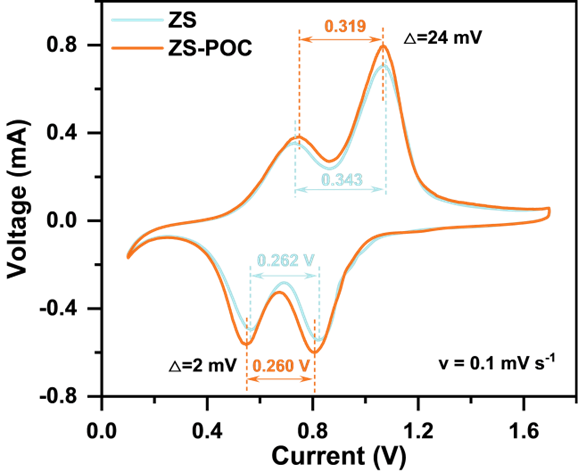


**Fig. S54** CV curves of Zn//NVO full cells in the **a** ZS and **b** ZS-POC.


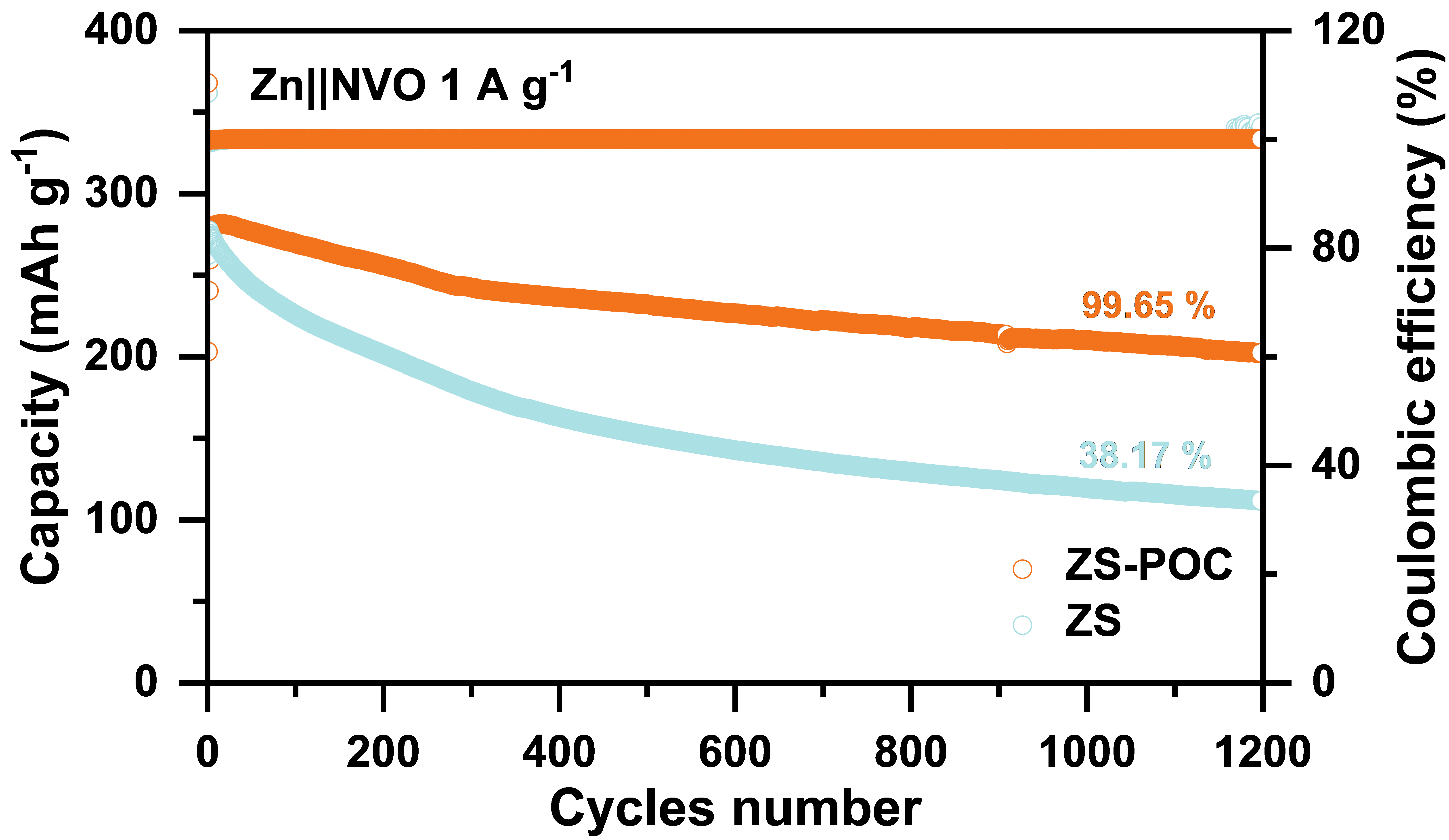


**Fig. S55** Cycling performance of Zn//NVO full cells with the ZS and ZS-POC electrolytes at 1 A g^-1^.


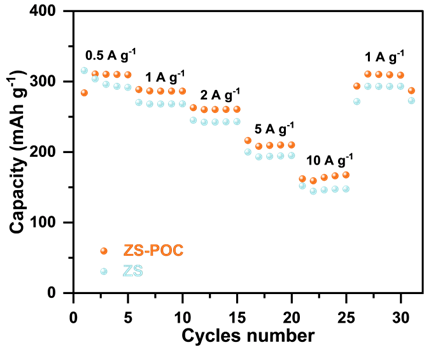


**Fig. S56** Rate capability of Zn//NVO full cells with the ZS and ZS-POC electrolytes at various current densities.


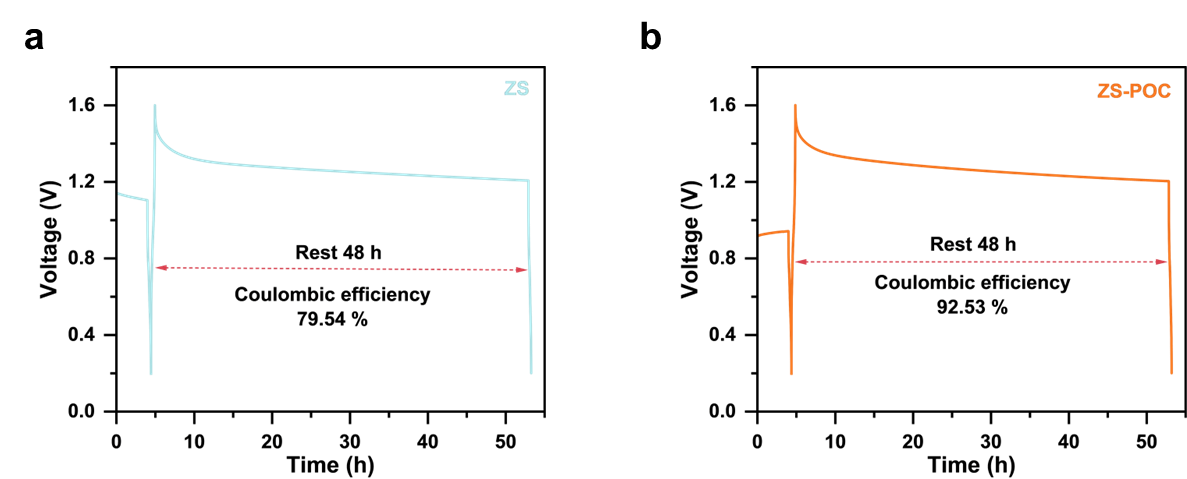


**Fig. S57** Self-discharge curves of full cells with the **a** ZS and **b** ZS-POC electrolytes.

**Supplementary Tables**

**Table S1** Battery performance comparison

| Reference | Electrolytes | Current density  (mA cm^-2^) | Mass loading  (mg cm^-2^) | Discharge capacity  （Ah） |
| --- | --- | --- | --- | --- |
| [S12] | S5 | 6.3 | 9.08 | - |
| [S13] | HE | 5 | 1-2 | 0.00543-0.0109 |
| [S14] | BmBr/ZSO | 1 | 11.85 | 0.06 |
| [S15] | ZSO:ZclO 2:8 DDSE | 30 | 16.4 | 0.237 |
| [S16] | Al-HQ | - | - | 0.75 |
| [S17] | HCDISE | 8 | 10 | 0.033 |
| This work | ZS-POC | 50 | 26.77 | 1.02 |

**Table S2** The specifications of Zn//NVO pouch cell with discharge capacity of 1.02 Ah and sizes of 15 cm× 20 cm

|  | Parameter | Value |
| --- | --- | --- |
| NVO cathode | Discharge capacity | 235.87 mAh/g at 0.5 A/g |
|  | Mass loading of active material | 70% |
|  | Area weight | 26.77 mg/cm^2^ |
|  | Area capacity | 4.42 mAh/cm^2^ |
|  | Number of layers | 1 |
| Binder | Type | PTFE |
| Zn anode | Specific capacity | 820 mAh/g |
|  | Zn thickness | 20 μm |
|  | Area capacity | 11.7 mAh/cm^2^ |
|  | N/P ratio | 2.65 |
| Electrolyte | Type | ZS-POC |
| Separator | Type | Whatman GF/D |
|  | Thickness | 47 μm |
|  | Area Weight |  |
| Pouch Cell | Capacity | 1.02 Ah |
|  | Size | 15 cm × 20 cm |

**Supplementary References**

1. Z. Qu, Y. Muhammad, W. He, J. Li, Z. Gao et al., Designing C-Fe-O bonded MIL-88B(Fe)/jasmine petal-derived-carbon composite biosensor for the simultaneous detection of dopamine and uric acid. Chem. Eng. J. **404**, 126570 (2021). <https://doi.org/10.1016/j.cej.2020.126570>
2. Y. Wang, X. Huang, X. Zhang, Ultrarobust, tough and highly stretchable self-healing materials based on cartilage-inspired noncovalent assembly nanostructure. Nat. Commun. **12**(1), 1291 (2021). <https://doi.org/10.1038/s41467-021-21577-7>
3. X.-Q. Zhang, X. Chen, X.-B. Cheng, B.-Q. Li, X. Shen et al., Highly stable lithium metal batteries enabled by regulating the solvation of lithium ions in nonaqueous electrolytes. Angew. Chem. Int. Ed. **57**(19), 5301–5305 (2018). <https://doi.org/10.1002/anie.201801513>
4. G. Kresse, J. Furthmüller, Efficient iterative schemes for *ab initio* total-energy calculations using a plane-wave basis set. Phys. Rev. B **54**(16), 11169–11186 (1996). <https://doi.org/10.1103/physrevb.54.11169>
5. P.E. Blöchl, P. Margl, K. Schwarz, Ab initio molecular dynamics with the projector augmented wave method. In: Chemical Applications of Density-Functional Theory.. pp. 54–69. American Chemical Society, (1996). <https://doi.org/10.1021/bk-1996-0629.ch004>
6. J.P. Perdew, K. Burke, M. Ernzerhof, Generalized gradient approximation made simple. Phys. Rev. Lett. **77**(18), 3865–3868 (1996). <https://doi.org/10.1103/physrevlett.77.3865>
7. J. Klimeš, D.R. Bowler, A. Michaelides, Chemical accuracy for the van der Waals density functional. J. Phys.: Condens. Matter **22**(2), 022201 (2010). <https://doi.org/10.1088/0953-8984/22/2/022201>
8. J. Klimeš, D.R. Bowler, A. Michaelides, Van der Waals density functionals applied to solids. Phys. Rev. B **83**(19), 195131 (2011). <https://doi.org/10.1103/physrevb.83.195131>
9. G. Kresse, D. Joubert, From ultrasoft pseudopotentials to the projector augmented-wave method. Phys. Rev. B **59**(3), 1758–1775 (1999). <https://doi.org/10.1103/physrevb.59.1758>
10. M. Wang, Y. Meng, M. Sajid, Z. Xie, P. Tong et al., Bidentate coordination structure facilitates high-voltage and high-utilization aqueous Zn-I(2) batteries. Angew. Chem. Int. Ed. **63**(39), e202404784 (2024). <https://doi.org/10.1002/anie.202404784>
11. B. Xie, C. Zheng, H. Lang, M. Li, Q. Hu et al., Ultrastable electrolyte (>3500 hours at high current density) achieved by high-entropy solvation toward practical aqueous zinc metal batteries. Energy Environ. Sci. **17**(19), 7281–7293 (2024). <https://doi.org/10.1039/d4ee02896a>
12. Y. Q. Wang, Y. T. Zhu, H. Xian, B. Wang, Z. Chen, X. Tan, Z. Z. Wu, S. L. Liu, J. F. Mao, S. Q. Zhang, Z. P. Guo, L. Z. Wang, C. J. Hawker, A. K. Whittaker, C. Zhang. Amphiphilic fluorinated block copolymer additives for ultrastable aqueous Zn-ion batteries. J Am Chem Soc. **148**(1), 448–460 (2026). <https://doi.org/10.1021/jacs.5c14364>
13. L. Lin, Z. P. Shao, S. Z. Liu, P. Yang, K. P. Zhu, W. B. Zhuang, C. W. Li, G. D. Guo, W. H. Wang, G. Hong, B. Wu, Q. C. Zhang, Y. G. Yao. High-entropy aqueous electrolyte induced formation of water-poor Zn solvation structures and gradient solid-electrolyte interphase for long-life Zn-metal anodes. Angew Chem, IntEd. **64**(15), e202425008 (2025). <https://doi.org/10.1002/anie.202425008>
14. Y. Q. Lv, C. Y. Huang, M. Zhao, M. Z. Fang, Q. W. Dong, W. Q. Tang, J. T. Yang, X. X. Zhu, X. J. Qiao, H. F. Zheng, C. Sun, L. J. Zheng, M. T. Zheng, Y. K. Xu, J. Lu. Synergistic anion-cation chemistry enables highly stable Zn metal anodes. J Am Chem Soc. **147**(10), 8523–8533 (2025). <https://doi.org/10.1021/jacs.4c16932>
15. G. J. Li, Q. Q. Cai, S. L. Zhang, J. A. Yuwono, L. Mao, H. Y. Jin, Z. P. Guo. Decoupled dual-salt electrolyte for practical aqueous zinc batteries. Nature Sustainability. **8**(11), (2025). <https://doi.org/10.1038/s41893-025-01646-1>
16. W. J. Fan, S. Y. Tian, L. P. Qin, T. S. Alomar, P. C. Ruan, Z. M. El-Bahy, N. AlMasoud, B. G. Lu, J. Zhou. Inner-sphere electron transfer enabling highly reversible mn/mno conversion toward energy-dense electrolytic zinc-manganese batteries. J Am Chem Soc. **147**(22), 18694–18703 (2025). <https://doi.org/10.1021/jacs.5c01648>
17. Y. Y. Ji, Q. Hu, J. X. Zhao, C. P. Han, H. M. Cheng. A dynamically ion-sieved electrolyte towards ultralong-lifespan Zn-ion batteries. Angew Chem, IntEd. **64**(1), e202412853 (2025). <https://doi.org/10.1002/anie.202412853>
